# The Redox-Active Tyrosine Is Essential for Proton Pumping in Cytochrome c Oxidase

**DOI:** 10.3389/fchem.2021.640155

**Published:** 2021-04-14

**Authors:** Margareta R. A. Blomberg

**Affiliations:** Arrhenius Laboratory, Department of Organic Chemistry, Stockholm University, Stockholm, Sweden

**Keywords:** cytochrome c oxidase, energy conservation, proton pumping, redox-active tyrosine, midpoint potentials, density functional theory

## Abstract

Cellular respiration involves electron transport via a number of enzyme complexes to the terminal Cytochrome *c* oxidase (C*c*O), in which molecular oxygen is reduced to water. The free energy released in the reduction process is used to establish a transmembrane electrochemical gradient, via two processes, both corresponding to charge transport across the membrane in which the enzymes are embedded. First, the reduction chemistry occurring in the active site of C*c*O is electrogenic, which means that the electrons and protons are delivered from opposite sides of the membrane. Second, the exergonic chemistry is coupled to translocation of protons across the entire membrane, referred to as proton pumping. In the largest subfamily of the C*c*O enzymes, the A-family, one proton is pumped for every electron needed for the chemistry, making the energy conservation particularly efficient. In the present study, hybrid density functional calculations are performed on a model of the A-family C*c*Os. The calculations show that the redox-active tyrosine, conserved in all types of C*c*Os, plays an essential role for the energy conservation. Based on the calculations a reaction mechanism is suggested involving a tyrosyl radical (possibly mixed with tyrosinate character) in all reduction steps. The result is that the free energy released in each reduction step is large enough to allow proton pumping in all reduction steps without prohibitively high barriers when the gradient is present. Furthermore, the unprotonated tyrosine provides a mechanism for coupling the uptake of two protons per electron in every reduction step, i.e. for a secure proton pumping.

## Introduction

**FIGURE 1:**
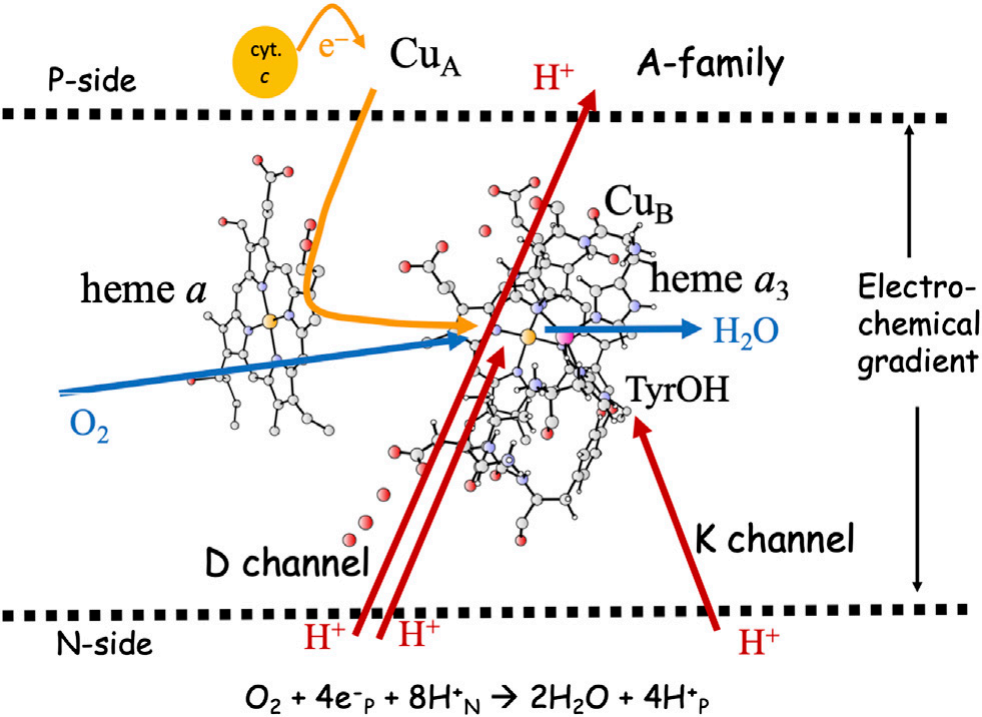
Overview of the A-family C*c*Os. The BNC active site cofactors: high-spin heme *a*_3_, Cu_*B*_ and the cross-linked tyrosine are embedded in the inner mitochondrial or the bacterial membrane. Soluble cytochrome *c*, the electron transfer cofactors (Cu_*A*_ and low-spin heme *a*), plus proton channels (D and K) from the N-side of the membrane to the BNC are indicated. Electron and proton uptake to the BNC is indicated as arrows, as well as the proton pumping across the entire membrane.

Cytochrome *c* oxidase (C*c*O), the terminal enzyme in the respiratory chain, reduces molecular oxygen to water in an exergonic process. A significant part of the released free energy is converted into an electrochemical gradient across the membrane in which the enzyme is embedded. The gradient is used by another enzyme in the same membrane to transform ADP to ATP, which provides energy for many processes in the living cell. As shown in Figure 1 the electrons needed for the oxygen reduction chemistry are delivered from soluble cytochrome *c* located on the P-side of the membrane, while the protons are delivered from the opposite side, the N-side. This means that the chemical reaction corresponds to charge motion across the membrane (electrogenic chemistry), which contributes to the creation of the electrochemical gradient. In 1977 Wikström discovered that the oxygen reduction is coupled to translocation of protons across the entire membrane, referred to as proton pumping, which also contribute to the electrochemical gradient, and thereby increases the efficiency of the energy conservation (Wikström, 1977). The largest subgroup of C*c*Os, the A-family, which can be found in both mitochondria and bacteria, is known to pump four protons per oxygen molecule, i.e., one pumped proton per electron. The stoichiometry of the proton pumping is most likely lower in the other C*c*O families, the B- and the C-families (Han, et al., 2011; Von Ballmoos, et al., 2012). The proton pumping in the A family is the focus of the present study.

The active site in the A-family C*c*Os, the binuclear center (BNC), consists of a high-spin heme group of *a*-type, labeled heme *a*_3_, a copper complex, labeled Cu_*B*_ and a redox-active tyrosine cross-linked to one of the histidine ligands on Cu_*B*_. The electrons are delivered from soluble cytochrome *c* on the P-side of the membrane to the BNC via two electron transfer cofactors, a copper complex, Cu_*A*_ and a low-spin heme *a*. In the A-family C*c*Os two proton channels originating on the N-side of the membrane have been characterized, the D-channel leading to the center of the BNC, and the K-channel leading to the redox-active tyrosine, see Figure 1. The basic features of the reduction process are well known: Molecular oxygen binds to the reduced state of the BNC, the **R** state, with Fe_a3_(II)-Cu_*B*_(I)-TyrOH. The O-O bond is cleaved in the first step, yielding the **P**_*M*_ state, with Fe_a3_(IV)=O-Cu_*B*_(II)OH-TyrO^•^. The rest of the catalytic cycle consists of four proton coupled reduction steps leading back to the **R** state plus two new water molecules. Two or three protons for the chemistry are delivered via the D-channel, and the rest (one or two) via the K-channel. All protons to be pumped use the D-channel. It is now generally agreed that each reduction step in the A-family is coupled to proton pumping, also at a high gradient (Bloch et al., 2004; Ferguson, 2010; Kaila et al., 2010; Wikström and Hummer, 2012; Wikström et al., 2018). Although considerable knowledge about the proton pumping has been achieved (Brzezinski and Gennis, 2008; Popovic et al., 2010; Han et al., 2011; Blomberg and Siegbahn, 2015a; Rich, 2017; Wikström et al., 2018), the mechanistic details are still not agreed on. One of the remaining issues concerns the thermodynamics of the individual reduction steps. The exergonicity of a reduction step is determined by the proton coupled reduction potential (midpoint potential) of the active site cofactor that is reduced in the particular step. Experimental results indicate that two of the four BNC reduction potentials (Cu_*B*_(II) and Fe_a3_(III)) are too low to afford proton pumping (Wikström and Morgan, 1992; Kaila et al., 2010). A second remaining issue concerns how to achieve a secure coupling between the transfer of *one* electron to the BNC active site and the uptake of *two* protons, one for the chemistry and one to be pumped. An important element of such a coupling is the suggested proton loading site (PLS), a position in the vicinity of the BNC where the protons to be pumped are temporarily stored (Morgan et al., 1994; Rich et al., 1996). A third remaining issue concerns the gating of the protons, i.e. how to force the protons to move against the gradient and to avoid that they move back to the N-side, which is thermodynamically favorable as soon as there is a gradient across the membrane.

The focus of the present study is on the first two issues described in the preceding paragraph, the energetics of the individual reduction steps and the coupling of electron and proton uptake. Quantum chemical calculations, using hybrid density functional theory (DFT), have been performed on a model of the BNC active site. To describe the overall energetics of each reduction step, both an electron and a proton are added to each main intermediate of the catalytic cycle. To describe the coupling of electron and proton uptake, the electron affinity of each main intermediate is calculated by adding only an electron. Clearly there are interesting details of the reduction processes which are not possible to describe with the present approach, but those are out of the scope of the present investigation. The results obtained with the present model support previous computational results showing that at least three of the BNC cofactors have high enough proton coupled reduction potentials for proton pumping (Blomberg, 2019). A mechanism is suggested, which allows proton pumping in all four reduction steps, and in which the redox-active tyrosine plays a special role (Blomberg, 2016). The computational results are also used to shed further light on a previously suggested mechanism for the coupling between electron and proton uptake, in which the conserved redox-active tyrosine plays an essential role (Blomberg, 2016; Blomberg, 2020a). The gating problem is shortly discussed.

## Computational Details

**FIGURE 2:**
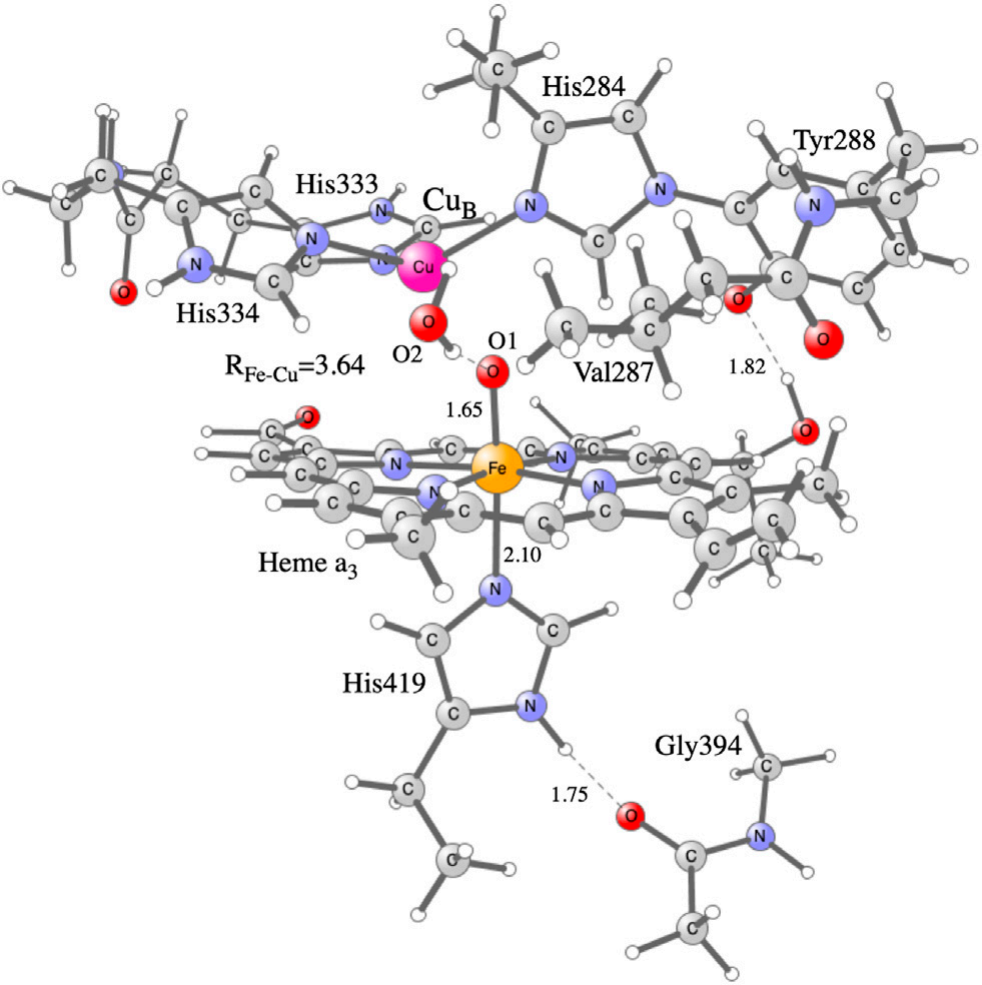
Model of the active site in C*c*O used in the present calculations, showing the optimized **F** state.

Hybrid density functional theory (DFT) is an important tool in the studies of enzymatic reaction mechanisms, in particular employing the cluster approach, as described in recent reviews (Blomberg et al., 2014; Blomberg, 2020a). The cluster model of the active site in C*c*O used in the present study is the same as was used in a recent study on NO reduction in different C*c*Os (Blomberg, 2020b), see Figure 2. It is constructed on the basis of a crystal structure of the *Rhodobacter sphaeroides aa*_3_ C*c*O (Qin et al., 2006). The model has about 170 atoms (depending on the state) and the total charge is +1 for the main intermediates in the catalytic cycle. To obtain the energetics of the catalytic cycle, plausible intermediate structures are optimized and their relative energies calculated. For the energetics of the proton coupled reduction steps the energy cost of an electron from soluble cytochrome *c* and a proton from bulk water is needed. Rather than using calculated values, the total energy of one electron and one proton is given a value that reproduces the overall energy of the reduction process, 2.2 eV, as obtained from experimental reduction potentials of the cytochrome *c* electron donor and the reduction of oxygen to water. This procedure has been described in more detail in several previous papers (Blomberg, 2016; Blomberg, 2019). To obtain the electron affinities, only an electron is added to the different intermediates. The calculated electron affinities are only compared to each other, therefore no explicit energy cost for just an electron is needed in the present context.

The B3LYP*-type of functionals are frequently used to describe metalloenzymes, and are found to generally give reliable results (Blomberg et al., 2014; Siegbahn and Blomberg, 2014). The dispersion corrected hybrid density functional B3LYP-D3 (Becke, 1993; Grimme et al., 2010) was used here, together with a double zeta basis with polarization functions on all second row atoms, to optimize the geometries for the different intermediates. To obtain more accurate single point energies for the optimized structures, the B3LYP*-D3 functional (with 15% exact exchange) (Reiher et al., 2001), the lacv3p+ basis for the metal ions (Jaguar, 2018) and the large cc-pvtz (-f) basis set for the rest of the atoms were used. Polarization effects on the relative energies from the omitted protein that surrounds the cluster model were estimated using a self-consistent reaction field approach with a dielectric constant of 4.0 (Blomberg et al., 1998). For relative energies between intermediates with the same charge, small dielectric effects were obtained. Zero-point corrections for all structures were taken from the Hessians, calculated at the same level as the geometry optimizations, applying the harmonic approximation. The Gaussian 09 package (Gaussian, 2010) was used for geometry optimizations and Hessian calculations. The Jaguar (Jaguar, 2018) program was used for the polarization calculations and for the larger basis set. During the geometry optimizations the coordinates of a few atoms are fixed to the crystal structure, to maintain the steric effects of the surrounding protein, which means that accurate entropy effects cannot be obtained from the Hessians. The frozen atoms are the alpha carbons on each residue together with the two hydrogen atoms replacing the peptide links. An exception is the proximal His419 which has no frozen atoms, since it is kept in place by hydrogen bonding to the Gly394. Entropy effects were added to the relative energies only for the gaseous O_2_ molecule, assuming that the entropy lost on binding is equal to the translational entropy for the free molecule (10.8 kcal/mol at room temperature). This approximation is supported by previous calculations where more explicit estimates of the entropy effects could be made (Blomberg et al., 2004). For the binding enthalpy of a water molecule to bulk water an empirical value of 12 kcal/mol is used, which includes explicit zero point effects, and which is based on experimental thermodynamics and previous experience (Blomberg and Siegbahn, 2015a; Siegbahn, 2018). The reported energetic results are considered as free energies.

The use of the B3LYP* hybrid functional for the main results reported is based on previous experience showing that 15–20% exact exchange gives the best agreement with experiments for the calculated reduction potentials in C*c*O (Siegbahn and Blomberg, 2018), together with a recent study of the accuracy in calculated energy profiles for NO reduction in heme-copper oxidases showing that 15% exact exchange gives slightly better agreement with experiments (Blomberg, 2021). However, although it was showed that the reduction potentials in the heme-copper oxidases varies rather little with the fraction of exact exchange in the hybrid functional, it has been found in several studies on both C*c*O and *c*NOR that too small reduction potentials are obtained for the ferric heme (Blomberg, 2016; Blomberg and Siegbahn, 2016). Therefore a correction of 9 kcal/mol is used for the Fe(III)OH to Fe(II)OH_2_ reduction step, which gives good agreement with experiments for both C*c*O and *c*NOR for the B3LYP^*^ functional (Blomberg, 2019; Blomberg, 2020b). Clearly a corresponding (but opposite) correction has to be applied also on oxidation of the ferrous heme iron, i.e., when the oxygen molecule binds forming a ferric superoxide complex. Since it is expected that the main DFT-problem is concerned with the electronic part of the proton coupled reduction potential, the full correction of 9 kcal/mol is used for the calculated electron affinities of the **A** and **E** intermediates, which involve reduction from Fe(III) to Fe(II). It is important to note that the best computational procedure that is possible to use for the present type of systems, inevitably will have uncertainties of a few kcal/mol in the calculated relative energies, and therefore strong conclusions should not be drawn based on small energy differences.

## Results and Discussion

### Energetics of the Individual Reduction Steps

The driving force of each reduction step in the catalytic cycle of O_2_ reduction in C*c*O is determined by the difference in reduction potential between the BNC cofactor reduced in the particular step, and the ultimate electron donor, cytochrome *c*. It can be noted that the immediate electron donor to the BNC, low-spin heme *a*, has a reduction potential quite similar to cytochrome *c*, which means that the details in the energetics of the electron transfer process between the cofactors can be neglected when the overall energetics of the reduction processes is discussed. In Table 1 the energetics of the reduction steps is summarized. The driving force of each reduction step, ∆G (eV), is obtained both from experimental information about the individual reduction potentials (Kaila et al., 2010), and from the presently calculated proton coupled reduction potentials. The table also includes estimated barriers, ∆G^#^ (eV), for each reduction step, and the effects on the energetics from the presence of the electrochemical gradient across the membrane. To complete the catalytic cycle also the energetics of the binding of molecular oxygen and the O-O bond cleavage are included in the table.

**TABLE 1:** Driving forces, ∆G (eV), for the individual reduction steps in the catalytic cycle, as obtained from experiments (Kaila et al., 2010) and from the present calculations. Note that the first column shows only the cofactor reduced in each particular step, not the full BNC. Rate-limiting barriers, ∆G^#^ (eV), estimated from experimental information are also given for each step. The effects on the driving forces and the barrier heights of the presence of the electrochemical gradient across the membrane are also given.

	No gradient			With gradient: 0.2 V Four protons pumped	
Reduction process	∆G (eV) exp	∆G (eV) Calc	∆G^#^ (eV) est	∆G (eV) calc>	∆G^#^ (eV) est
**P**_*M*_→**F**: TyrO^•^ → TyrOH **F** → **O**_*H*_: Fe_*a*3_(IV)=O → Fe(III)OH **O**_*H*_→ **E**: Cu_*B*_(II)OH → Cu_*B*_(I)OH_2_ **E** → **R**: Fe_a3_(III)OH →Fe_a3_(II)OH_2_	−0.52	−0.55	+0.58	−0.15	+0.65
	0.46	−0.43	+0.58	−0.03	+0.65
	−0.10	−0.80	+0.58	−0.40	+0.65
	−0.10	−0.10	+0.58	+0.30	+0.65
**R**→ **A**: O_2_ binding **A** → **P**_*M*_: O-O cleavage	−0.02*^a^*	−0.02	+0.45	−0.02	**+0.75**
	−0.30*^a^*	−0.30	+0.55	−0.30	**+0.83**
**Sum**	−1.50	−2.2		−0.60	

^a^ The energetics of these reaction steps are taken from the calculations.

In Table 1 the starting point for the reduction process is the **P**_*M*_ state, with Fe_*a*3_(IV)=O-Cu_*B*_(II)OH-TyrO^•^ obtained after the O-O bond cleavage. The first column in Table 1 describes which cofactor is reduced in each step, and the order of the cofactor reductions follows the experimental assignments (Kaila et al., 2010). The second column in Table 1 shows the exergonicity of each reduction step obtained from the experimental reduction potentials (Kaila et al., 2010). The uneven distribution of the free energies over the reduction steps was noted at an early stage (Wikström and Morgan, 1992), and it was realized that the low driving force of two of the reduction steps was not compatible with the experimental observations that proton pumping occurs in all four reduction steps in the A family C*c*Os (Verkhovsky et al., 1999; Bloch et al., 2004). It was also pointed out that the sum of the driving forces based on the experimental reduction potentials would be significantly smaller than the overall exergonicity of the reduction process of 2.2 V, with more than half a volt missing (Kaila et al., 2010), see Table 1. The calculated proton coupled reduction potentials give a different picture, with the corresponding exergonicities shown as the third column in Table 1. The main difference is that the Cu_*B*_(II) potential is significantly higher than the experimental value, resulting in a reasonable energy profile for the entire catalytic cycle with three significantly exergonic reduction steps (>0.4 eV) (Blomberg, 2016; Blomberg, 2019). The calculated Cu_*B*_(II) potential is supported by more reliable CCSD(T) calculations on a smaller model of the copper-complex (Blomberg and Siegbahn, 2015a). The calculations show that the Cu_*B*_(II) potential itself is high during catalytic turnover, with no need for involvement of any structurally excited state (Blomberg, 2020c). There is, however, still one reduction step with a very low exergonicity, Fe_*a*3_(III)OH → Fe_*a*3_(II)OH_2_ (0.1 eV). The fourth column in Table 1 contains estimated barrier heights for the individual reaction steps, and since without gradient, all reaction steps are exergonic, the total barrier for each step is the same as the local barrier. The proton coupled reduction steps are rate limited by proton transfer (including the pump-protons), which based on experimental data, is estimated to occur on the millisecond time-scale. Using transition state theory, this corresponds to a barrier of about 0.58 eV (13.3 kcal/mol). From experiments the O_2_ binding is known to occur in about 10 μs, corresponding to a barrier of about 0.45 eV (10.6 kcal/mol), and the O-O bond cleavage occurs in about 300 μs, corresponding to a barrier of about 0.55 eV (12.6 kcal/mol).

The main problem with a low exergonicity for a reaction step occurs when the electrochemical gradient is present across the membrane. Both the electrogenic chemistry and the proton pumping correspond to charge motion against the gradient across the entire membrane, which means that the exergonicity decreases in reaction steps involving these processes. The maximum electrochemical gradient is some 0.20–0.22 V (Wikström et al., 2018), and in the fifth column in Table 1 the calculated driving forces of the reduction steps are given for the case of a gradient of 0.2 V. Each charge moved across the membrane decreases the exergonicity by 0.2 eV, and assuming that proton pumping occurs in all steps means that the exergonicity of all steps are decreased by 0.4 eV. The low reduction potential of the Fe_*a*3_(III)OH cofactor, results in an endergonic **E** to **R** step (+0.30 eV) when a high gradient is present. Since the reduction steps are rate-limited by proton transfer against the gradient, the barriers are expected to increase, a slightly higher value of 0.65 eV (15 kcal/mol) is chosen here. The local barriers for O_2_ binding and O-O bond cleavage are not expected to be affected by the gradient across the membrane. However, with an endergonic reaction step present, the total barriers increase for the succeeding reaction steps, since the total barrier has to be calculated relative to the preceding point with the lowest energy, i.e., the **E** state in this case. Therefore, the barriers for O_2_ binding and O-O bond cleavage has to be calculated relative to the **E** state, and become 0.75 (0.30 + 0.45) and 0.83 (0.30–0.02 + 0.55) eV, respectively, when the gradient is present (see column six in Table 1). A barrier of 0.83 V (about 19 kcal/mol) corresponds to a very slow reaction, with only a few molecules reduced per minute, which is much slower than observed rates of at least a few hundreds per second in the working oxidase enzyme.

To avoid the strongly endergonic reaction step when the gradient is present over the membrane, and thereby the too high barriers, it was at an early stage suggested that the tyrosine should stay unprotonated until the **R** state is formed, a type of mechanism which has later been further clarified (Blomberg and Siegbahn, 2006; Blomberg, 2016). Table 2 shows the energetics for such a mechanism, where the assignments of the reduction steps are based on the calculated reduction potentials. In this mechanism the **E** state, Cu_*B*_(I)OH_2_-Fe_*a*3_(III)OH-TyrOH, is replaced by the **E**_*H*_ state, Cu_*B*_(I)OH_2_-Fe_*a*3_(II/III)OH_2_-TyrO^•/−^, i.e., the proton was transfered to the center of the BNC rather than to the tyrosine when the **O**_*H*_ state was reduced. The result is that the reduction of Fe_*a*3_(III) with a low potential is mixed with the reduction of TyrO^•^ with a high potential, such that both the reduction steps between state **O**_*H*_ and state **R** have a reasonably large exergonicity, see the second column in Table 2. With this mechanism the **E**_*H*_ to **R** step is estimated to be only slightly endergonic, +0.15 eV, when the full gradient is present, see the fourth column in Table 2. The lower endergonicity of the **E**_*H*_ to **R** step decreases succeeding barriers as compared to the situation in Table 1. Thus, with this mechanism there is no barrier higher than 0.68 eV when a high gradient is present, see the fifth column in Table 2, and the oxygen reduction reaction can proceed with a reasonable rate also with a high gradient. It is noted that this mechanism requires that the **E** state, which has a lower energy than the **E**_*H*_ state, is avoided, i.e., that protonation of the tyrosine is avoided when the **O**_*H*_ state is reduced. In the A-family this is possible due to the presence of the D-channel for proton uptake (see Figure 1), but it also requires a high barrier for proton transfer within the BNC, from the center of the BNC where the D-channel ends, to the tyrosine (where the K-channel ends) (Blomberg and Siegbahn, 2015a; Blomberg, 2016). Experimental information supports the presence of such a barrier for proton transfer within the BNC (Vilhjálmsdóttir et al., 2020). The suggested mechanism implies that only one proton is taken up via the K-channel, in the **E**_*H*_ to **R** step, which is in accordance with the experimental observation that the first reduction of the oxidized state actually does occur in mutants missing the essential lysine in the K-channel (Ganesan and Gennis, 2010). Furthermore, for the B- and the C-family C*c*Os, which have only one proton channel to the BNC, analogous to the K-channel in the A-family, i.e. ending at the redox-active tyrosine, it is not possible to avoid formation of the **E** state. This should be at least part of the explanation for a lower proton pumping stoichiometry in the B-and the C-families.

**TABLE 2:** Driving forces, ∆G (eV), for the individual reduction steps in the catalytic cycle, as obtained from the present calculations, assuming that the tyrosine is left unprotonated until the last reduction step. Note that the first column shows only the cofactor reduced in each particular step, not the full BNC. Rate-limiting barriers, ∆G^#^ (eV), estimated from experimental information are also given for each step. The effects on the driving forces and the barrier heights of the presence of the electrochemical gradient across the membrane are also given.

	No gradient		With gradient: 0.2 V Four protons pumped	
Reduction process	∆**G** (eV) calc	∆**G** ^#^ (eV) est	∆**G** (eV) calc	∆**G** ^#^ (eV) est
**P**_*M*_ →**F**: Cu_*B*_(II)OH → Cu_*B*_(I)OH_2_ **F** →**O**_*H*_: Fe_*a*3_(IV)=O → Fe_*a*3_(III)OH **O**_*H*_ →**E**_*H*_: Fe_*a*3_(III)OHTyrO^•^ → Fe_*a*3_(II/III)OH_2_TyrO^•/−^ **E**_*H*_ →**R**: Fe_*a*3_(II/III)OH_2_TyrO^•/−^ → Fe_*a*3_(II)OH_2_TyrOH	−0.80	+0.58	−0.40	+0.65
	−0.43	+0.58	−0.03	+0.65
	−0.40	+0.58	−0.00	+0.65
	−0.25	+0.58	+0.15	+0.65
**R** →**A**: O_2_ binding **A** →**P**_*M*_: O-O cleavage	−0.02	+0.45	−0.02	**+0.60**
	−0.30	+0.55	−0.30	**+0.68**
**Sum**	−2.2		−0.60	

An alternative solution to the problem with too high barriers, could be that the stoichiometry of the proton pumping decreases when the gradient becomes high. Thus, if the **E** to **R** step in the mechanism shown in Table 1, at high gradient involved only electrogenic chemistry and no proton pumping, this step would be endergonic by only 0.10 eV, which would decrease the succeeding barriers by 0.2 eV compared to the values given in Table 1. Thus, if there exists such mechanism, removing the proton pumping in the **E** to **R** step at high gradient, the reaction could proceed with a reasonable rate also following the mechanism described in Table 1. It has also been suggested that only one proton is pumped during the **O** to **R** steps, and accordingly three protons pumped during the **P** to **O** steps. A detailed scheme for such a proton pumping scenario was constructed based on electrostatic repulsion and the electroneutrality principle (Michel, 1998; Michel, 1999). Another carefully constructed proton pumping scheme implying that only one proton is pumped during the **O** to **R** steps was based on electrostatic calculations (Popovic and Stuchebrukhov, 2004). However, as described in the following section, there are mechanistic aspects on the proton pumping that support the type of mechanism described in Table 2.

In summary, a reaction mechanism is suggested for O_2_ reduction in the A-family C*c*Os (Table 2), in which all reduction steps are exergonic enough to allow proton pumping, also with a high gradient, and without making the overall reaction too slow.

### Coupling of Electron and Proton Transfer

**FIGURE 3:**
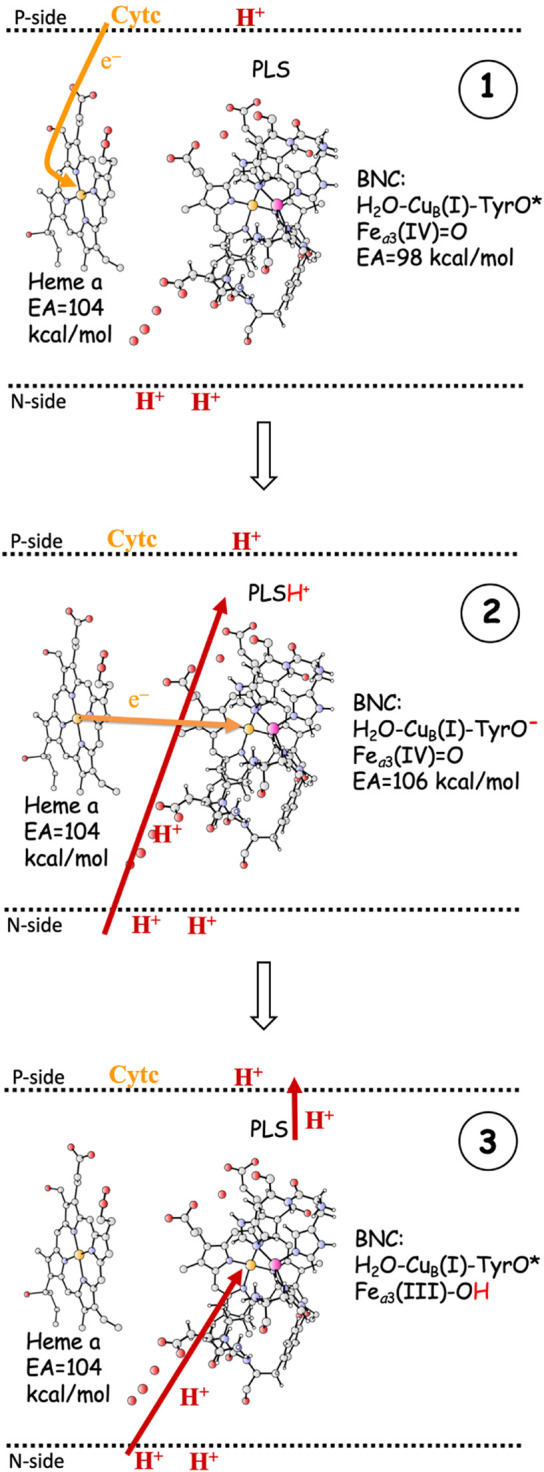
One reduction step, scetching the main steps in a commonly accepted scheme for proton pumping in C*c*O (Wikström et al., 2018). **1**. Electron transfer from soluble cytochrome *c* to the low-spin heme *a*. The EA of the BNC is too low for the electron to move further into the BNC. **2**. The electron in heme *a* triggers proton uptake from the N-side to PLS, which increases the EA of the BNC, such that the electron can move from heme *a* into the BNC. **3**. The electron in the BNC triggers the uptake of the chemical proton from the N-side to the BNC, and the proton in the BNC repels the proton in the PLS, which is ejected to the P-side. - The **F** to **O**_*H*_ reduction is used as an example.

As indicated in the introduction, the mechanisms for proton pumping in C*c*O are not yet fully understood. Many different mechanistic suggestions have been put forward, which are carefully described in a recent review by Wikström and coworkers (Wikström et al., 2018). The basic features of the most commonly accepted view of the proton pumping procedure are summarized in Figure 3, which describes one reduction step in terms of individual electron and proton transfer events (Wikström et al., 2003; Quenneville et al., 2006; Wikström et al., 2018; Blomberg, 2020a). A reduction step is initiated by electron transfer from soluble cytochrome *c* to the low-spin heme *a*, the immediate donor to the BNC, see panel 1 in Figure 3. At this point the electron affinity in the BNC is not large enough to make the electron move further, and the so called proton loading site (PLS) plays an important role (Morgan et al., 1994; Rich et al., 1996). The PLS is a site, not yet exactly known (Sugitani et al., 2008; Wikström et al., 2018), in the vicinity of both the BNC and the low-spin heme. The electron in the low-spin heme increases the proton affinity of the PLS, which triggers the uptake of a proton from the N-side. At the same time, a proton in the PLS will increase the electron affinity of the BNC, which triggers the electron transfer from the low-spin heme to the BNC. It is not clear if this is a two-step procedure or a concerted one, but the result is a proton in the PLS and the electron in the BNC, see panel 2 in Figure 3. The electron in the BNC increases the proton affinity in the BNC, which triggers the uptake of the chemical proton. The proton in the BNC neutralizes the negative charge in the active site and the proton in the PLS is destabilized and expelled to the P-side of the membrane (Michel, 1998; Michel, 1999), see panel 3 in Figure 3.

The most crucial step in the pumping mechanism described in Figure 3 is the one that leads from panel 1 to panel 2, i.e., the initial uptake of the proton to be pumped, and the most crucial property of the active site is the electron affinity (EA) of the BNC, which must be balanced so that the uptake of the pump-proton both is necessary and has a sufficient effect. Initially the EA of the BNC must be lower than the EA of the low-spin heme *a*, otherwise the proton in the PLS would not be needed for the electron transfer to the BNC to occur, and there would be no reliable proton pumping. However, the EA *difference* between the low-spin heme *a* and the BNC must not be too large, because then the effect of the proton in the PLS would not be sufficient to make the electron transfer to the BNC exergonic, which is essential for the state in panel 2 to be stable enough to be inevitably involved in the reduction process. Although the exact position of the PLS is not known, it is generally thought to be somewhere in the vicinity of the A-propionate of the high-spin heme *a*_3_ (Sugitani et al., 2008; Blomberg and Siegbahn, 2012; Wikström et al., 2018). This means that the distance between the proton in the PLS and the BNC cofactor accepting the electron (Fe*a*_3_(III/IV), Cu_*B*_(II), TyrO^•^) most likely is on the order of 10–15 Å. Assuming a dielectric constant of four (Blomberg et al., 1998), the Coulomb interaction energy between the PLS and one of the electron acceptors in the BNC would be on the order of 5–8 kcal/mol. This gives a hint of how much the EA of the BNC cofactors may increase by the proton in the PLS.

**FIGURE 4:**
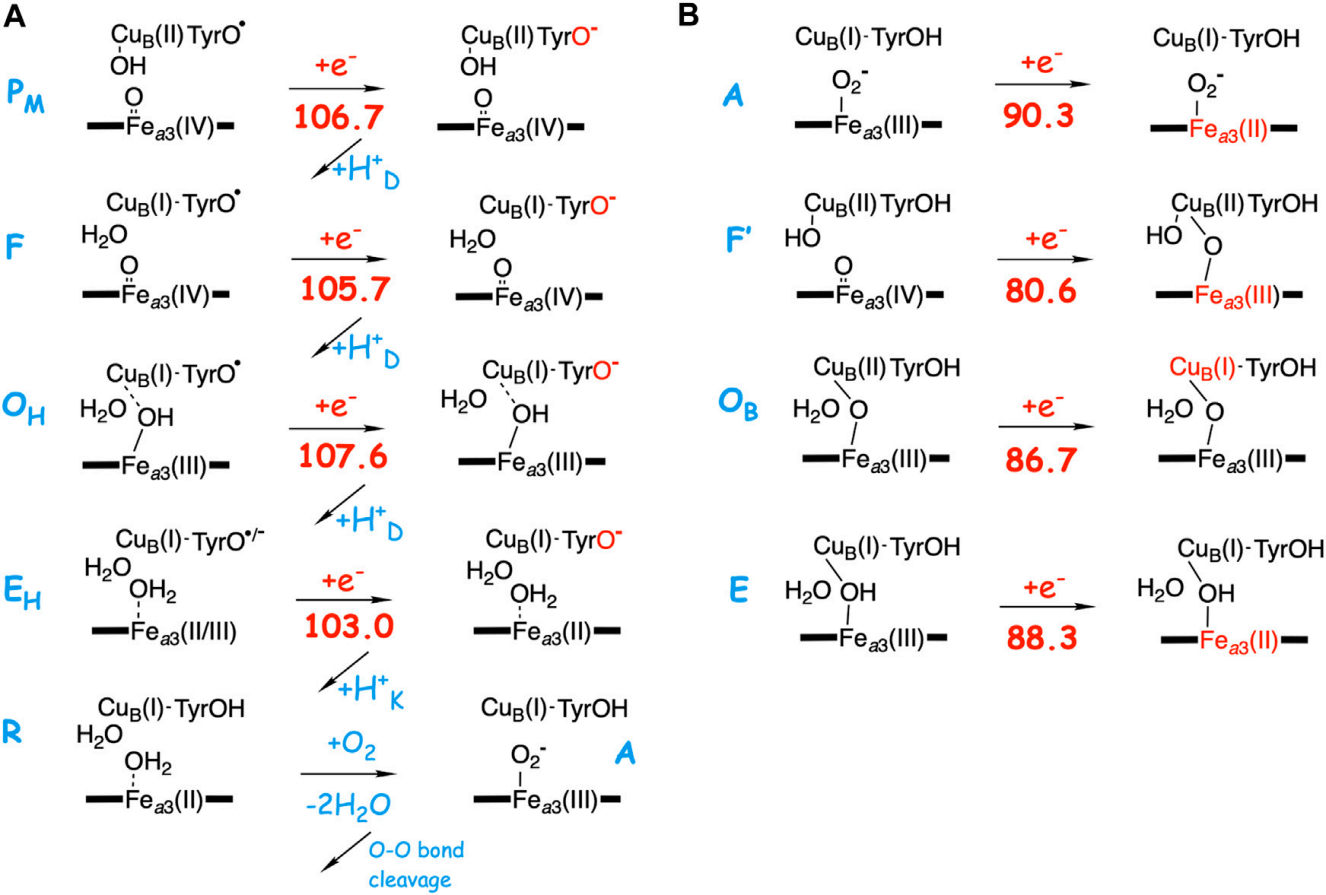
Calculated electron affinities (EA, kcal/mol) of possible intermediates in the catalytic cycle of the A-family C*c*Os. **(A)** The mechanism for O_2_ reduction suggested in Table 2 with calculated EAs for the intermediates that initiate each of the reduction steps. **(B)** Calculated EAs for alternative structures for each reduction step.

As described in the previous paragraph, the affinity for electron uptake (EA) for intermediates that are possible starting structures for the reduction steps is essential for the proton pumping. Using the BNC model described in the computatonal section, the EA of possible structures of the intermediates during the O_2_ reduction process have been calculated by adding just an electron, and the results are summarized in Figure 4. The A-panel in Figure 4 shows the type of reaction mechanism described in Table 2 above. Characteristic of this mechanism is that the tyrosine is unprotonated (radical) in all intermediates except the **R** state, i.e., in all intermediates from which a reduction step is initiated. It is also characteristic that in each case the electron transfer initially yields a tyrosinate. It is therefore not surprising that all these states have a rather similar calculated EA, 103.0–107.6 kcal/mol, where one of the values, 103.0, is slightly lower than the other three, due to the involvement of Fe_*a*3_(III) reduction (see above). As is also shown in Figure 4A, when the chemical proton arrives in the BNC, the electron moves from the tyrosinate to another cofactor in the BNC (except for the **E**_***HR***_ to **R** step). For example, in the **F**_*R*_ state, the new electron is on the tyrosine and the high-spin heme is still in the Fe_*a*3_(IV) state, but when the proton has reached the BNC, forming the **O**_*H*_ state, the high-spin heme turns into Fe*a*_3_(III). This scheme for reduction of the **F** state agrees with experimental observations (Björck and Brzezinski, 2018).

In the B-panel of Figure 4 alternative structures are shown for intermediates that could be the starting point for each of the four reduction steps. Characteristic for these states is that they all have the tyrosine protonated, and also that they all have significantly lower calculated EAs compared to those involved in the suggested mechanism shown in the A-panel in the same figure, 80.6–90.3 kcal/mol, as compared to 103.0–107.6 kcal/mol. The difference in EA at each reduction level ranges between about 15 and 25 kcal/mol. These two sets of calculated EAs cannot be considered as absolute values, but they are comparable to each other, since they are obtained for the same model, with the same charge, in the same environment. Preferably they should also be compared to the corresponding calculated value for the immediate electron donor, the low-spin heme *a*. Unfortunately this cannot be done in a reliable way, since both the model and the environment would be different for a calculated low-spin heme *a* EA, as compared to the BNC, which may lead to incomparable absolute values. However, there is experimental information which can be used to estimate the level of EA values needed in the BNC for the electron transfer to occur at the right stage. In a joint experimental and theoretical study of the fully reduced state of C*c*O, it was shown that the electron present in the low-spin heme was not transferred to the BNC in the **A** state, Fe_*a*3_(III)O_2_^−^Cu_*B*_(I)-TyrOH, but only after the so called **I**_*P*_ state, Fe_*a*3_(III)OOH-Cu_*B*_(I)-TyrO^•^ with a calculated EA of 105.0 kcal/mol (using the present model) had been formed. This shows that the calculated EA of 90.3 kcal/mol for the **A** state (Figure 4B) is too low for electron transfer from the low-spin heme, while a value of 105 kcal/mol works. Furthermore, it is known that after O-O bond cleavage the **P**_*M*_ state is formed, and also that the succeeding reduction step is coupled to proton pumping. In addition, after the O-O bond cleavage there is only one possible type of structure, the **P**_*M*_ state shown in Figure 4A, which means that the calculated EA of this state indicates what is needed for a reduction mechanism that is coupled to proton pumping. Therefore, it can be concluded that only the intermediates with an unprotonated tyrosine and a high EA, similar to that of the **P**_*M*_ state, shown in Figure 4A, can be securely coupled to proton pumping. It should be noted here that even though all intermediates that initiate a reduction step, in Figure 4A are described as having a tyrosyl radical, at least in some cases there may be a partial electron transfer from e.g., Cu_*B*_(I) to form a mixture with tyrosinate. This is the reason for using the notation unprotonated tyrosine rather than tyrosyl radical in the text. The point is that due to the “extra” proton in the center of the BNC, as compared to putting the proton on the tyrosine, also e.g. Cu_*B*_(II) has a high EA, similar to that of the tyrosyl radical. The fact that no tyrosyl radical in the BNC has been observed experimentally may indicate that there is a larger involvement of the tyrosinate electronic structure than the present model calculations suggest. Furthermore, these intermediates with an unprotonated tyrosine are not expected to be very long-lived, compare (Blomberg, 2020c).

**FIGURE 5:**
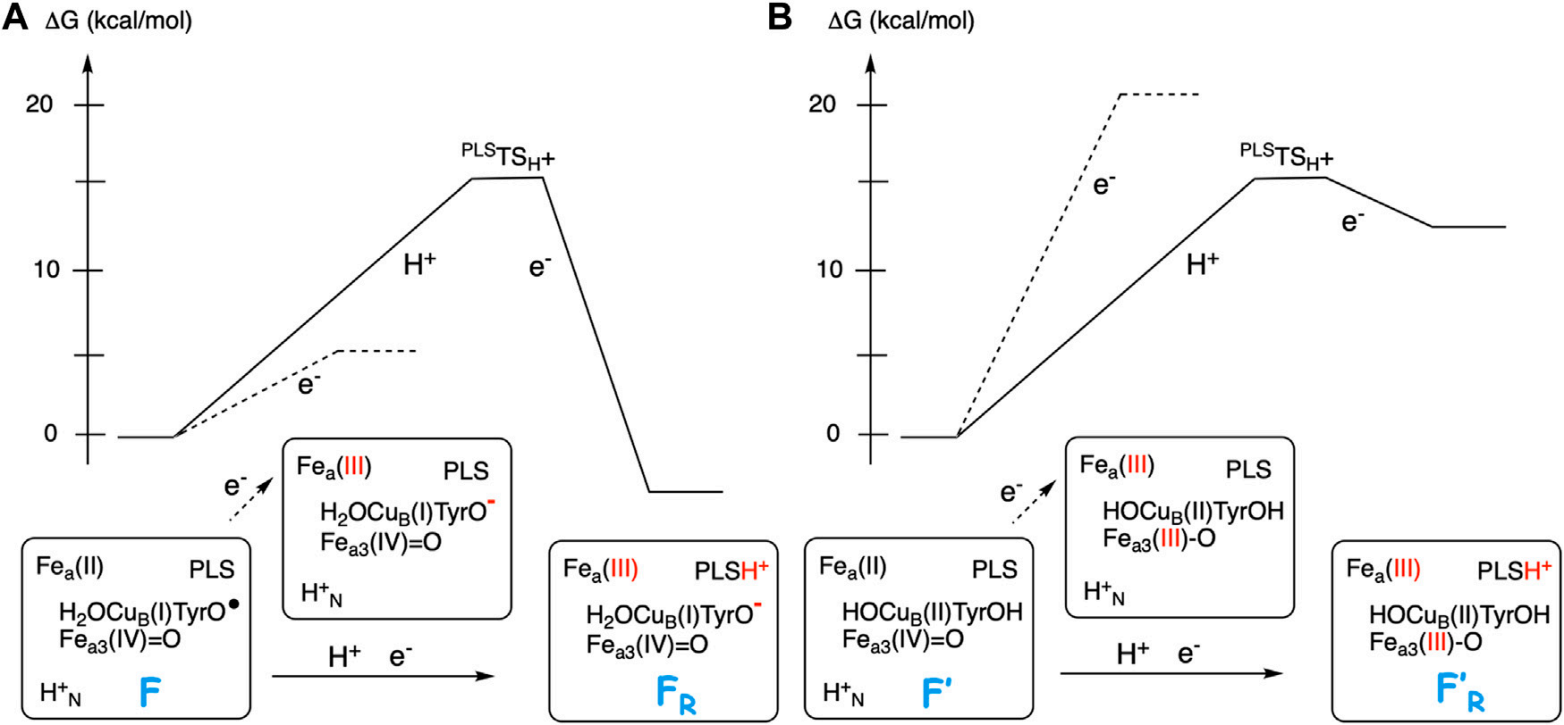
Scetch of energy profiles for coupled electron transfer from heme *a* to the BNC and proton transfer from the N-side to PLS (full curves). The dotted lines correspond to only electron transfer from heme *a* to the BNC. **(A)** For the **F** state, with a high EA, the coupled reaction is exergonic. **(B)** For the **F′** state, with a low EA, the coupled reaction is endergonic.

Figure 5 illustrates the energetics of the coupled electron and proton transfer during the first part of a reduction step, using the **F** (A-panel) and the **F’** (B-panel) intermediates as an example. For the **F** state a pure electron transfer from the low-spin heme *a* to the tyrosyl radical is only slightly endergonic (dotted curve in Figure 5A). Therefore, when the electron transfer is coupled to proton transfer from the N-side of the membrane to the PLS, a rather small change (5–8 kcal/mol) in EA of the BNC makes the coupled process exergonic (full curve in Figure 5A). On the other hand, for the **F’** state with a very low initial EA, a pure electron transfer from the low-spin heme *a* to Fe_*a*3_(IV) is strongly endergonic (dotted curve in Figure 5B). A proton in PLS increases the EA of the BNC, but in this case also the coupled reaction will be endergonic (full curve in Figure 5B), and both the electron and the proton will return back before the next step in the forward reaction, the transfer of the chemical proton to the BNC occurs. In that kind of situation, it is more likely that there is a coupled proton and electron transfer to the BNC, without proton pumping, a mechanism suggested for O_2_ reduction in *c*NOR (Blomberg and Ädelroth, 2017), and also for NO reduction in C*c*O (Blomberg and Ädelroth, 2018). Characteristic of the two latter processes is that there is no tyrosyl radical present in any of the intermediates, in the first case because there is no tyrosine available in the active site of *c*NOR, and in the second case because the active site tyrosine is not involved when NO is reduced in C*c*O.

Finally, to check the accuracy of the calculated EA values reported in Figure 4 the corresponding values are calculated using a fraction of 20% exact exchange in the hybrid B3LYP functional, and the results are reported in Supplementary Figure S1 in the Supplementary Material. The difference in calculated EA between 15 and 20% exact exchange is in all cases less than 2 kcal/mol, supporting the large difference in EA between the two sets of structures discussed above. In summary, it is concluded that the redox-active tyrosine plays an essential role for the proton pumping in C*c*O, which is in accordance with the observation that it is conserved in the active site of all types of C*c*Os (Hemp et al., 2006).

### Gating Barriers

Both the electrogenic chemistry and the proton pumping imply that protons must move against the gradient, i.e. in the direction that is thermodynamically unfavorable. This means that there has to be barriers in the proton pathways, some of which must be flexible. Furthermore, the proton uptake must always occur from the N-side and not from the P-side. This means that there must be a barrier for proton uptake from the P-side, both to the PLS and to the BNC, which is always higher than the barriers for proton uptake from the N-side via the D- and the K-channels. At the same time, the barrier between the PLS and the P-side must be low enough for the pump-protons to be expelled to the P-side. No specific proton pathway has been characterized between the PLS and the P-side, and therefore it is difficult to suggest gating mechanisms in this region. For the protons taken up from the N-side there are mainly two situations that has to be handled by barriers with a varying height. First, when the proton is taken up for the PLS, transfer to the BNC must be avoided. One suggestion is that there is an unfavorable alignment of water molecules toward the BNC at this point of the reaction, the so-called “water-gated” mechanism (Wikström et al., 2003). Another suggestion involves thermodynamic considerations, in which the endergonicity of proton transfer to the BNC before the electron has arrived in the BNC is an important part of the barrier that prevents the proton from going to the BNC rather than to the PLS (Siegbahn and Blomberg, 2007; Blomberg and Siegbahn, 2012).

The most discussed gating situation is probably how to prevent the proton in the PLS to leak back to the N-side when the chemical proton has arrived in the BNC. One suggestion is that the transition state in the D-channel, where all pumped protons are transferred, has a positive character, which means that it is low when there is an uncompensated electron in the vicinity, such as in the low-spin heme *a*, thus allowing the proton to be taken up in the first place. But when the electron is neutralized by the proton in the BNC, the barrier is high, and the proton that is expelled from the PLS cannot return to the N-side, and has to go to the P-side (Siegbahn and Blomberg, 2007; Blomberg and Siegbahn, 2010; Blomberg and Siegbahn, 2012). The positive character of the transition state is achieved by the traveling proton itself, and excludes an initial deprotonation of the glutamic acid at the BNC end of the D-channel. Other suggestions involve charge separation processes for the proton transfer in the D-channel in which the glutamic acid becomes deprotonated, and also motion of the glutamic side chain between different positions (Kaila et al., 2008).

The present computational approach cannot be applied to large enough models to reliably describe the different gating situations in the C*c*Os. Although there have been theoretical studies performed on significantly larger portions of the C*c*O enzyme than the models used in the present study, see e.g., (Goyal et al., 2015; Reidelbach and Imhof, 2020), it has not yet been possible to conclusively quantify the different gating suggestions.

## Conclusion

Cellular energy conservation occurs in the respiratory chain, where one of the members, Cytochrome *c* oxidase (C*c*O), reduces molecular oxygen. The C*c*Os belonging to the A-family are particularly efficient in converting the released free energy in the reduction process to an electrochemical gradient across the membrane. Apart from the electrogenic reduction chemistry, which contributes to the gradient, there is also proton pumping across the entire membrane in every reduction step. The fact that every single electron transfer into the active site for the oxygen reduction is coupled to the motion of two positive charges (protons) across the membrane, requires intricate mechanisms and special energetic preconditions. Quantum chemical calculations suggest a mechanism for the oxygen reduction process that solves some of the pending issues. The main characteristic of the suggested mechanism is that the redox-active tyrosine in the active site, which is conserved in all types of C*c*Os, and which becomes a neutral tyrosyl radical in the O-O bond cleavage step, stays unprotonated in all intermediates from which the reduction steps are initiated, see Table 2 and Figure 4A. This means that the proton goes to the center of the BNC rather than to the tyrosine in each step, and the tyrosine stays as a radical or with some tyrosinate character mixed in. One consequence of such a mechanism is that the free energy released in the oxygen reduction process becomes smoother distributed over the reduction steps, which is needed to avoid too high barriers during the reduction process when the gradient is present. Another consequence is that the affinity for electron transfer to the active site from the immediate donor, the low-spin heme, is adjusted to a value that *both* is small enough to require the uptake of a proton to a pump-loading site, *and* is large enough to secure an exergonic process for the combined uptake of the pump-proton and the electron transfer to the active site, see panel 2 in Figure 3. In this way the tyrosine plays an essential role for a secure proton pumping in all reduction steps.

## Data Availability

The original contributions presented in the study are included in the article/Supplementary Material, further inquiries can be directed to the corresponding author.
