# Supplementary material for: The Redox-Active Tyrosine Is Essential for Proton Pumping in Cytochrome c Oxidase
Source: Front Chem. 2021 Apr 14;9:640155. doi: 10.3389/fchem.2021.640155 (PMC8079940; doi:10.3389/fchem.2021.640155)
Supplement: Supplementary file 1 [file datasheet1.pdf]

Supplementary material for:

The redox-active tyrosine is essential for proton pumping in cytochrome c oxidase.

Margareta R.A. Blomberg*

Department of Organic Chemistry, Arrhenius Laboratory, Stockholm University, SE-106 91, Stockholm, Sweden.

Contact information for corresponding author:
e-mail: margareta.blomberg@su.se, phone: +46-8-16 26 16

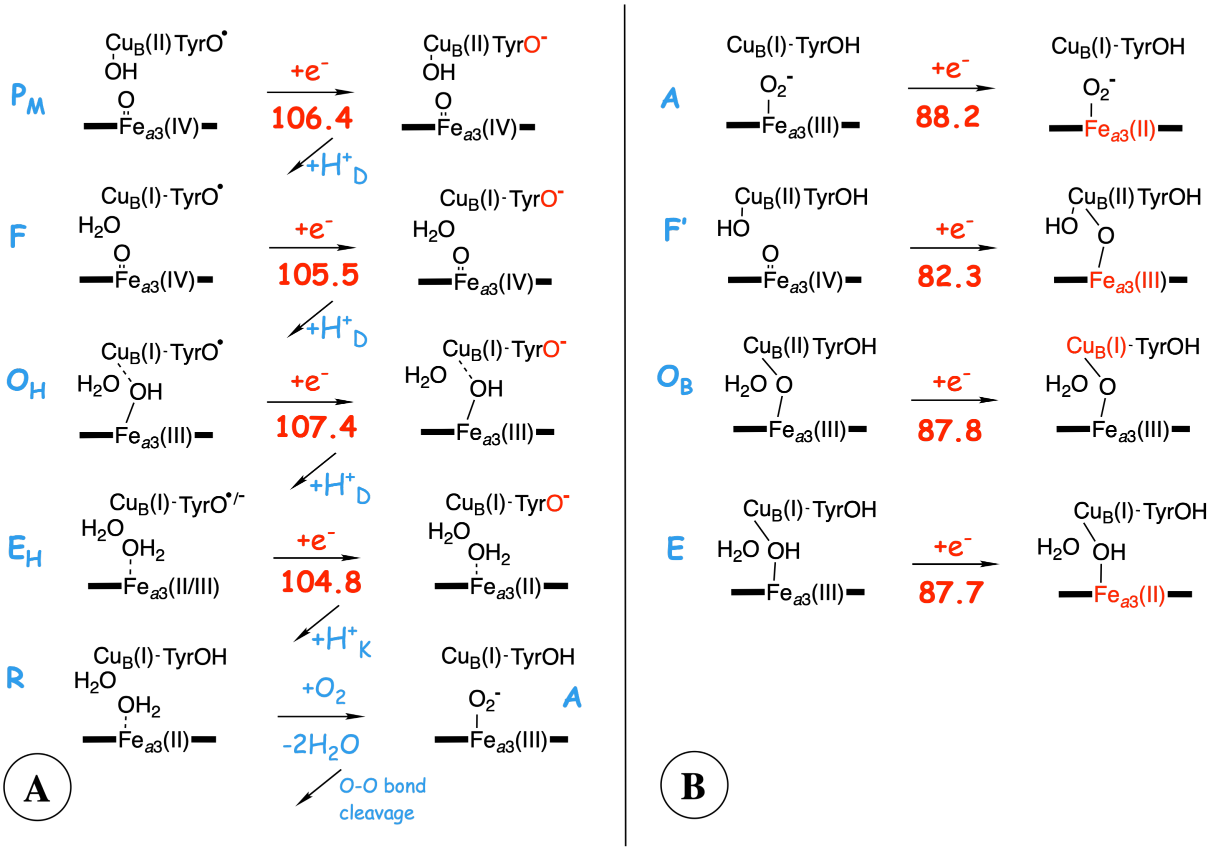

Figure S1. Calculated electron affinities (EA, kcal/mol) of possible intermediates in the catalytic cycle of the A-family CcOs using the B3LYP functional with 20 % exact exchange. **A**. The mechanism for O_2 reduction suggested in Table 2 in the main text with calculated EAs for the intermediates that initiate each of the reduction steps. **B**. Calculated EAs for alternative structures for each reduction step.

Cartesian coordinates for the optimized structures for the C*c*O intermediates:

P$_M$ (quintet, +1)

C1	-0.8191010000	-26.0151710000	24.4889340000
C2	-0.7318120000	-26.8548640000	25.7852190000

C3	-2.0788110000	-27.4070240000	26.1351890000
N4	-2.4551240000	-28.7328740000	25.9290940000
C5	-3.1807940000	-26.7032780000	26.5182210000
C6	-3.7506000000	-28.8216880000	26.1643260000
N7	-4.2516400000	-27.5950300000	26.5238970000
C8	-7.7230360000	-24.0380670000	24.0959640000
C9	-7.5075380000	-24.0594750000	25.6275540000
C10	-7.2358370000	-25.4435980000	26.1515670000
C11	-8.2697290000	-26.3097380000	26.6019500000
C12	-5.9199860000	-25.9205210000	26.1283050000
C13	-7.9872090000	-27.5854280000	27.0282500000
C14	-5.5953190000	-27.1943760000	26.5621970000
C15	-6.6307790000	-28.0957970000	27.0582620000
O16	-6.3464680000	-29.2390940000	27.4998320000
C17	2.9850280000	-33.3740080000	28.0369990000
C18	3.1567820000	-33.5587080000	26.5284940000
O19	2.6195360000	-34.4772380000	25.9160460000
C20	1.4932590000	-33.6571660000	28.3808250000
C21	0.4939480000	-32.7285230000	27.7270950000
N22	-0.4412820000	-32.0272520000	28.4755970000
C23	0.2339540000	-32.3504070000	26.4297640000
C24	-1.1965790000	-31.2644620000	27.6427820000
N25	-0.7979430000	-31.4213220000	26.3896940000
N26	3.8893490000	-32.5854190000	25.9103930000
C27	4.2948730000	-32.6900480000	24.5160120000
C28	4.1344650000	-31.3600650000	23.7425170000
C29	2.7187480000	-30.9032500000	23.6233810000
N30	2.0136890000	-30.8522910000	22.4258420000
C31	1.8027070000	-30.5554160000	24.5792500000
C32	0.7330730000	-30.4894220000	22.6896340000
N33	0.5783060000	-30.3249760000	23.9912760000
C34	-6.9991740000	-37.1460990000	20.0191310000
C35	-5.7205990000	-36.9755780000	20.8581940000
C36	-5.7188550000	-35.7263680000	21.6827560000
N37	-6.7668690000	-35.3861470000	22.5224380000
C38	-4.8006030000	-34.7161670000	21.8303620000
C39	-6.4643070000	-34.2224500000	23.1323680000
N40	-5.2758270000	-33.7897720000	22.7380540000
Cu41	-1.3301400000	-30.0763450000	24.7506890000
Fe42	-4.3062650000	-32.0766410000	23.4678820000
C43	-1.4182790000	-32.9548720000	21.8338180000
C44	-3.4693250000	-34.0749820000	26.0956100000
C45	-7.1517580000	-31.1026360000	25.0966990000
C46	-5.4516440000	-30.5999160000	20.6048700000
N47	-5.9716680000	-31.0538160000	22.9488620000
C48	-1.6530390000	-33.5202370000	23.0848500000
C49	-0.7784630000	-34.4438250000	23.7299390000
C50	-1.3690110000	-34.7890510000	24.9250090000
C51	-2.6066680000	-34.0469060000	25.0035530000
C52	-0.6991980000	-35.5737760000	25.9612330000

O53	-1.0934760000	-35.7344860000	27.1098040000
N54	-5.1435150000	-32.4951290000	25.2521710000
C55	-4.6357830000	-33.3445700000	26.2042030000
C56	-5.5245400000	-33.4010930000	27.3514650000
C57	-6.5765880000	-32.5732460000	27.0698870000
C58	-6.3127350000	-31.9975190000	25.7559570000
C59	-5.2968430000	-34.2705500000	28.5513780000
N60	-2.7639180000	-33.3045770000	23.8696520000
C61	-6.9911230000	-30.6750200000	23.7870220000
C62	-7.9554860000	-29.8533020000	23.0783690000
C63	-7.5454260000	-29.8171670000	21.7644200000
C64	-6.2744740000	-30.5380420000	21.7177030000
C65	-9.1151970000	-29.1399140000	23.7003860000
C66	-8.1705930000	-29.1777460000	20.6139930000
C67	-9.4604440000	-28.8310220000	20.4888290000
N68	-3.5620930000	-31.7993830000	21.6082590000
C69	-4.1699510000	-31.1355950000	20.5803590000
C70	-3.2776370000	-31.0410480000	19.4328650000
C71	-2.1260730000	-31.6668780000	19.8074720000
C72	-2.3152750000	-32.1547470000	21.1520100000
C73	-3.5981710000	-30.3618160000	18.1378650000
C74	-7.8600450000	-32.4490660000	27.8537620000
O75	-8.4208650000	-31.1394710000	27.8369900000
C76	-8.9036060000	-33.4220390000	27.2812670000
H77	0.1811240000	-34.7753110000	23.3517510000
H78	-1.2295580000	-31.8123700000	19.2165680000
H79	-1.2177860000	-26.6722550000	23.7052060000
H80	-0.0365830000	-27.6878430000	25.6313040000
H81	-0.3402900000	-26.2516960000	26.6148170000
H82	-8.5316060000	-24.7348650000	23.8547310000
H83	-6.6798730000	-23.3910270000	25.8982540000
H84	-8.4137300000	-23.6487640000	26.0885540000
H85	3.6244290000	-34.0874440000	28.5670410000
H86	1.2658020000	-34.6851080000	28.0805110000
H87	1.3644410000	-33.6076930000	29.4686300000
H88	3.6962750000	-33.4855420000	24.0701920000
H89	4.7394000000	-30.5880220000	24.2370620000
H90	4.5766170000	-31.4923570000	22.7470070000
H91	-7.8815500000	-37.2044790000	20.6659360000
H92	-5.5998040000	-37.8509180000	21.5122150000
H93	-4.8399860000	-36.9535410000	20.2064320000
H94	-5.9857750000	-34.0241580000	29.3637600000
H95	-4.2747890000	-34.1629300000	28.9328920000
H96	-5.4354550000	-35.3321760000	28.3097230000
H97	-10.0646820000	-29.6717050000	23.5529100000
H98	-9.2130820000	-28.1444240000	23.2511500000
H99	-8.9747130000	-29.0109730000	24.7771760000
H100	-2.7656960000	-30.4527780000	17.4339390000
H101	-3.7956510000	-29.2932090000	18.2900490000
H102	-4.4882710000	-30.7931580000	17.6629510000

H103	-7.6555360000	-32.7445490000	28.8954960000
H104	-8.4843240000	-34.4328990000	27.2376290000
H105	-9.1741080000	-33.1105350000	26.2662140000
H106	-9.8097590000	-33.4560970000	27.8942220000
H107	-0.4837900000	-33.2053030000	21.3419000000
H108	-5.8027970000	-30.1378780000	19.6908070000
H109	-7.5178980000	-28.9764400000	19.7665000000
H110	-10.1978690000	-29.0169240000	21.2613300000
H111	-9.8173720000	-28.3468300000	19.5852410000
H112	-8.0223400000	-30.7704810000	25.6443730000
H113	-3.1710850000	-34.7093850000	26.9203070000
H114	0.2743560000	-35.9974250000	25.6418720000
H115	-7.1059060000	-33.7226450000	23.8404060000
H116	-7.6353170000	-35.9105300000	22.6581540000
H117	-3.8379570000	-34.5918960000	21.3596340000
H118	-0.0589290000	-30.3364430000	21.9729400000
H119	2.3979370000	-31.0252950000	21.5077500000
H120	1.9381550000	-30.4901750000	25.6454870000
H121	4.4024110000	-31.9373460000	26.4936260000
H122	0.7095020000	-32.6940770000	25.5292660000
H123	-0.5801600000	-32.1608810000	29.4667800000
H124	-1.9921870000	-30.6113090000	27.9668670000
H125	-4.3594990000	-29.6985450000	26.0204600000
H126	-3.2980350000	-25.6696220000	26.7979530000
H127	-5.1463730000	-25.2820870000	25.7198300000
H128	-8.7640040000	-28.2600840000	27.3754140000
H129	-9.2959110000	-25.9516050000	26.6031630000
H130	-7.6859710000	-30.4957390000	27.9132210000
H131	3.2712660000	-32.3595090000	28.3144110000
H132	-1.4217070000	-25.2220750000	24.5787790000
H133	0.0891310000	-25.7121940000	24.1996940000
H134	-8.0231020000	-23.1344150000	23.7901120000
N135	-6.5976020000	-24.4875600000	23.3015460000
H136	5.2636450000	-32.9382170000	24.4989930000
H137	-7.1328380000	-36.3013310000	19.3341730000
H138	-6.9519400000	-38.0659640000	19.4266280000
H139	-4.7461400000	-24.9910700000	21.6805950000
C140	-5.5860070000	-25.6409910000	21.3980050000
C141	-6.7881480000	-25.4043790000	22.2880500000
O142	-7.8358950000	-26.0253310000	22.1495880000
C143	-5.1407380000	-27.1217190000	21.4735810000
C144	-3.8242780000	-27.3276960000	20.7164440000
C145	-5.0299080000	-27.6096380000	22.9231950000
H146	-5.7806430000	-23.8946590000	23.2489110000
H147	-5.8654490000	-25.3893240000	20.3683050000
H148	-5.9270080000	-27.7077010000	20.9889530000
H149	-3.0086690000	-26.7638050000	21.1857150000
H150	-3.9059000000	-27.0018580000	19.6712970000
H151	-3.5317540000	-28.3824230000	20.7277370000
H152	-5.9871730000	-27.5348030000	23.4469090000

H153	-4.2801500000	-27.0284400000	23.4773860000
H154	-4.7177190000	-28.6568470000	22.9606510000
H155	-2.6175460000	-29.3088360000	23.0285510000
C156	-11.0219730000	-37.0394970000	21.4210620000
C157	-10.4093520000	-36.6286540000	22.7379150000
O158	-9.1936320000	-36.6823390000	22.9559940000
N159	-11.2737430000	-36.1700520000	23.6690030000
C160	-10.8109980000	-35.7070350000	24.9628930000
H161	-10.3487360000	-37.7314340000	20.9122120000
H162	-11.1579520000	-36.1590460000	20.7816160000
H163	-12.2637960000	-36.1796680000	23.4726300000
H164	-10.0055510000	-34.9778250000	24.8373050000
H165	-11.6400640000	-35.2313420000	25.4908520000
H166	-11.9911560000	-37.5204390000	21.5567630000
H167	-10.4290930000	-36.5298650000	25.5672450000
O168	-1.6788900000	-29.0929490000	23.1490650000
O169	-3.5246910000	-30.7843700000	24.0992850000

$\mathbf{P_R}$ (quartet, 0)

C1	-0.8191110000	-26.0151750000	24.4889560000
C2	-0.7469680000	-26.8636260000	25.7834800000
C3	-2.0878480000	-27.4442580000	26.1286910000
N4	-2.4537410000	-28.7609480000	25.8851510000
C5	-3.1999460000	-26.7716310000	26.5533680000
C6	-3.7638100000	-28.8592550000	26.1287060000
N7	-4.2622240000	-27.6639480000	26.5225640000
C8	-7.7229950000	-24.0380710000	24.0959380000
C9	-7.4670870000	-24.0757690000	25.6144260000
C10	-7.2209780000	-25.4622670000	26.1607150000
C11	-8.2363650000	-26.3123130000	26.6349090000
C12	-5.9301750000	-25.9807830000	26.1443790000
C13	-7.9635510000	-27.6052110000	27.0691680000
C14	-5.6343170000	-27.2650510000	26.5942900000
C15	-6.6432940000	-28.1697160000	27.0718060000
O16	-6.3742170000	-29.3695200000	27.4519390000
C17	2.9850370000	-33.3740040000	28.0369910000
C18	3.1858580000	-33.5788420000	26.5360320000
O19	2.7168280000	-34.5434040000	25.9396290000
C20	1.4927630000	-33.6572530000	28.3745120000
C21	0.4985960000	-32.7225380000	27.7265260000
N22	-0.4414920000	-32.0259820000	28.4741820000
C23	0.2467500000	-32.3401450000	26.4297580000
C24	-1.1935870000	-31.2611680000	27.6355290000
N25	-0.7857210000	-31.4138280000	26.3857380000
N26	3.8785490000	-32.5835880000	25.9057250000
C27	4.2948500000	-32.6900560000	24.5160180000
C28	4.1201060000	-31.3704800000	23.7302950000
C29	2.6982250000	-30.9370060000	23.5990260000
N30	1.9782580000	-30.9602380000	22.4091210000

C31	1.7870140000	-30.5553670000	24.5471100000
C32	0.6948540000	-30.5999550000	22.6730060000
N33	0.5540790000	-30.3717120000	23.9648300000
C34	-7.0641690000	-37.2475660000	20.0469420000
C35	-5.7671940000	-37.0602490000	20.8535400000
C36	-5.7551620000	-35.8045460000	21.6675240000
N37	-6.7795690000	-35.4690740000	22.5385670000
C38	-4.8457590000	-34.7813380000	21.7748930000
C39	-6.4679050000	-34.2934490000	23.1264080000
N40	-5.3007300000	-33.8521730000	22.6870800000
Cu41	-1.3571010000	-30.0430970000	24.7238200000
Fe42	-4.3050360000	-32.1240040000	23.3905530000
C43	-1.4228370000	-33.0485610000	21.7754610000
C44	-3.5004540000	-34.0904010000	26.0487380000
C45	-7.1116340000	-31.0449620000	25.0204520000
C46	-5.4500160000	-30.7038080000	20.5020010000
N47	-5.9513550000	-31.0850960000	22.8617500000
C48	-1.6555940000	-33.5812680000	23.0439220000
C49	-0.7720040000	-34.4667320000	23.7223240000
C50	-1.3690100000	-34.7890740000	24.9249940000
C51	-2.6207000000	-34.0715520000	24.9681430000
C52	-0.6958220000	-35.5397800000	25.9774460000
O53	-1.1001220000	-35.7216300000	27.1214980000
N54	-5.1560450000	-32.5077250000	25.1754630000
C55	-4.6674520000	-33.3590350000	26.1370280000
C56	-5.5658120000	-33.3925090000	27.2782820000
C57	-6.5973580000	-32.5428120000	26.9908710000
C58	-6.3110170000	-31.9752450000	25.6777040000
C59	-5.3611520000	-34.2529410000	28.4889490000
N60	-2.7771380000	-33.3629150000	23.8140180000
C61	-6.9378430000	-30.6388440000	23.7081490000
C62	-7.8732640000	-29.7842980000	22.9978560000
C63	-7.4958070000	-29.8167950000	21.6741120000
C64	-6.2553120000	-30.5899490000	21.6237350000
C65	-8.9577350000	-28.9712720000	23.6345020000
C66	-8.1173970000	-29.1844230000	20.5179120000
C67	-9.3891440000	-28.7701850000	20.4160370000
N68	-3.5671870000	-31.9003010000	21.5211660000
C69	-4.1742940000	-31.2553670000	20.4825840000
C70	-3.2828950000	-31.1877860000	19.3331740000
C71	-2.1331730000	-31.8117070000	19.7168220000
C72	-2.3208120000	-32.2697750000	21.0723930000
C73	-3.5995020000	-30.5231780000	18.0293960000
C74	-7.8751870000	-32.3686330000	27.7841510000
O75	-8.4189310000	-31.0697520000	27.7285840000
C76	-8.9207960000	-33.3770830000	27.2530420000
H77	0.1971480000	-34.7926030000	23.3635540000
H78	-1.2367090000	-31.9688050000	19.1280980000
H79	-1.2154980000	-26.6713220000	23.7033250000
H80	-0.0343980000	-27.6841510000	25.6325420000

H81	-0.3691690000	-26.2583830000	26.6192800000
H82	-8.5438660000	-24.7279780000	23.8744180000
H83	-6.6182730000	-23.4183330000	25.8582310000
H84	-8.3463340000	-23.6238650000	26.0940070000
H85	3.6206970000	-34.0839650000	28.5775720000
H86	1.2658360000	-34.6844600000	28.0701410000
H87	1.3638700000	-33.6140860000	29.4628410000
H88	3.7027810000	-33.4929730000	24.0729870000
H89	4.7100470000	-30.5837610000	24.2209280000
H90	4.5719320000	-31.5062560000	22.7391800000
H91	-7.9280870000	-37.3097190000	20.7176270000
H92	-5.6235520000	-37.9318730000	21.5086120000
H93	-4.9031370000	-37.0352750000	20.1798310000
H94	-6.0294130000	-33.9588050000	29.3031430000
H95	-4.3308220000	-34.1838800000	28.8577610000
H96	-5.5505280000	-35.3123890000	28.2703000000
H97	-9.9390260000	-29.4631160000	23.5793460000
H98	-9.0331120000	-28.0035100000	23.1243880000
H99	-8.7394820000	-28.7719020000	24.6875740000
H100	-2.7707000000	-30.6345420000	17.3231530000
H101	-3.7832870000	-29.4499510000	18.1675610000
H102	-4.4978790000	-30.9479920000	17.5635950000
H103	-7.6524070000	-32.6434100000	28.8311370000
H104	-8.4758370000	-34.3765340000	27.1784880000
H105	-9.2398480000	-33.0610010000	26.2529620000
H106	-9.8097420000	-33.4560700000	27.8942260000
H107	-0.4847690000	-33.3062060000	21.2930720000
H108	-5.8017150000	-30.2565380000	19.5807300000
H109	-7.4762950000	-29.0421980000	19.6492060000
H110	-10.1087940000	-28.8845880000	21.2186570000
H111	-9.7427780000	-28.2861560000	19.5107210000
H112	-7.9501200000	-30.6542640000	25.5782150000
H113	-3.2083720000	-34.7112310000	26.8860680000
H114	0.3010650000	-35.9255030000	25.6767060000
H115	-7.0888590000	-33.7920190000	23.8518070000
H116	-7.6336000000	-36.0031620000	22.7087800000
H117	-3.9001580000	-34.6483840000	21.2725390000
H118	-0.1048520000	-30.4875720000	21.9570310000
H119	2.3525760000	-31.1727180000	21.4958020000
H120	1.9297390000	-30.4370140000	25.6084540000
H121	4.3199020000	-31.8737910000	26.4745390000
H122	0.7241760000	-32.6844560000	25.5297580000
H123	-0.5801600000	-32.1608850000	29.4667780000
H124	-1.9948590000	-30.6087040000	27.9497810000
H125	-4.3725470000	-29.7367730000	25.9934560000
H126	-3.3304950000	-25.7495440000	26.8678820000
H127	-5.1307820000	-25.3725450000	25.7307360000
H128	-8.7652900000	-28.2406560000	27.4382650000
H129	-9.2647690000	-25.9519540000	26.6592600000
H130	-7.6629160000	-30.4014050000	27.7387520000

H131	3.2712640000	-32.3595090000	28.3144190000
H132	-1.4217120000	-25.2220610000	24.5787770000
H133	0.0891380000	-25.7122000000	24.1996970000
H134	-8.0231300000	-23.1344180000	23.7901000000
N135	-6.6237130000	-24.4948390000	23.2708780000
H136	5.2636490000	-32.9382130000	24.4989890000
H137	-7.2230010000	-36.4061510000	19.3631530000
H138	-7.0251090000	-38.1700020000	19.4567010000
H139	-4.7319150000	-25.0266150000	21.7184160000
C140	-5.5860210000	-25.6409800000	21.3980580000
C141	-6.8215520000	-25.3431130000	22.2198120000
O142	-7.9106400000	-25.8481040000	21.9535830000
C143	-5.1974750000	-27.1344050000	21.4757710000
C144	-3.9280740000	-27.4059260000	20.6584700000
C145	-5.0299200000	-27.6096500000	22.9231680000
H146	-5.7030990000	-24.1082120000	23.4176060000
H147	-5.8063850000	-25.3749770000	20.3569010000
H148	-6.0290930000	-27.6910610000	21.0351300000
H149	-3.0707440000	-26.8584980000	21.0712050000
H150	-4.0500640000	-27.1033230000	19.6094060000
H151	-3.6731080000	-28.4706670000	20.6817910000
H152	-5.9404980000	-27.4698600000	23.5124930000
H153	-4.2199580000	-27.0630260000	23.4262680000
H154	-4.7764620000	-28.6738400000	22.9595780000
H155	-2.5809360000	-29.0222660000	23.0222380000
C156	-11.0219660000	-37.0394970000	21.4210660000
C157	-10.4099700000	-36.6713870000	22.7532600000
O158	-9.2065080000	-36.8032490000	22.9985960000
N159	-11.2700360000	-36.1714440000	23.6674860000
C160	-10.8109890000	-35.7070350000	24.9628890000
H161	-10.3540210000	-37.7213060000	20.8926300000
H162	-11.1602560000	-36.1417880000	20.8072910000
H163	-12.2464990000	-36.0816540000	23.4287240000
H164	-10.0073020000	-34.9735830000	24.8475550000
H165	-11.6431590000	-35.2349510000	25.4887330000
H166	-11.9911550000	-37.5204390000	21.5567610000
H167	-10.4290950000	-36.5298650000	25.5672470000
O168	-1.6140350000	-29.0519470000	23.1109750000
O169	-3.4903530000	-30.8432640000	23.9931220000

F (quartet, +1)

C1	-0.8188990000	-26.0151840000	24.4889260000
C2	-0.7531820000	-26.9175050000	25.7407610000
C3	-2.1082020000	-27.4822100000	26.0258370000
N4	-2.5161210000	-28.7459640000	25.5997070000
C5	-3.1891000000	-26.8153930000	26.5227760000
C6	-3.8115820000	-28.8360810000	25.8390660000
N7	-4.2799630000	-27.6701240000	26.3997610000

C8	-7.7228760000	-24.0381160000	24.0959510000
C9	-7.4951290000	-24.0629690000	25.6228770000
C10	-7.2305500000	-25.4595170000	26.1106680000
C11	-8.2838560000	-26.3545070000	26.4430080000
C12	-5.9139640000	-25.9313350000	26.1173970000
C13	-8.0217090000	-27.6563400000	26.7948190000
C14	-5.6146360000	-27.2393020000	26.4576720000
C15	-6.6664840000	-28.1627900000	26.8587890000
O16	-6.3894180000	-29.3281920000	27.2534500000
C17	2.9851550000	-33.3739550000	28.0369400000
C18	3.1650900000	-33.5764470000	26.5345900000
O19	2.6517560000	-34.5174520000	25.9341930000
C20	1.5192180000	-33.6790890000	28.4229970000
C21	0.5056550000	-32.7907070000	27.7523330000
N22	-0.4656460000	-32.1134450000	28.4699250000
C23	0.2359760000	-32.4735760000	26.4406150000
C24	-1.2625280000	-31.4358630000	27.6051480000
N25	-0.8551540000	-31.6276890000	26.3637430000
N26	3.8826460000	-32.5985370000	25.9108070000
C27	4.2946970000	-32.6899570000	24.5164130000
C28	4.0543140000	-31.3603820000	23.7643530000
C29	2.6102630000	-30.9902080000	23.5885830000
N30	1.9478300000	-31.0931840000	22.3715480000
C31	1.6476350000	-30.5410000000	24.4587480000
C32	0.6518220000	-30.7201210000	22.5427020000
N33	0.4344600000	-30.3862250000	23.8056030000
C34	-6.9089040000	-37.4395830000	20.1289580000
C35	-5.6752770000	-37.2140770000	21.0206660000
C36	-5.6855430000	-35.8891530000	21.7169130000
N37	-6.7652330000	-35.4499020000	22.4642330000
C38	-4.7541620000	-34.8856620000	21.8078690000
C39	-6.4639560000	-34.2406460000	22.9779660000
N40	-5.2503110000	-33.8677480000	22.5980680000
Cu41	-1.4550940000	-30.3687590000	24.7794210000
Fe42	-4.2364050000	-32.1513560000	23.2453370000
C43	-1.3864020000	-33.2514690000	21.6800040000
C44	-3.4896130000	-34.0080410000	25.9949470000
C45	-7.1259210000	-31.0597440000	24.7748780000
C46	-5.3071990000	-30.7957020000	20.2996810000
N47	-5.8631070000	-31.1049820000	22.6694090000
C48	-1.6298660000	-33.6895870000	22.9782490000
C49	-0.7667750000	-34.5572240000	23.7106270000
C50	-1.3690250000	-34.7890470000	24.9250200000
C51	-2.6018830000	-34.0409620000	24.9261740000
C52	-0.7319540000	-35.5225180000	26.0193100000
O53	-1.1481820000	-35.6112250000	27.1675400000
N54	-5.1289440000	-32.4488450000	25.0453380000
C55	-4.6609740000	-33.2802730000	26.0356080000
C56	-5.5911350000	-33.3148570000	27.1461450000
C57	-6.6437420000	-32.5087130000	26.8047620000

C58	-6.3248350000	-31.9480970000	25.4935610000
C59	-5.4050950000	-34.1504210000	28.3772140000
N60	-2.7453940000	-33.4000890000	23.7323720000
C61	-6.9069480000	-30.6749530000	23.4596250000
C62	-7.8470550000	-29.8881800000	22.6733840000
C63	-7.3939700000	-29.9317090000	21.3747840000
C64	-6.1368470000	-30.6650980000	21.4021990000
C65	-9.0638220000	-29.1926190000	23.1936720000
C66	-8.0125270000	-29.4402200000	20.1404300000
C67	-8.6209550000	-28.2568590000	19.9705720000
N68	-3.4842050000	-32.0345840000	21.3685300000
C69	-4.0561050000	-31.3949340000	20.3026030000
C70	-3.1645300000	-31.4203000000	19.1544820000
C71	-2.0516590000	-32.0922700000	19.5627610000
C72	-2.2598190000	-32.4852560000	20.9330310000
C73	-3.4448040000	-30.7886410000	17.8270800000
C74	-7.9231590000	-32.3587880000	27.6054710000
O75	-8.5307360000	-31.0793880000	27.4801560000
C76	-8.9586340000	-33.4200520000	27.1990300000
H77	0.1917240000	-34.9302560000	23.3705520000
H78	-1.1643090000	-32.3180690000	18.9839710000
H79	-1.2223350000	-26.6155240000	23.6592390000
H80	-0.0550090000	-27.7434200000	25.5595820000
H81	-0.3803180000	-26.3517930000	26.6033690000
H82	-8.5284420000	-24.7371940000	23.8538040000
H83	-6.6612020000	-23.4026620000	25.8926930000
H84	-8.3956020000	-23.6534630000	26.0962050000
H85	3.6484310000	-34.0733410000	28.5593510000
H86	1.3025360000	-34.7201390000	28.1589910000
H87	1.4110450000	-33.5978200000	29.5110020000
H88	3.7116110000	-33.5008130000	24.0739290000
H89	4.5748500000	-30.5545810000	24.2985130000
H90	4.5418290000	-31.4267690000	22.7832280000
H91	-7.8281530000	-37.4025990000	20.7237680000
H92	-5.6157880000	-38.0220340000	21.7636260000
H93	-4.7595270000	-37.2775230000	20.4227390000
H94	-6.0848920000	-33.8448980000	29.1774790000
H95	-4.3812050000	-34.0764690000	28.7605290000
H96	-5.5935190000	-35.2122990000	28.1735690000
H97	-9.8051770000	-29.0859660000	22.3956510000
H98	-8.8175280000	-28.1762030000	23.5298180000
H99	-9.5256050000	-29.7280370000	24.0301320000
H100	-2.6225280000	-30.9688070000	17.1284730000
H101	-3.5718180000	-29.7030640000	17.9260200000
H102	-4.3630750000	-31.1831210000	17.3744720000
H103	-7.6649430000	-32.5321460000	28.6629120000
H104	-8.4953570000	-34.4122580000	27.1815110000
H105	-9.3322080000	-33.1912270000	26.1949880000
H106	-9.8097510000	-33.4560930000	27.8942200000
H107	-0.4601510000	-33.5692480000	21.2123110000

H108	-5.6380170000	-30.3421730000	19.3730050000
H109	-7.9653540000	-30.1293410000	19.2961060000
H110	-8.6281960000	-27.4955560000	20.7424180000
H111	-9.0781780000	-28.0073720000	19.0162280000
H112	-8.0126260000	-30.7010590000	25.2781640000
H113	-3.2135480000	-34.6024360000	26.8561920000
H114	0.2386430000	-35.9825360000	25.7416860000
H115	-7.1269380000	-33.6640590000	23.6032030000
H116	-7.6482690000	-35.9474600000	22.6125760000
H117	-3.7700030000	-34.8256110000	21.3727530000
H118	-0.0884020000	-30.6735270000	21.7579400000
H119	2.3692480000	-31.3570140000	21.4922030000
H120	1.7348350000	-30.3589210000	25.5182570000
H121	4.3458050000	-31.9082740000	26.4860840000
H122	0.7371130000	-32.8127560000	25.5508020000
H123	-0.5801650000	-32.1608660000	29.4666540000
H124	-2.0977030000	-30.8198810000	27.9032340000
H125	-4.4432500000	-29.6730160000	25.5852570000
H126	-3.2826810000	-25.8340990000	26.9593350000
H127	-5.1208260000	-25.2702060000	25.7888570000
H128	-8.8166580000	-28.3508070000	27.0488010000
H129	-9.3100530000	-25.9975280000	26.4083750000
H130	-7.8081160000	-30.4222930000	27.5741370000
H131	3.2712160000	-32.3595430000	28.3144170000
H132	-1.4218130000	-25.2221620000	24.5787930000
H133	0.0891040000	-25.7121280000	24.1996800000
H134	-8.0232120000	-23.1344050000	23.7901190000
N135	-6.5918550000	-24.4708100000	23.3001460000
H136	5.2636590000	-32.9382820000	24.4987580000
H137	-6.9744310000	-36.6711040000	19.3508070000
H138	-6.8592620000	-38.4192500000	19.6421360000
H139	-4.7274330000	-25.0180890000	21.6835290000
C140	-5.5860080000	-25.6409890000	21.3980450000
C141	-6.7783170000	-25.3813900000	22.2914240000
O142	-7.8352700000	-25.9985100000	22.1709420000
C143	-5.2015960000	-27.1346870000	21.4681800000
C144	-3.9594140000	-27.3801770000	20.6085470000
C145	-5.0299260000	-27.6096270000	22.9231740000
H146	-5.7720740000	-23.8818170000	23.2620330000
H147	-5.8619200000	-25.3784960000	20.3700290000
H148	-6.0385400000	-27.6945690000	21.0399270000
H149	-3.0976410000	-26.8147260000	20.9908830000
H150	-4.1327160000	-27.0560400000	19.5741460000
H151	-3.6712670000	-28.4327530000	20.6070460000
H152	-5.9710450000	-27.5464920000	23.4766160000
H153	-4.2792250000	-27.0029660000	23.4515800000
H154	-4.6897700000	-28.6497000000	22.9507710000
H155	-2.5510860000	-30.0143680000	22.3420450000
C156	-11.0219960000	-37.0395360000	21.4210530000
C157	-10.4146340000	-36.6267590000	22.7396760000

O158	-9.1985140000	-36.6708720000	22.9608080000
N159	-11.2798070000	-36.1712070000	23.6709060000
C160	-10.8109980000	-35.7070310000	24.9628980000
H161	-10.3479130000	-37.7328380000	20.9153210000
H162	-11.1574140000	-36.1604270000	20.7804070000
H163	-12.2713830000	-36.2017710000	23.4848520000
H164	-10.0014270000	-34.9850390000	24.8287980000
H165	-11.6350210000	-35.2236060000	25.4913270000
H166	-11.9911380000	-37.5204050000	21.5567650000
H167	-10.4290920000	-36.5298690000	25.5672450000
O168	-1.9325010000	-29.4396630000	21.8427750000
O169	-3.3490230000	-30.8580250000	23.7656170000
H170	-1.7742300000	-28.7042310000	22.4546640000

$\mathbf{F_R}$ (triplet, 0)

C1	-0.8189080000	-26.0151940000	24.4889470000
C2	-0.7712890000	-26.9253600000	25.7368110000
C3	-2.1230800000	-27.5035060000	26.0251560000
N4	-2.5201200000	-28.7645400000	25.6045280000
C5	-3.2146610000	-26.8545020000	26.5363100000
C6	-3.8281730000	-28.8565250000	25.8526740000
N7	-4.2959340000	-27.7093890000	26.4040030000
C8	-7.7228220000	-24.0381360000	24.0959190000
C9	-7.4605870000	-24.0638100000	25.6119020000
C10	-7.2212800000	-25.4537680000	26.1420360000
C11	-8.2532000000	-26.3162770000	26.5542870000
C12	-5.9337880000	-25.9790190000	26.1203650000
C13	-7.9987040000	-27.6281430000	26.9331280000
C14	-5.6593820000	-27.2872330000	26.5080130000
C15	-6.6821690000	-28.1987740000	26.9326820000
O16	-6.4277860000	-29.4156060000	27.2662240000
C17	2.9851480000	-33.3739510000	28.0369260000
C18	3.1811510000	-33.5880970000	26.5387020000
O19	2.7220730000	-34.5628660000	25.9502770000
C20	1.5208670000	-33.6829760000	28.4228820000
C21	0.5051520000	-32.7984310000	27.7548240000
N22	-0.4737280000	-32.1260490000	28.4679220000
C23	0.2376730000	-32.4908710000	26.4421980000
C24	-1.2745790000	-31.4594760000	27.5938910000
N25	-0.8592130000	-31.6557790000	26.3568820000
N26	3.8793530000	-32.5994450000	25.9085640000
C27	4.2946820000	-32.6899450000	24.5164260000
C28	4.0460990000	-31.3675610000	23.7540110000
C29	2.6006490000	-30.9960690000	23.5918900000
N30	1.9261090000	-31.0867860000	22.3791450000
C31	1.6471680000	-30.5544240000	24.4756090000
C32	0.6337990000	-30.7052610000	22.5678620000
N33	0.4309740000	-30.3894980000	23.8362870000

C34	-6.9820530000	-37.4807820000	20.1585670000
C35	-5.7229640000	-37.2536810000	21.0135480000
C36	-5.7223710000	-35.9362600000	21.7235220000
N37	-6.7788090000	-35.5150720000	22.5152800000
C38	-4.7984240000	-34.9232640000	21.7918680000
C39	-6.4725760000	-34.3016170000	23.0222720000
N40	-5.2776280000	-33.9163090000	22.6044980000
Cu41	-1.4680490000	-30.3247390000	24.7854290000
Fe42	-4.2495970000	-32.1826970000	23.2321000000
C43	-1.3896280000	-33.2575910000	21.6706280000
C44	-3.5223700000	-34.0647200000	25.9677870000
C45	-7.0916750000	-31.0280170000	24.7858240000
C46	-5.3558770000	-30.8764230000	20.2786310000
N47	-5.8711620000	-31.1339800000	22.6611800000
C48	-1.6265520000	-33.6918750000	22.9750580000
C49	-0.7510500000	-34.5320090000	23.7183110000
C50	-1.3690350000	-34.7890620000	24.9250180000
C51	-2.6213080000	-34.0797460000	24.9077380000
C52	-0.7307480000	-35.5190200000	26.0148260000
O53	-1.1874040000	-35.7091600000	27.1369420000
N54	-5.1359200000	-32.4762190000	25.0282190000
C55	-4.6863820000	-33.3273660000	26.0098230000
C56	-5.6224790000	-33.3574100000	27.1165600000
C57	-6.6479290000	-32.5124970000	26.7915340000
C58	-6.3146940000	-31.9481370000	25.4863130000
C59	-5.4570200000	-34.2076840000	28.3406330000
N60	-2.7576910000	-33.4306300000	23.7173690000
C61	-6.8782120000	-30.6548580000	23.4693240000
C62	-7.8043250000	-29.8394780000	22.6963810000
C63	-7.3930890000	-29.9296820000	21.3870750000
C64	-6.1592830000	-30.7054730000	21.3949760000
C65	-8.9608140000	-29.0646420000	23.2414320000
C66	-8.0310660000	-29.4319590000	20.1684270000
C67	-8.6836160000	-28.2684080000	20.0283910000
N68	-3.5145630000	-32.0902940000	21.3455450000
C69	-4.1040270000	-31.4769210000	20.2759670000
C70	-3.2199730000	-31.5094110000	19.1220650000
C71	-2.0927880000	-32.1562270000	19.5326820000
C72	-2.2816670000	-32.5253600000	20.9131260000
C73	-3.5178430000	-30.8997860000	17.7877220000
C74	-7.9224060000	-32.3176350000	27.5945190000
O75	-8.5007710000	-31.0426220000	27.4497550000
C76	-8.9618490000	-33.3891830000	27.1929050000
H77	0.2228810000	-34.8779720000	23.3933890000
H78	-1.2047050000	-32.3738100000	18.9509450000
H79	-1.2246670000	-26.6106660000	23.6570030000
H80	-0.0678770000	-27.7476750000	25.5555440000
H81	-0.4001080000	-26.3607500000	26.6024400000
H82	-8.5398850000	-24.7322080000	23.8783930000
H83	-6.6068190000	-23.4104500000	25.8492280000

H84	-8.3356640000	-23.6068610000	26.0942800000
H85	3.6498930000	-34.0689470000	28.5647720000
H86	1.3103580000	-34.7256850000	28.1598210000
H87	1.4160690000	-33.6032330000	29.5117110000
H88	3.7139740000	-33.5038260000	24.0758650000
H89	4.5785150000	-30.5566060000	24.2699640000
H90	4.5208180000	-31.4500960000	22.7672600000
H91	-7.8797440000	-37.4719390000	20.7865920000
H92	-5.6331920000	-38.0710600000	21.7437500000
H93	-4.8256820000	-37.3009000000	20.3860230000
H94	-6.1316860000	-33.8886830000	29.1401350000
H95	-4.4318870000	-34.1578940000	28.7258590000
H96	-5.6684320000	-35.2649230000	28.1317370000
H97	-9.7763130000	-29.0312730000	22.5100690000
H98	-8.6659890000	-28.0255170000	23.4375360000
H99	-9.3378960000	-29.4807720000	24.1798590000
H100	-2.7014550000	-31.0857450000	17.0827880000
H101	-3.6505530000	-29.8135490000	17.8719860000
H102	-4.4394270000	-31.3051380000	17.3508090000
H103	-7.6595140000	-32.4960400000	28.6532400000
H104	-8.4907200000	-34.3782080000	27.1478040000
H105	-9.3518040000	-33.1419140000	26.1987290000
H106	-9.8097280000	-33.4560780000	27.8942220000
H107	-0.4526970000	-33.5546460000	21.2096540000
H108	-5.6956830000	-30.4349330000	19.3492390000
H109	-7.9581920000	-30.0958690000	19.3053700000
H110	-8.7216770000	-27.5211010000	20.8137400000
H111	-9.1454010000	-28.0123960000	19.0775090000
H112	-7.9457090000	-30.6278230000	25.3114970000
H113	-3.2545200000	-34.6684340000	26.8252980000
H114	0.2860670000	-35.8873710000	25.7626630000
H115	-7.1143480000	-33.7349780000	23.6783850000
H116	-7.6470170000	-36.0244450000	22.6912560000
H117	-3.8281130000	-34.8468250000	21.3266720000
H118	-0.1234570000	-30.6305410000	21.8008230000
H119	2.3340730000	-31.3545740000	21.4951860000
H120	1.7446950000	-30.3769070000	25.5349120000
H121	4.2850230000	-31.8652130000	26.4722920000
H122	0.7418550000	-32.8342900000	25.5562780000
H123	-0.5801690000	-32.1608620000	29.4666400000
H124	-2.1178870000	-30.8476220000	27.8800200000
H125	-4.4569020000	-29.7012580000	25.6283680000
H126	-3.3225560000	-25.8730580000	26.9689640000
H127	-5.1204460000	-25.3623680000	25.7472230000
H128	-8.8134510000	-28.2726350000	27.2541350000
H129	-9.2796280000	-25.9498370000	26.5706180000
H130	-7.7470320000	-30.3696940000	27.4802360000
H131	3.2712170000	-32.3595430000	28.3144230000
H132	-1.4218180000	-25.2221450000	24.5787980000
H133	0.0891160000	-25.7121320000	24.1996780000

H134	-8.0232390000	-23.1344070000	23.7901130000
N135	-6.6160520000	-24.4850440000	23.2750890000
H136	5.2636700000	-32.9382900000	24.4987530000
H137	-7.0865440000	-36.6946370000	19.4022920000
H138	-6.9350420000	-38.4485890000	19.6469080000
H139	-4.7166250000	-25.0513520000	21.7234670000
C140	-5.5860240000	-25.6409760000	21.3980870000
C141	-6.8148060000	-25.3137530000	22.2158050000
O142	-7.9212720000	-25.7837540000	21.9416600000
C143	-5.2475400000	-27.1439970000	21.4732590000
C144	-4.0438480000	-27.4381080000	20.5726240000
C145	-5.0299400000	-27.6096320000	22.9231590000
H146	-5.6885090000	-24.1308710000	23.4553890000
H147	-5.7995930000	-25.3665050000	20.3579920000
H148	-6.1131960000	-27.6803910000	21.0749650000
H149	-3.1555270000	-26.8854220000	20.9095480000
H150	-4.2484170000	-27.1352290000	19.5366060000
H151	-3.7809420000	-28.4973030000	20.5839270000
H152	-5.9290590000	-27.4872250000	23.5340000000
H153	-4.2256260000	-27.0353300000	23.4061340000
H154	-4.7393300000	-28.6636890000	22.9552190000
H155	-2.5629100000	-30.0513650000	22.3183840000
C156	-11.0219960000	-37.0395390000	21.4210610000
C157	-10.4148000000	-36.6713180000	22.7550390000
O158	-9.2112190000	-36.7987210000	23.0028110000
N159	-11.2756090000	-36.1747470000	23.6699500000
C160	-10.8109890000	-35.7070310000	24.9628940000
H161	-10.3523690000	-37.7209510000	20.8942660000
H162	-11.1602380000	-36.1417080000	20.8074700000
H163	-12.2535080000	-36.0902860000	23.4350870000
H164	-10.0045320000	-34.9787670000	24.8398380000
H165	-11.6390530000	-35.2292940000	25.4899290000
H166	-11.9911410000	-37.5204050000	21.5567610000
H167	-10.4290940000	-36.5298690000	25.5672470000
O168	-1.9501190000	-29.4613650000	21.8274800000
O169	-3.3523290000	-30.8896940000	23.7215820000
H170	-1.8893540000	-28.6960330000	22.4202840000

O_H (septet, +1)

C1	-0.8189090000	-26.0151940000	24.4889320000
C2	-0.7441340000	-26.8945710000	25.7557320000
C3	-2.0966170000	-27.4388290000	26.0877400000
N4	-2.4822790000	-28.7472060000	25.8067250000
C5	-3.1842180000	-26.7437320000	26.5280480000
C6	-3.7710020000	-28.8359870000	26.0791550000
N7	-4.2623970000	-27.6232240000	26.5128480000
C8	-7.7228630000	-24.0381220000	24.0959470000
C9	-7.4885750000	-24.0501230000	25.6212370000

C10	-7.2250640000	-25.4396610000	26.1287230000
C11	-8.2791660000	-26.3279530000	26.4716800000
C12	-5.9088740000	-25.9080030000	26.1560990000
C13	-8.0159200000	-27.6225570000	26.8469600000
C14	-5.6043810000	-27.2053450000	26.5351260000
C15	-6.6614820000	-28.1291010000	26.9261300000
O16	-6.3966160000	-29.3000490000	27.3152160000
C17	2.9851550000	-33.3739620000	28.0369420000
C18	3.1588160000	-33.5734750000	26.5325830000
O19	2.6418050000	-34.5108380000	25.9306740000
C20	1.5185120000	-33.6702460000	28.4207150000
C21	0.5210520000	-32.7629320000	27.7544690000
N22	-0.4534960000	-32.0929530000	28.4728870000
C23	0.2771680000	-32.4120900000	26.4466320000
C24	-1.2292200000	-31.3843360000	27.6140060000
N25	-0.8018800000	-31.5513730000	26.3763410000
N26	3.8858070000	-32.6010090000	25.9108920000
C27	4.2946800000	-32.6899640000	24.5164050000
C28	4.0595250000	-31.3600500000	23.7646370000
C29	2.6172390000	-30.9997690000	23.5817300000
N30	1.9153300000	-31.2838750000	22.4173300000
C31	1.6845270000	-30.4244590000	24.4081730000
C32	0.6279820000	-30.8797780000	22.5699540000
N33	0.4529650000	-30.3576640000	23.7763330000
C34	-7.0255540000	-37.7492450000	20.3606400000
C35	-5.7512280000	-37.4662780000	21.1753250000
C36	-5.7657320000	-36.1318820000	21.8533780000
N37	-6.7978640000	-35.7217520000	22.6807040000
C38	-4.8749720000	-35.0872340000	21.8515420000
C39	-6.5093850000	-34.4834210000	23.1372390000
N40	-5.3483950000	-34.0694470000	22.6550390000
Cu41	-1.4009530000	-30.3369220000	24.7775590000
Fe42	-4.2693510000	-32.1557120000	23.1656130000
C43	-1.5111420000	-33.4893650000	21.5459230000
C44	-3.4606840000	-33.8805960000	26.0054510000
C45	-7.1890090000	-31.0484790000	24.7025410000
C46	-5.3730460000	-30.8829210000	20.1893440000
N47	-5.9423480000	-31.0906640000	22.5773370000
C48	-1.6685910000	-33.8152500000	22.9039250000
C49	-0.8053270000	-34.6615640000	23.6696070000
C50	-1.3690170000	-34.7890590000	24.9250090000
C51	-2.5769610000	-33.9978450000	24.9245430000
C52	-0.7264850000	-35.4915350000	26.0360640000
O53	-1.1517720000	-35.5601040000	27.1825790000
N54	-5.1469420000	-32.3497090000	25.0639130000
C55	-4.6512090000	-33.1637380000	26.0550770000
C56	-5.5948370000	-33.2131200000	27.1563010000
C57	-6.6740980000	-32.4437940000	26.7928960000
C58	-6.3710480000	-31.8941400000	25.4726070000
C59	-5.4028040000	-34.0222850000	28.4041190000

N60	-2.7169880000	-33.4202270000	23.6986940000
C61	-6.9883930000	-30.6710890000	23.3716340000
C62	-7.9112210000	-29.8782350000	22.5746170000
C63	-7.4407330000	-29.9108070000	21.2752270000
C64	-6.1961090000	-30.6683330000	21.2986870000
C65	-9.1241200000	-29.1664120000	23.0829430000
C66	-8.0448790000	-29.3738380000	20.0565530000
C67	-8.7135100000	-28.2160910000	19.9461310000
N68	-3.5715420000	-32.2060200000	21.2003480000
C69	-4.1589510000	-31.5756780000	20.1427610000
C70	-3.3213890000	-31.7241610000	18.9577270000
C71	-2.2369320000	-32.4549750000	19.3516640000
C72	-2.3925890000	-32.7517700000	20.7582610000
C73	-3.6164390000	-31.1451700000	17.6097050000
C74	-7.9569160000	-32.3121640000	27.5851940000
O75	-8.5807100000	-31.0422040000	27.4312650000
C76	-8.9714820000	-33.3956070000	27.1861270000
H77	0.1191930000	-35.1084750000	23.3240990000
H78	-1.3962810000	-32.7607960000	18.7404370000
H79	-1.2249990000	-26.6271690000	23.6702670000
H80	-0.0637380000	-27.7352620000	25.5783500000
H81	-0.3445320000	-26.3160970000	26.5981040000
H82	-8.5292090000	-24.7377890000	23.8595180000
H83	-6.6528470000	-23.3871940000	25.8791770000
H84	-8.3856960000	-23.6324060000	26.0939640000
H85	3.6478520000	-34.0746690000	28.5583460000
H86	1.2905480000	-34.7064630000	28.1489620000
H87	1.4100450000	-33.5951880000	29.5092050000
H88	3.7109530000	-33.4997140000	24.0727560000
H89	4.5702050000	-30.5511630000	24.3027760000
H90	4.5546740000	-31.4255430000	22.7868330000
H91	-7.9052180000	-37.7552410000	21.0128690000
H92	-5.6185350000	-38.2612950000	21.9229770000
H93	-4.8699190000	-37.5046420000	20.5251200000
H94	-6.0601430000	-33.6838300000	29.2102970000
H95	-4.3700210000	-33.9607730000	28.7646270000
H96	-5.6172610000	-35.0848620000	28.2307090000
H97	-9.9019130000	-29.1363030000	22.3121450000
H98	-8.8819460000	-28.1239200000	23.3280280000
H99	-9.5370850000	-29.6404320000	23.9789730000
H100	-2.8424400000	-31.4187850000	16.8867260000
H101	-3.6636860000	-30.0496100000	17.6544250000
H102	-4.5804780000	-31.4958500000	17.2202590000
H103	-7.7044730000	-32.4665340000	28.6470090000
H104	-8.4849950000	-34.3763850000	27.1558740000
H105	-9.3647680000	-33.1682210000	26.1896040000
H106	-9.8097910000	-33.4561000000	27.8942470000
H107	-0.6261680000	-33.8837010000	21.0543990000
H108	-5.7034770000	-30.4408130000	19.2558310000
H109	-7.9255120000	-29.9927100000	19.1668150000

H110	-8.8012030000	-27.5172560000	20.7701340000
H111	-9.1433560000	-27.9200020000	18.9928280000
H112	-8.0905080000	-30.7038480000	25.1921710000
H113	-3.1791750000	-34.4486620000	26.8837430000
H114	0.2468120000	-35.9532280000	25.7743090000
H115	-7.1426760000	-33.9189870000	23.8045020000
H116	-7.6596010000	-36.2376630000	22.8744540000
H117	-3.9310610000	-34.9984050000	21.3367900000
H118	-0.1384250000	-30.9275470000	21.8074340000
H119	2.3057440000	-31.6857470000	21.5767690000
H120	1.8061110000	-30.0899540000	25.4271130000
H121	4.3548290000	-31.9163490000	26.4886150000
H122	0.7827190000	-32.7389410000	25.5549390000
H123	-0.5801570000	-32.1608660000	29.4666730000
H124	-2.0621890000	-30.7658580000	27.9124740000
H125	-4.3884630000	-29.7148230000	25.9691120000
H126	-3.2856090000	-25.7250770000	26.8646390000
H127	-5.1192740000	-25.2480280000	25.8181780000
H128	-8.8124410000	-28.3136330000	27.1049390000
H129	-9.3055490000	-25.9731960000	26.4261300000
H130	-7.8661800000	-30.3795390000	27.5408650000
H131	3.2712140000	-32.3595430000	28.3144150000
H132	-1.4218280000	-25.2221490000	24.5787910000
H133	0.0891170000	-25.7121240000	24.1996730000
H134	-8.0232220000	-23.1343920000	23.7901120000
N135	-6.5946560000	-24.4740410000	23.2968160000
H136	5.2636750000	-32.9382860000	24.4987600000
H137	-7.1741600000	-36.9836730000	19.5907420000
H138	-6.9598550000	-38.7252540000	19.8679940000
H139	-4.7320190000	-25.0109920000	21.6830250000
C140	-5.5860060000	-25.6409930000	21.3980540000
C141	-6.7819880000	-25.3876510000	22.2905140000
O142	-7.8335330000	-26.0119610000	22.1683180000
C143	-5.1926340000	-27.1313550000	21.4704130000
C144	-3.9335230000	-27.3865320000	20.6388720000
C145	-5.0299250000	-27.6096290000	22.9231680000
H146	-5.7795160000	-23.8781890000	23.2486530000
H147	-5.8613020000	-25.3830380000	20.3688960000
H148	-6.0230150000	-27.6937500000	21.0340520000
H149	-3.0802690000	-26.8201410000	21.0406650000
H150	-4.0778470000	-27.0679490000	19.5990090000
H151	-3.6541320000	-28.4433520000	20.6393170000
H152	-5.9485460000	-27.4828780000	23.5022620000
H153	-4.2166140000	-27.0765650000	23.4358960000
H154	-4.7940370000	-28.6761250000	22.9144570000
H155	-2.3575930000	-30.1269280000	22.1032300000
C156	-11.0220230000	-37.0394780000	21.4210240000
C157	-10.4273530000	-36.7206420000	22.7747690000
O158	-9.2482770000	-36.9454730000	23.0686230000
N159	-11.2753310000	-36.1601180000	23.6655330000

C160	-10.8110120000	-35.7070310000	24.9628930000
H161	-10.3565140000	-37.7087440000	20.8751850000
H162	-11.1645610000	-36.1228520000	20.8375640000
H163	-12.2361200000	-35.9988140000	23.4008390000
H164	-10.0040580000	-34.9762610000	24.8479900000
H165	-11.6402470000	-35.2333980000	25.4921700000
H166	-11.9911060000	-37.5204580000	21.5567840000
H167	-10.4290510000	-36.5298500000	25.5672420000
O168	-1.8280200000	-29.7679000000	21.3470590000
O169	-3.1377760000	-30.5917890000	23.5961930000
H170	-1.4757030000	-28.9350200000	21.6961750000
H171	-3.6978910000	-29.8156740000	23.7495310000

O$_{HR}$ (sextet, 0)

C1	-0.8189510000	-26.0152230000	24.4889490000
C2	-0.7602850000	-26.9044510000	25.7515770000
C3	-2.1093600000	-27.4696450000	26.0768470000
N4	-2.4808530000	-28.7706530000	25.7820110000
C5	-3.2107200000	-26.7938020000	26.5297430000
C6	-3.7876160000	-28.8625930000	26.0494370000
N7	-4.2805520000	-27.6721730000	26.4840770000
C8	-7.7227830000	-24.0381830000	24.0958330000
C9	-7.4549000000	-24.0543420000	25.6116560000
C10	-7.2164240000	-25.4399110000	26.1521530000
C11	-8.2535050000	-26.3024200000	26.5493890000
C12	-5.9282230000	-25.9563310000	26.1564370000
C13	-7.9985190000	-27.6109730000	26.9289380000
C14	-5.6485910000	-27.2605780000	26.5532700000
C15	-6.6807830000	-28.1763110000	26.9465890000
O16	-6.4369880000	-29.3981930000	27.2692640000
C17	2.9851820000	-33.3739430000	28.0369260000
C18	3.1797600000	-33.5900540000	26.5381450000
O19	2.7217020000	-34.5656470000	25.9505060000
C20	1.5197650000	-33.6747440000	28.4186340000
C21	0.5221380000	-32.7687070000	27.7549990000
N22	-0.4562180000	-32.0986980000	28.4704320000
C23	0.2793990000	-32.4273190000	26.4446960000
C24	-1.2356340000	-31.4008980000	27.6003970000
N25	-0.8034570000	-31.5737190000	26.3657970000
N26	3.8791520000	-32.6018560000	25.9080310000
C27	4.2946250000	-32.6899800000	24.5163880000
C28	4.0444520000	-31.3657650000	23.7592350000
C29	2.5983010000	-31.0198020000	23.5746650000
N30	1.8881770000	-31.3415450000	22.4240070000
C31	1.6690610000	-30.4225850000	24.3904400000
C32	0.6002610000	-30.9325160000	22.5749420000
N33	0.4338070000	-30.3779560000	23.7661020000
C34	-7.0659390000	-37.8080220000	20.4091380000

C35	-5.7926500000	-37.5205690000	21.2250330000
C36	-5.8062230000	-36.1832850000	21.8976290000
N37	-6.8115130000	-35.7853550000	22.7651230000
C38	-4.9358580000	-35.1224330000	21.8550210000
C39	-6.5205340000	-34.5370180000	23.2020650000
N40	-5.3904810000	-34.1077960000	22.6696830000
Cu41	-1.4148390000	-30.2803330000	24.7763810000
Fe42	-4.2783120000	-32.1674650000	23.1634900000
C43	-1.5333980000	-33.5293770000	21.5281090000
C44	-3.4727640000	-33.9004970000	25.9972920000
C45	-7.1725250000	-31.0316940000	24.7029550000
C46	-5.4094010000	-30.9394800000	20.1723820000
N47	-5.9605530000	-31.1257340000	22.5621300000
C48	-1.6829950000	-33.8449520000	22.8922790000
C49	-0.8076790000	-34.6677210000	23.6637040000
C50	-1.3690680000	-34.7890700000	24.9250130000
C51	-2.5880230000	-34.0179330000	24.9153750000
C52	-0.7175270000	-35.4817920000	26.0318130000
O53	-1.1372420000	-35.5788230000	27.1800630000
N54	-5.1555410000	-32.3750800000	25.0491050000
C55	-4.6627510000	-33.1846730000	26.0451160000
C56	-5.6052550000	-33.2202800000	27.1490710000
C57	-6.6746440000	-32.4377440000	26.7882820000
C58	-6.3665580000	-31.8945430000	25.4651740000
C59	-5.4139050000	-34.0185510000	28.4042840000
N60	-2.7372920000	-33.4582790000	23.6830960000
C61	-6.9780460000	-30.6665400000	23.3699650000
C62	-7.8836970000	-29.8429530000	22.5831740000
C63	-7.4440430000	-29.9145450000	21.2758580000
C64	-6.2191830000	-30.7054710000	21.2869640000
C65	-9.0465330000	-29.0656410000	23.1101300000
C66	-8.0535890000	-29.3774600000	20.0604710000
C67	-8.6921260000	-28.2042600000	19.9392620000
N68	-3.6029730000	-32.2608030000	21.1815070000
C69	-4.1959890000	-31.6355500000	20.1251830000
C70	-3.3592050000	-31.7818620000	18.9383260000
C71	-2.2712390000	-32.5102840000	19.3292640000
C72	-2.4216210000	-32.8041270000	20.7385370000
C73	-3.6534400000	-31.1953140000	17.5927720000
C74	-7.9546420000	-32.2682300000	27.5822010000
O75	-8.5462160000	-30.9998990000	27.4188750000
C76	-8.9701800000	-33.3647120000	27.1861100000
H77	0.1235280000	-35.1050910000	23.3223840000
H78	-1.4278190000	-32.8092580000	18.7168690000
H79	-1.2269720000	-26.6230550000	23.6679990000
H80	-0.0698020000	-27.7387890000	25.5736410000
H81	-0.3671950000	-26.3278830000	26.5998420000
H82	-8.5383420000	-24.7343500000	23.8843880000
H83	-6.5988250000	-23.4011990000	25.8390200000
H84	-8.3272120000	-23.5922430000	26.0945010000

H85	3.6487410000	-34.0696640000	28.5658130000
H86	1.2966070000	-34.7121660000	28.1456470000
H87	1.4132910000	-33.6025730000	29.5079060000
H88	3.7138000000	-33.5037000000	24.0754260000
H89	4.5468320000	-30.5485250000	24.2939340000
H90	4.5415340000	-31.4301590000	22.7818780000
H91	-7.9454530000	-37.8167270000	21.0621590000
H92	-5.6593480000	-38.3152730000	21.9738290000
H93	-4.9112940000	-37.5588680000	20.5747280000
H94	-6.0728270000	-33.6684300000	29.2045540000
H95	-4.3808810000	-33.9517670000	28.7652890000
H96	-5.6275730000	-35.0838660000	28.2429920000
H97	-9.8502410000	-29.0267090000	22.3660140000
H98	-8.7506160000	-28.0286290000	23.3154680000
H99	-9.4403380000	-29.4870320000	24.0391450000
H100	-2.8789260000	-31.4657630000	16.8679470000
H101	-3.6992560000	-30.0997470000	17.6442200000
H102	-4.6183850000	-31.5408630000	17.2004620000
H103	-7.6942430000	-32.4316670000	28.6443290000
H104	-8.4754860000	-34.3421130000	27.1363450000
H105	-9.3740160000	-33.1282650000	26.1950700000
H106	-9.8097210000	-33.4560590000	27.8942300000
H107	-0.6441580000	-33.9175990000	21.0379980000
H108	-5.7392030000	-30.4956910000	19.2395250000
H109	-7.9647400000	-30.0151100000	19.1802860000
H110	-8.7364960000	-27.4773600000	20.7424700000
H111	-9.1297990000	-27.9165800000	18.9866290000
H112	-8.0437810000	-30.6465030000	25.2147200000
H113	-3.1873640000	-34.4612850000	26.8795110000
H114	0.2663350000	-35.9218900000	25.7632750000
H115	-7.1316440000	-33.9799390000	23.8956030000
H116	-7.6567460000	-36.3109510000	22.9902520000
H117	-4.0142270000	-35.0205610000	21.3037170000
H118	-0.1708660000	-30.9920430000	21.8173540000
H119	2.2695070000	-31.7723830000	21.5943850000
H120	1.7938830000	-30.0555820000	25.3985120000
H121	4.2727390000	-31.8589190000	26.4693740000
H122	0.7892000000	-32.7579960000	25.5554290000
H123	-0.5801560000	-32.1608770000	29.4666920000
H124	-2.0717570000	-30.7793350000	27.8870010000
H125	-4.4067300000	-29.7416360000	25.9535580000
H126	-3.3255560000	-25.7799720000	26.8746660000
H127	-5.1178220000	-25.3324410000	25.7904990000
H128	-8.8132480000	-28.2631660000	27.2349070000
H129	-9.2813330000	-25.9383050000	26.5503600000
H130	-7.7921970000	-30.3296240000	27.4635980000
H131	3.2711930000	-32.3595280000	28.3144170000
H132	-1.4218410000	-25.2221410000	24.5787830000
H133	0.0891240000	-25.7121350000	24.1997220000
H134	-8.0232440000	-23.1344110000	23.7901020000

N135	-6.6171290000	-24.4849360000	23.2723660000
H136	5.2636680000	-32.9382740000	24.4987390000
H137	-7.2157830000	-37.0408060000	19.6408690000
H138	-6.9982930000	-38.7835920000	19.9142680000
H139	-4.7210210000	-25.0432220000	21.7187960000
C140	-5.5860390000	-25.6409650000	21.3981650000
C141	-6.8138280000	-25.3302190000	22.2245220000
O142	-7.9093540000	-25.8329790000	21.9709550000
C143	-5.2404360000	-27.1415620000	21.4752920000
C144	-4.0224780000	-27.4513700000	20.5988460000
C145	-5.0299070000	-27.6096310000	22.9231700000
H146	-5.6999970000	-24.0898720000	23.4185700000
H147	-5.8066140000	-25.3711810000	20.3578660000
H148	-6.1053110000	-27.6785640000	21.0737880000
H149	-3.1386190000	-26.8991850000	20.9507330000
H150	-4.2006080000	-27.1559370000	19.5564540000
H151	-3.7683150000	-28.5145680000	20.6161860000
H152	-5.8956120000	-27.4144630000	23.5613160000
H153	-4.1594720000	-27.1213630000	23.3826560000
H154	-4.8577800000	-28.6882860000	22.9259400000
H155	-2.3780190000	-30.1676250000	22.0619960000
C156	-11.0220260000	-37.0394360000	21.4210240000
C157	-10.4290520000	-36.7624700000	22.7868100000
O158	-9.2776140000	-37.0770340000	23.1017440000
N159	-11.2649340000	-36.1563910000	23.6603810000
C160	-10.8110190000	-35.7070240000	24.9628930000
H161	-10.3605580000	-37.6977040000	20.8568650000
H162	-11.1657200000	-36.1078680000	20.8627590000
H163	-12.1820020000	-35.8789860000	23.3431030000
H164	-10.0094080000	-34.9664690000	24.8675100000
H165	-11.6478750000	-35.2440220000	25.4894100000
H166	-11.9910830000	-37.5204850000	21.5567910000
H167	-10.4290240000	-36.5298390000	25.5672340000
O168	-1.8334610000	-29.7819370000	21.3281120000
O169	-3.1519430000	-30.6177250000	23.5525280000
H170	-1.5296650000	-28.9434600000	21.7074030000
H171	-3.6986370000	-29.8434280000	23.7601890000

E_H (sextet, +1)

C1	-0.8189520000	-26.0152660000	24.4889180000
C2	-0.7654150000	-26.8961850000	25.7554660000
C3	-2.1214300000	-27.4556310000	26.0519310000
N4	-2.5153660000	-28.7402150000	25.6895310000
C5	-3.2095080000	-26.7856730000	26.5315020000
C6	-3.8125840000	-28.8363490000	25.9511600000
N7	-4.2903880000	-27.6534630000	26.4516790000
C8	-7.7228940000	-24.0381810000	24.0959360000
C9	-7.4894210000	-24.0476300000	25.6202410000

C10	-7.2363130000	-25.4358520000	26.1385250000
C11	-8.2847950000	-26.3092210000	26.5160200000
C12	-5.9279480000	-25.9253030000	26.1380940000
C13	-8.0264450000	-27.6096060000	26.8906250000
C14	-5.6358080000	-27.2259290000	26.5144650000
C15	-6.6828400000	-28.1394710000	26.9284760000
O16	-6.4176960000	-29.3219970000	27.2987770000
C17	2.9851480000	-33.3739430000	28.0369260000
C18	3.1608790000	-33.5724490000	26.5328480000
O19	2.6499600000	-34.5124630000	25.9301610000
C20	1.5169540000	-33.6573370000	28.4170490000
C21	0.5318460000	-32.7351880000	27.7536950000
N22	-0.4574070000	-32.0899730000	28.4732340000
C23	0.3004460000	-32.3596730000	26.4502490000
C24	-1.2327080000	-31.3744110000	27.6212980000
N25	-0.7892220000	-31.5127390000	26.3858990000
N26	3.8851130000	-32.5976180000	25.9101490000
C27	4.2945880000	-32.6899570000	24.5163900000
C28	4.0384830000	-31.3729590000	23.7503850000
C29	2.5871230000	-31.0591110000	23.5702000000
N30	1.8235430000	-31.6151140000	22.5526140000
C31	1.6940440000	-30.3165150000	24.3001210000
C32	0.5389660000	-31.1973290000	22.6951350000
N33	0.4249630000	-30.4098870000	23.7518500000
C34	-7.1021600000	-37.9929770000	20.5438790000
C35	-5.8307330000	-37.6819800000	21.3532330000
C36	-5.8580000000	-36.3379670000	22.0112460000
N37	-6.8475160000	-35.9503360000	22.9010980000
C38	-5.0142040000	-35.2596660000	21.9263310000
C39	-6.5832830000	-34.6897890000	23.3084830000
N40	-5.4755700000	-34.2420580000	22.7376940000
Cu41	-1.3311710000	-30.2206610000	24.8472100000
Fe42	-4.4434080000	-32.3868480000	23.1026330000
C43	-1.6466120000	-33.6709630000	21.4863760000
C44	-3.4125580000	-33.7842220000	26.0337520000
C45	-7.1988410000	-31.0476890000	24.6956290000
C46	-5.4527420000	-30.9435370000	20.1597690000
N47	-5.9952960000	-31.1191900000	22.5505160000
C48	-1.7556100000	-33.9292760000	22.8651580000
C49	-0.8693210000	-34.7529750000	23.6381090000
C50	-1.3690200000	-34.7890660000	24.9250180000
C51	-2.5624390000	-33.9638180000	24.9293370000
C52	-0.7031400000	-35.4604570000	26.0396370000
O53	-1.0828780000	-35.4624860000	27.2045400000
N54	-5.1196580000	-32.2857060000	25.0816610000
C55	-4.6102440000	-33.0737920000	26.0850120000
C56	-5.5593060000	-33.1384580000	27.1870440000
C57	-6.6625740000	-32.4146920000	26.8061160000
C58	-6.3619750000	-31.8684100000	25.4774530000
C59	-5.3444670000	-33.9208910000	28.4481600000

N60	-2.7516750000	-33.4563600000	23.6827600000
C61	-7.0191420000	-30.6795980000	23.3551120000
C62	-7.9436860000	-29.8675460000	22.5702720000
C63	-7.4886890000	-29.9053000000	21.2664720000
C64	-6.2563640000	-30.6927010000	21.2787570000
C65	-9.1371000000	-29.1352420000	23.0960060000
C66	-8.0955680000	-29.3591730000	20.0534290000
C67	-8.7673250000	-28.2025570000	19.9467510000
N68	-3.6778430000	-32.3337360000	21.1439860000
C69	-4.2606290000	-31.6839890000	20.0959840000
C70	-3.4382800000	-31.8525790000	18.8998780000
C71	-2.3674700000	-32.6248020000	19.2839850000
C72	-2.5269150000	-32.9251710000	20.6922130000
C73	-3.7451250000	-31.3022770000	17.5393390000
C74	-7.9533420000	-32.2989540000	27.5935270000
O75	-8.5871120000	-31.0374280000	27.4306790000
C76	-8.9576820000	-33.3958090000	27.1984690000
H77	0.0215920000	-35.2516940000	23.2744850000
H78	-1.5525520000	-32.9652980000	18.6554470000
H79	-1.2210320000	-26.6269230000	23.6678890000
H80	-0.0691960000	-27.7278790000	25.5955730000
H81	-0.3954870000	-26.3160380000	26.6098310000
H82	-8.5299530000	-24.7371440000	23.8616580000
H83	-6.6504370000	-23.3868050000	25.8760660000
H84	-8.3837290000	-23.6180880000	26.0879080000
H85	3.6436500000	-34.0772630000	28.5597820000
H86	1.2739970000	-34.6889230000	28.1411210000
H87	1.4078770000	-33.5846980000	29.5057180000
H88	3.7180580000	-33.5107960000	24.0825290000
H89	4.5198710000	-30.5425800000	24.2811130000
H90	4.5375410000	-31.4367690000	22.7739680000
H91	-7.9788500000	-38.0082320000	21.1989040000
H92	-5.6856970000	-38.4636540000	22.1121220000
H93	-4.9501100000	-37.7170720000	20.7018890000
H94	-6.0201910000	-33.5963290000	29.2448670000
H95	-4.3170200000	-33.8121570000	28.8135530000
H96	-5.5144230000	-34.9940070000	28.2907280000
H97	-9.9299400000	-29.0995600000	22.3405490000
H98	-8.8780550000	-28.0944270000	23.3314900000
H99	-9.5379520000	-29.5966590000	24.0039920000
H100	-2.9376300000	-31.5194570000	16.8337820000
H101	-3.8901540000	-30.2147640000	17.5635890000
H102	-4.6681950000	-31.7364770000	17.1351320000
H103	-7.7012600000	-32.4476010000	28.6567960000
H104	-8.4635920000	-34.3736950000	27.1871970000
H105	-9.3398240000	-33.1839140000	26.1937850000
H106	-9.8097920000	-33.4561000000	27.8942510000
H107	-0.7912020000	-34.1117440000	20.9800260000
H108	-5.7858960000	-30.5041330000	19.2249760000
H109	-7.9786710000	-29.9737380000	19.1600060000

H110	-8.8597350000	-27.5062520000	20.7720570000
H111	-9.1994260000	-27.9048900000	18.9948520000
H112	-8.0984720000	-30.7048840000	25.1911510000
H113	-3.1104370000	-34.3195460000	26.9261250000
H114	0.2426040000	-35.9682350000	25.7623560000
H115	-7.1891250000	-34.1335640000	24.0084400000
H116	-7.6927400000	-36.4805640000	23.1276320000
H117	-4.1089570000	-35.1438450000	21.3510820000
H118	-0.2602040000	-31.4721810000	22.0277630000
H119	2.1673120000	-32.2140270000	21.8154180000
H120	1.8708610000	-29.7485220000	25.2010820000
H121	4.3466530000	-31.9065490000	26.4859240000
H122	0.8188000000	-32.6611860000	25.5566140000
H123	-0.5801600000	-32.1608700000	29.4666670000
H124	-2.0788750000	-30.7750230000	27.9224150000
H125	-4.4354750000	-29.7054960000	25.7948190000
H126	-3.3111780000	-25.7893350000	26.9298710000
H127	-5.1320710000	-25.2812540000	25.7814690000
H128	-8.8278080000	-28.2837200000	27.1775150000
H129	-9.3092490000	-25.9450150000	26.5006560000
H130	-7.8728710000	-30.3670960000	27.5229340000
H131	3.2712150000	-32.3595430000	28.3144210000
H132	-1.4218420000	-25.2221090000	24.5788000000
H133	0.0891600000	-25.7121050000	24.1996750000
H134	-8.0232120000	-23.1343480000	23.7900910000
N135	-6.5961650000	-24.4748940000	23.2955130000
H136	5.2637250000	-32.9382820000	24.4987580000
H137	-7.2637380000	-37.2354090000	19.7684750000
H138	-7.0196500000	-38.9710690000	20.0578520000
H139	-4.7131970000	-25.0455440000	21.7005330000
C140	-5.5859970000	-25.6409850000	21.3980940000
C141	-6.7915650000	-25.3573150000	22.2694240000
O142	-7.8584280000	-25.9436450000	22.1025960000
C143	-5.2655650000	-27.1489470000	21.4759410000
C144	-4.0860200000	-27.5172210000	20.5719180000
C145	-5.0298440000	-27.6096210000	22.9231820000
H146	-5.7528940000	-23.9179210000	23.2965580000
H147	-5.8359100000	-25.3701690000	20.3658430000
H148	-6.1538780000	-27.6682780000	21.1069220000
H149	-3.1608510000	-27.0307240000	20.9174460000
H150	-4.2614320000	-27.2038290000	19.5361040000
H151	-3.9409470000	-28.6031680000	20.5744090000
H152	-5.8563710000	-27.3341050000	23.5832270000
H153	-4.1042600000	-27.1888200000	23.3410800000
H154	-4.9707890000	-28.7022400000	22.9433650000
H155	-2.5947600000	-30.3758850000	22.4180910000
C156	-11.0220150000	-37.0394900000	21.4210340000
C157	-10.4392750000	-36.7933120000	22.7979180000
O158	-9.3150910000	-37.1737330000	23.1424050000
N159	-11.2583230000	-36.1412160000	23.6541690000

C160	-10.8110020000	-35.7070310000	24.9628930000
H161	-10.3640260000	-37.6915930000	20.8471660000
H162	-11.1680350000	-36.0983160000	20.8799130000
H163	-12.1557990000	-35.8207890000	23.3197230000
H164	-10.0109530000	-34.9625660000	24.8765220000
H165	-11.6511580000	-35.2493670000	25.4893700000
H166	-11.9911170000	-37.5204510000	21.5567780000
H167	-10.4290590000	-36.5298540000	25.5672440000
O168	-1.7044390000	-29.9136600000	20.9596690000
O169	-3.0069970000	-30.5210420000	23.3094020000
H170	-1.8288550000	-28.9828300000	20.7217270000
H171	-3.5657500000	-29.7404730000	23.4573760000
H172	-2.0314610000	-30.4287850000	20.1984020000

$\mathbf{E_{HR}}$ (quintet, 0)

C1	-0.8189520000	-26.0152660000	24.4889180000
C2	-0.7734470000	-26.8896060000	25.7620180000
C3	-2.1254480000	-27.4589040000	26.0686780000
N4	-2.5071180000	-28.7433400000	25.7166180000
C5	-3.2232380000	-26.7996500000	26.5527110000
C6	-3.8144490000	-28.8423160000	25.9797690000
N7	-4.2967180000	-27.6691990000	26.4665640000
C8	-7.7228940000	-24.0381810000	24.0959360000
C9	-7.4623850000	-24.0422690000	25.6119140000
C10	-7.2237210000	-25.4234180000	26.1613410000
C11	-8.2601710000	-26.2864590000	26.5615790000
C12	-5.9357200000	-25.9423450000	26.1593690000
C13	-8.0077990000	-27.5960750000	26.9422930000
C14	-5.6607730000	-27.2506140000	26.5512240000
C15	-6.6894180000	-28.1662640000	26.9568540000
O16	-6.4424840000	-29.3824010000	27.2860390000
C17	2.9851480000	-33.3739430000	28.0369260000
C18	3.1918860000	-33.5924190000	26.5408380000
O19	2.7551600000	-34.5792120000	25.9560780000
C20	1.5172400000	-33.6636580000	28.4108100000
C21	0.5344830000	-32.7382090000	27.7534160000
N22	-0.4544320000	-32.0897980000	28.4722270000
C23	0.3065040000	-32.3663710000	26.4491310000
C24	-1.2275700000	-31.3761060000	27.6127090000
N25	-0.7811050000	-31.5197180000	26.3799500000
N26	3.8775340000	-32.5966300000	25.9069580000
C27	4.2945880000	-32.6899570000	24.5163900000
C28	4.0211850000	-31.3830150000	23.7400090000
C29	2.5662610000	-31.0769900000	23.5699020000
N30	1.7812620000	-31.6767750000	22.5932350000
C31	1.6898020000	-30.2942660000	24.2783600000
C32	0.5005810000	-31.2431810000	22.7406670000
N33	0.4115120000	-30.4063400000	23.7595460000

C34	-7.0755910000	-38.1028790000	20.6238460000
C35	-5.8205030000	-37.7761190000	21.4529880000
C36	-5.8559980000	-36.4163250000	22.0768640000
N37	-6.8503280000	-36.0138850000	22.9556330000
C38	-5.0203890000	-35.3329540000	21.9664000000
C39	-6.5918640000	-34.7372740000	23.3281730000
N40	-5.4889260000	-34.2983970000	22.7482870000
Cu41	-1.3437190000	-30.1914060000	24.8556920000
Fe42	-4.4417770000	-32.3917080000	23.0893570000
C43	-1.6498240000	-33.6829570000	21.4747100000
C44	-3.4213780000	-33.7905040000	26.0268100000
C45	-7.1949400000	-31.0448530000	24.6868040000
C46	-5.4783690000	-30.9847280000	20.1379410000
N47	-6.0192560000	-31.1498740000	22.5280000000
C48	-1.7558790000	-33.9429440000	22.8584860000
C49	-0.8667090000	-34.7569390000	23.6338830000
C50	-1.3690200000	-34.7890660000	24.9250180000
C51	-2.5660320000	-33.9716680000	24.9228000000
C52	-0.6990140000	-35.4604070000	26.0301090000
O53	-1.0619030000	-35.4794690000	27.2024970000
N54	-5.1300380000	-32.3014830000	25.0728490000
C55	-4.6203960000	-33.0826070000	26.0778160000
C56	-5.5683030000	-33.1371500000	27.1848560000
C57	-6.6658670000	-32.4091610000	26.8044610000
C58	-6.3630060000	-31.8718130000	25.4693600000
C59	-5.3504810000	-33.9076190000	28.4530910000
N60	-2.7529380000	-33.4725760000	23.6753250000
C61	-7.0229120000	-30.6874830000	23.3420490000
C62	-7.9410740000	-29.8590300000	22.5616070000
C63	-7.5010910000	-29.9145240000	21.2537020000
C64	-6.2807310000	-30.7264390000	21.2590560000
C65	-9.1048740000	-29.0898690000	23.1005540000
C66	-8.1068080000	-29.3601760000	20.0444430000
C67	-8.7577030000	-28.1916030000	19.9342540000
N68	-3.6975780000	-32.3732990000	21.1174830000
C69	-4.2791240000	-31.7196120000	20.0739610000
C70	-3.4417940000	-31.8651830000	18.8841380000
C71	-2.3632230000	-32.6269190000	19.2699510000
C72	-2.5332390000	-32.9447100000	20.6752470000
C73	-3.7373070000	-31.2966610000	17.5282900000
C74	-7.9535230000	-32.2672420000	27.5922870000
O75	-8.5603280000	-31.0041560000	27.4291900000
C76	-8.9631760000	-33.3657490000	27.1921690000
H77	0.0270000000	-35.2554090000	23.2749480000
H78	-1.5359090000	-32.9456250000	18.6451230000
H79	-1.2231130000	-26.6306340000	23.6720470000
H80	-0.0727390000	-27.7198140000	25.6075920000
H81	-0.4020670000	-26.3009240000	26.6113720000
H82	-8.5386710000	-24.7345980000	23.8832210000
H83	-6.6104670000	-23.3832450000	25.8403630000

H84	-8.3404380000	-23.5796130000	26.0830000000
H85	3.6424350000	-34.0718350000	28.5700230000
H86	1.2781860000	-34.6946450000	28.1278510000
H87	1.4076790000	-33.5992970000	29.5003470000
H88	3.7239740000	-33.5178830000	24.0867100000
H89	4.5009000000	-30.5427590000	24.2576900000
H90	4.5140940000	-31.4543880000	22.7604370000
H91	-7.9669130000	-38.0948490000	21.2593840000
H92	-5.6948640000	-38.5422940000	22.2316860000
H93	-4.9270890000	-37.8301580000	20.8202340000
H94	-6.0285640000	-33.5758060000	29.2451950000
H95	-4.3232330000	-33.7892150000	28.8179170000
H96	-5.5141450000	-34.9842220000	28.3086570000
H97	-9.9075870000	-29.0321010000	22.3561590000
H98	-8.8116500000	-28.0576800000	23.3338950000
H99	-9.5013280000	-29.5327430000	24.0191400000
H100	-2.9266970000	-31.5095420000	16.8238470000
H101	-3.8756330000	-30.2082540000	17.5656070000
H102	-4.6618900000	-31.7174530000	17.1124310000
H103	-7.6997600000	-32.4302830000	28.6556910000
H104	-8.4634600000	-34.3410910000	27.1497420000
H105	-9.3593210000	-33.1318400000	26.1972750000
H106	-9.8097920000	-33.4561000000	27.8942510000
H107	-0.7868410000	-34.1139410000	20.9707910000
H108	-5.8073810000	-30.5381700000	19.2044530000
H109	-8.0040960000	-29.9802000000	19.1521190000
H110	-8.8306500000	-27.4830950000	20.7516420000
H111	-9.1868910000	-27.8911480000	18.9811400000
H112	-8.0750200000	-30.6734520000	25.1960740000
H113	-3.1180560000	-34.3220370000	26.9215470000
H114	0.2469810000	-35.9649390000	25.7391660000
H115	-7.1981910000	-34.1660310000	24.0161930000
H116	-7.6887790000	-36.5447920000	23.1910930000
H117	-4.1121960000	-35.2236750000	21.3929020000
H118	-0.3151040000	-31.5514740000	22.1074470000
H119	2.1014630000	-32.3246080000	21.8880960000
H120	1.8849700000	-29.6803190000	25.1452030000
H121	4.2488620000	-31.8385960000	26.4621110000
H122	0.8240470000	-32.6740490000	25.5561540000
H123	-0.5801600000	-32.1608700000	29.4666670000
H124	-2.0741560000	-30.7719080000	27.9043540000
H125	-4.4366740000	-29.7146570000	25.8434260000
H126	-3.3370490000	-25.8020670000	26.9444080000
H127	-5.1225940000	-25.3192880000	25.7946660000
H128	-8.8226130000	-28.2471160000	27.2485430000
H129	-9.2866330000	-25.9191230000	26.5624430000
H130	-7.8134590000	-30.3300270000	27.4678100000
H131	3.2712150000	-32.3595430000	28.3144210000
H132	-1.4218420000	-25.2221090000	24.5788000000
H133	0.0891600000	-25.7121050000	24.1996750000

H134	-8.0232120000	-23.1343480000	23.7900910000
N135	-6.6127130000	-24.4831010000	23.2785890000
H136	5.2637250000	-32.9382820000	24.4987580000
H137	-7.2148290000	-37.3638730000	19.8263470000
H138	-6.9903140000	-39.0944710000	20.1643680000
H139	-4.7015930000	-25.0781210000	21.7306900000
C140	-5.5859970000	-25.6409850000	21.3980940000
C141	-6.8178830000	-25.2897150000	22.2048090000
O142	-7.9253640000	-25.7408010000	21.9116500000
C143	-5.3133290000	-27.1567130000	21.4831850000
C144	-4.1806730000	-27.5690610000	20.5371440000
C145	-5.0298440000	-27.6096210000	22.9231820000
H146	-5.6832160000	-24.1396010000	23.4700450000
H147	-5.7846840000	-25.3626800000	20.3562700000
H148	-6.2278290000	-27.6535890000	21.1489240000
H149	-3.2321770000	-27.0950300000	20.8354190000
H150	-4.3915330000	-27.2720360000	19.5025230000
H151	-4.0625930000	-28.6584510000	20.5539130000
H152	-5.8065250000	-27.2903440000	23.6238770000
H153	-4.0697640000	-27.2199100000	23.2909830000
H154	-5.0076420000	-28.7036440000	22.9599550000
H155	-2.6108300000	-30.3527890000	22.4297900000
C156	-11.0220150000	-37.0394900000	21.4210340000
C157	-10.4434740000	-36.8211400000	22.8066160000
O158	-9.3475300000	-37.2601090000	23.1661870000
N159	-11.2531430000	-36.1398050000	23.6516360000
C160	-10.8110020000	-35.7070310000	24.9628930000
H161	-10.3648300000	-37.6849400000	20.8387390000
H162	-11.1665010000	-36.0900080000	20.8944020000
H163	-12.1029020000	-35.7382430000	23.2815320000
H164	-10.0147120000	-34.9573140000	24.8879050000
H165	-11.6555690000	-35.2556460000	25.4883690000
H166	-11.9911170000	-37.5204510000	21.5567780000
H167	-10.4290590000	-36.5298540000	25.5672440000
O168	-1.7223810000	-29.8945600000	20.9517770000
O169	-3.0144710000	-30.5224630000	23.3184990000
H170	-2.0305730000	-29.0115830000	20.6976990000
H171	-3.5775770000	-29.7513790000	23.5009730000
H172	-2.0365210000	-30.4896790000	20.2450140000

R (quintet, +1)

C1	-0.8190010000	-26.0150340000	24.4889950000
C2	-0.7488210000	-26.8613130000	25.7789690000
C3	-2.0953730000	-27.4280930000	26.0987800000
N4	-2.4856590000	-28.7024820000	25.7107520000
C5	-3.1831940000	-26.7757700000	26.6080590000
C6	-3.7831280000	-28.8070950000	25.9752580000
N7	-4.2582190000	-27.6465640000	26.5136220000

C8	-7.7230160000	-24.0380170000	24.0959740000
C9	-7.4787380000	-24.0523450000	25.6152740000
C10	-7.2168880000	-25.4374220000	26.1548520000
C11	-8.2326050000	-26.2885800000	26.6077460000
C12	-5.9173650000	-25.9366530000	26.1383570000
C13	-7.9460480000	-27.5790810000	27.0562840000
C14	-5.6147640000	-27.2187670000	26.5918850000
C15	-6.6287950000	-28.0621110000	27.0741790000
O16	-6.2737310000	-29.2805790000	27.5373880000
C17	2.9849940000	-33.3740010000	28.0370010000
C18	3.1665850000	-33.5759700000	26.5339530000
O19	2.6654660000	-34.5227810000	25.9338760000
C20	1.5152010000	-33.6547660000	28.4124550000
C21	0.5355410000	-32.7248000000	27.7524380000
N22	-0.4546000000	-32.0821840000	28.4737280000
C23	0.3127340000	-32.3350450000	26.4515710000
C24	-1.2208010000	-31.3529010000	27.6234590000
N25	-0.7721240000	-31.4819600000	26.3893810000
N26	3.8839680000	-32.5972440000	25.9092810000
C27	4.2949810000	-32.6899990000	24.5160050000
C28	4.0344970000	-31.3773230000	23.7449070000
C29	2.5820500000	-31.0661560000	23.5712130000
N30	1.7960980000	-31.6816020000	22.6062580000
C31	1.7086670000	-30.2677170000	24.2649990000
C32	0.5175520000	-31.2423920000	22.7429640000
N33	0.4295260000	-30.3850690000	23.7461220000
C34	-7.0756620000	-38.0454900000	20.6175520000
C35	-5.8218350000	-37.7166370000	21.4469320000
C36	-5.8678700000	-36.3646930000	22.0879290000
N37	-6.8874650000	-35.9627340000	22.9351160000
C38	-5.0179350000	-35.2891200000	22.0217250000
C39	-6.6307120000	-34.6950720000	23.3339840000
N40	-5.5027100000	-34.2583500000	22.8013690000
Cu41	-1.3107700000	-30.1710740000	24.8498990000
Fe42	-4.4802240000	-32.3598560000	23.1816480000
C43	-1.7449910000	-33.6494600000	21.4981860000
C44	-3.3732660000	-33.7608720000	26.0949440000
C45	-7.2107690000	-31.0395410000	24.8545190000
C46	-5.5750350000	-30.9017430000	20.2829860000
N47	-6.0777470000	-31.1120010000	22.6755770000
C48	-1.8163890000	-33.9166760000	22.8805680000
C49	-0.9117940000	-34.7536740000	23.6226290000
C50	-1.3690220000	-34.7890170000	24.9249740000
C51	-2.5565930000	-33.9515270000	24.9679740000
C52	-0.6769870000	-35.4679150000	26.0170270000
O53	-1.0247630000	-35.4703250000	27.1926440000
N54	-5.1120210000	-32.2578580000	25.1957550000
C55	-4.5698810000	-33.0452080000	26.1779020000
C56	-5.4890660000	-33.1218190000	27.3049410000
C57	-6.6092910000	-32.4103620000	26.9459570000

C58	-6.3505380000	-31.8588750000	25.6115930000
C59	-5.2248450000	-33.9034540000	28.5573250000
N60	-2.7805220000	-33.4401890000	23.7305620000
C61	-7.0630930000	-30.6585200000	23.5122130000
C62	-7.9789340000	-29.7944470000	22.7756520000
C63	-7.5658460000	-29.8185830000	21.4576070000
C64	-6.3498820000	-30.6441440000	21.4215700000
C65	-9.0683390000	-28.9647540000	23.3839130000
C66	-8.1358960000	-29.1509150000	20.2945460000
C67	-9.3837620000	-28.6753920000	20.1623100000
N68	-3.7873250000	-32.3208270000	21.2018970000
C69	-4.3874590000	-31.6513020000	20.1810050000
C70	-3.5845750000	-31.7974190000	18.9661520000
C71	-2.5056250000	-32.5733950000	19.3181920000
C72	-2.6414310000	-32.8983040000	20.7257230000
C73	-3.9141590000	-31.2231540000	17.6209200000
C74	-7.8868960000	-32.3666500000	27.7386610000
O75	-8.4027770000	-31.0080430000	27.7199880000
C76	-8.9464500000	-33.3518710000	27.2144720000
H77	-1.7008940000	-32.8998490000	18.6693820000
H78	3.2712800000	-32.3595120000	28.3144130000
H79	-1.2227900000	-26.6448970000	23.6838190000
H80	-0.0434620000	-27.6890470000	25.6382540000
H81	-0.3797070000	-26.2518600000	26.6131100000
H82	-8.5351560000	-24.7340850000	23.8669090000
H83	-6.6401080000	-23.3880040000	25.8656180000
H84	-8.3668970000	-23.6186120000	26.0912190000
H85	3.6408980000	-34.0776560000	28.5626510000
H86	1.2692630000	-34.6839490000	28.1304090000
H87	1.4044500000	-33.5871660000	29.5013980000
H88	3.7206580000	-33.5138210000	24.0844190000
H89	4.5156460000	-30.5425380000	24.2688290000
H90	4.5298880000	-31.4462470000	22.7667450000
H91	-7.9646260000	-38.0623550000	21.2558670000
H92	-5.6858040000	-38.4899410000	22.2163720000
H93	-4.9298200000	-37.7543750000	20.8113690000
H94	-5.3176510000	-34.9834480000	28.3854870000
H95	-5.9144050000	-33.6375120000	29.3643880000
H96	-4.2058440000	-33.7280770000	28.9203380000
H97	-10.0616510000	-29.4194580000	23.2652590000
H98	-9.0907600000	-27.9766960000	22.9088570000
H99	-8.8970980000	-28.8124100000	24.4545880000
H100	-3.1254220000	-31.4401050000	16.8942720000
H101	-4.0435150000	-30.1340710000	17.6641200000
H102	-4.8517420000	-31.6388450000	17.2307830000
H103	-7.6530230000	-32.6264800000	28.7793250000
H104	-8.4977150000	-34.3449900000	27.1065750000
H105	-9.2995630000	-33.0225720000	26.2318570000
H106	-9.8097290000	-33.4560170000	27.8941730000
H107	-0.9008540000	-34.0862580000	20.9693360000

H108	-5.9259990000	-30.4625990000	19.3549270000
H109	-7.4647420000	-29.0308130000	19.4446660000
H110	-10.1327230000	-28.7673760000	20.9406430000
H111	-9.6918870000	-28.1733490000	19.2503240000
H112	-8.1028210000	-30.6920810000	25.3618180000
H113	-3.0494140000	-34.2956770000	26.9801960000
H114	0.2551610000	-35.9852560000	25.7129670000
H115	-7.2602710000	-34.1253240000	24.0018920000
H116	-7.7348560000	-36.4927370000	23.1451890000
H117	-4.0926050000	-35.1862980000	21.4765970000
H118	-0.2968540000	-31.5521450000	22.1095710000
H119	2.1203190000	-32.3341370000	21.9068030000
H120	1.9074160000	-29.6390530000	25.1201740000
H121	4.3302250000	-31.8935510000	26.4816060000
H122	0.8352100000	-32.6291960000	25.5574950000
H123	-0.5801650000	-32.1608890000	29.4668010000
H124	-2.0643930000	-30.7499050000	27.9247890000
H125	-4.3990610000	-29.6781690000	25.8023170000
H126	-3.2940280000	-25.7864650000	27.0222590000
H127	-5.1196840000	-25.3252770000	25.7313800000
H128	-8.7429430000	-28.2248950000	27.4135490000
H129	-9.2633880000	-25.9428480000	26.6140800000
H130	-9.1613490000	-30.9590800000	28.3233490000
H131	-0.0396570000	-35.2625890000	23.2284810000
H132	-1.4217640000	-25.2221200000	24.5787620000
H133	0.0891110000	-25.7122690000	24.1996400000
H134	-8.0231180000	-23.1344260000	23.7901100000
N135	-6.6052740000	-24.4870640000	23.2904150000
H136	5.2635660000	-32.9382630000	24.4989890000
H137	-7.2289950000	-37.2964710000	19.8321550000
H138	-6.9770660000	-39.0275190000	20.1423090000
H139	-4.7122550000	-25.0542140000	21.7155360000
C140	-5.5860130000	-25.6410260000	21.3980200000
C141	-6.8080550000	-25.3415130000	22.2407820000
O142	-7.8841350000	-25.8899560000	22.0223260000
C143	-5.2761390000	-27.1493510000	21.4787060000
C144	-4.1090190000	-27.5334240000	20.5648610000
C145	-5.0299920000	-27.6096400000	22.9232670000
H146	-5.7399990000	-23.9666520000	23.3258670000
H147	-5.8116740000	-25.3673900000	20.3608460000
H148	-6.1762630000	-27.6579340000	21.1254020000
H149	-3.1790930000	-27.0444810000	20.8933330000
H150	-4.2938380000	-27.2323440000	19.5267120000
H151	-3.9641290000	-28.6192700000	20.5809230000
H152	-5.8411090000	-27.3142320000	23.5943300000
H153	-4.0913420000	-27.2028600000	23.3261010000
H154	-4.9901180000	-28.7028600000	22.9490970000
H155	-3.5513190000	-29.6837160000	23.4818550000
C156	-11.0220700000	-37.0398980000	21.4210000000
C157	-10.4479920000	-36.8087010000	22.8045900000

O158	-9.3420660000	-37.2210920000	23.1666260000
N159	-11.2607260000	-36.1353720000	23.6539840000
C160	-10.8109180000	-35.7070120000	24.9629590000
H161	-10.3630350000	-37.6892890000	20.8455200000
H162	-11.1670650000	-36.0944600000	20.8876210000
H163	-12.1432300000	-35.7858620000	23.3089660000
H164	-10.0103350000	-34.9609380000	24.8791450000
H165	-11.6517580000	-35.2517320000	25.4915520000
H166	-11.9911000000	-37.5201190000	21.5567400000
H167	-10.4291310000	-36.5298840000	25.5672450000
O168	-3.0193180000	-30.4860040000	23.3561190000
H169	-2.6152580000	-30.3825050000	22.4566940000
H170	-7.0703210000	-29.8394360000	27.6916540000
O171	-1.7196530000	-29.9426920000	20.9854760000
H172	-1.8388870000	-29.0087930000	20.7567020000
H173	-2.0703590000	-30.4487460000	20.2285690000

A (singlet, +1)

C1	-0.8190210000	-26.0150640000	24.4889620000
C2	-0.7295080000	-26.8027800000	25.8175980000
C3	-2.0667180000	-27.3816990000	26.1484640000
N4	-2.4411600000	-28.6505450000	25.7295000000
C5	-3.1691740000	-26.7450980000	26.6436210000
C6	-3.7426040000	-28.7660480000	25.9485130000
N7	-4.2359780000	-27.6237550000	26.5034660000
C8	-7.7230420000	-24.0380500000	24.0959570000
C9	-7.4872170000	-24.0596750000	25.6165070000
C10	-7.2168750000	-25.4481490000	26.1420840000
C11	-8.2273510000	-26.3197000000	26.5652100000
C12	-5.9109170000	-25.9302390000	26.1335180000
C13	-7.9297180000	-27.6166920000	26.9892220000
C14	-5.5973090000	-27.2163910000	26.5640700000
C15	-6.6074350000	-28.0841710000	27.0098230000
O16	-6.2438700000	-29.3142110000	27.4418980000
C17	2.9849930000	-33.3739930000	28.0369990000
C18	3.1637510000	-33.5703160000	26.5318830000
O19	2.6588670000	-34.5138700000	25.9293160000
C20	1.5139160000	-33.6548420000	28.4105970000
C21	0.5348530000	-32.7227940000	27.7506080000
N22	-0.4543240000	-32.0788230000	28.4730450000
C23	0.3182590000	-32.3234140000	26.4512290000
C24	-1.2114380000	-31.3372190000	27.6246760000
N25	-0.7597510000	-31.4608080000	26.3903140000
N26	3.8799150000	-32.5899580000	25.9072190000
C27	4.2950000000	-32.6899950000	24.5159970000
C28	4.0790250000	-31.3745190000	23.7357520000
C29	2.6439780000	-30.9969640000	23.5732540000
N30	1.8345090000	-31.4949070000	22.5608250000

C31	1.8051910000	-30.2300470000	24.3397060000
C32	0.5733560000	-31.0210290000	22.7425800000
N33	0.5256740000	-30.2552170000	23.8196390000
C34	-6.9648540000	-37.8164020000	20.4087810000
C35	-5.7343220000	-37.5154270000	21.2816120000
C36	-5.7722020000	-36.1588440000	21.9128610000
N37	-6.8618320000	-35.6966550000	22.6326450000
C38	-4.8538230000	-35.1424180000	21.9620320000
C39	-6.5853960000	-34.4571270000	23.0817220000
N40	-5.3713590000	-34.0917850000	22.6953370000
Cu41	-1.2419620000	-30.0568010000	24.8581100000
Fe42	-4.4342910000	-32.3815420000	23.1902690000
C43	-1.6091520000	-33.4843750000	21.5774730000
C44	-3.4515920000	-33.9672510000	26.0446830000
C45	-7.2188790000	-31.1800380000	24.8413850000
C46	-5.5375250000	-30.9584010000	20.3188360000
N47	-6.0596130000	-31.2867130000	22.6830220000
C48	-1.7596360000	-33.8254430000	22.9214590000
C49	-0.8394890000	-34.6272510000	23.6635000000
C50	-1.3690190000	-34.7890470000	24.9250070000
C51	-2.6160330000	-34.0558280000	24.9373210000
C52	-0.6721330000	-35.4517670000	26.0264970000
O53	-1.0754620000	-35.5488700000	27.1795520000
N54	-5.1951630000	-32.5321270000	25.0753940000
C55	-4.6473260000	-33.2750090000	26.0921540000
C56	-5.5191830000	-33.2713090000	27.2506180000
C57	-6.6141350000	-32.5232700000	26.9064600000
C58	-6.3836290000	-32.0421870000	25.5489940000
C59	-5.2278470000	-34.0084110000	28.5232240000
N60	-2.8303660000	-33.4820250000	23.7155110000
C61	-7.0347330000	-30.7965280000	23.5188920000
C62	-7.9134780000	-29.8871940000	22.8066540000
C63	-7.5061760000	-29.9062460000	21.4903960000
C64	-6.3202750000	-30.7631820000	21.4470210000
C65	-8.9691670000	-29.0298120000	23.4352840000
C66	-8.0419030000	-29.2014580000	20.3336960000
C67	-9.2207190000	-28.5669440000	20.2377590000
N68	-3.7165880000	-32.2624360000	21.3226050000
C69	-4.3218050000	-31.6316090000	20.2737050000
C70	-3.4924780000	-31.7173650000	19.0774780000
C71	-2.3803190000	-32.4131580000	19.4465850000
C72	-2.5243140000	-32.7513580000	20.8424550000
C73	-3.8253200000	-31.1245860000	17.7435760000
C74	-7.8742070000	-32.4187280000	27.7166440000
O75	-8.3611710000	-31.0524830000	27.6840320000
C76	-8.9531550000	-33.3881070000	27.2091940000
H77	0.1010560000	-35.0165600000	23.2926620000
H78	-1.5317210000	-32.6815640000	18.8286300000
H79	-1.2255660000	-26.6723350000	23.7100090000
H80	-0.0015770000	-27.6174640000	25.7172890000

H81	-0.3791050000	-26.1459290000	26.6233440000
H82	-8.5353380000	-24.7330970000	23.8630520000
H83	-6.6547310000	-23.3917580000	25.8770750000
H84	-8.3817920000	-23.6368430000	26.0907330000
H85	3.6408640000	-34.0790330000	28.5605160000
H86	1.2701730000	-34.6848530000	28.1292190000
H87	1.4021670000	-33.5877910000	29.4994240000
H88	3.7135640000	-33.5054050000	24.0795120000
H89	4.6031690000	-30.5588020000	24.2489150000
H90	4.5641750000	-31.4742640000	22.7555220000
H91	-7.8777780000	-37.8083950000	21.0137830000
H92	-5.6503740000	-38.2844090000	22.0626140000
H93	-4.8191140000	-37.5829240000	20.6830010000
H94	-5.9181080000	-33.7327730000	29.3257030000
H95	-4.2109890000	-33.7982480000	28.8737700000
H96	-5.2971980000	-35.0945930000	28.3838250000
H97	-9.9841690000	-29.4058910000	23.2466750000
H98	-8.9051470000	-28.0131280000	23.0283130000
H99	-8.8333100000	-28.9552170000	24.5182110000
H100	-3.0403180000	-31.3426700000	17.0134930000
H101	-3.9319210000	-30.0341190000	17.8070140000
H102	-4.7700280000	-31.5196930000	17.3495310000
H103	-7.6344330000	-32.6707460000	28.7575230000
H104	-8.5288890000	-34.3932690000	27.1166990000
H105	-9.3028240000	-33.0656780000	26.2232480000
H106	-9.8097600000	-33.4561000000	27.8942240000
H107	-0.7192520000	-33.8410420000	21.0668070000
H108	-5.8732020000	-30.5118940000	19.3914150000
H109	-7.4029500000	-29.1877440000	19.4528830000
H110	-9.9386720000	-28.5220510000	21.0472300000
H111	-9.4964250000	-28.0563760000	19.3203200000
H112	-8.0773390000	-30.7852040000	25.3673800000
H113	-3.1205820000	-34.4976310000	26.9282800000
H114	0.3235990000	-35.8517880000	25.7506070000
H115	-7.2551970000	-33.8514820000	23.6714990000
H116	-7.7428250000	-36.1973690000	22.7820640000
H117	-3.8671970000	-35.0917740000	21.5317570000
H118	-0.2726040000	-31.2653710000	22.1178320000
H119	2.1365200000	-32.0863800000	21.7997820000
H120	2.0300530000	-29.6851690000	25.2444480000
H121	4.3385120000	-31.8958760000	26.4819050000
H122	0.8448590000	-32.6113470000	25.5575330000
H123	-0.5801670000	-32.1608890000	29.4667910000
H124	-2.0472270000	-30.7238160000	27.9278320000
H125	-4.3500370000	-29.6178510000	25.6916870000
H126	-3.2987770000	-25.7632030000	27.0693280000
H127	-5.1157680000	-25.3015920000	25.7491650000
H128	-8.7236250000	-28.2792840000	27.3228660000
H129	-9.2625400000	-25.9871480000	26.5651450000
H130	-9.1136430000	-30.9794330000	28.2924800000

H131	3.2712790000	-32.3595120000	28.3144110000
H132	-1.4217560000	-25.2221240000	24.5787680000
H133	0.0891100000	-25.7122520000	24.1996770000
H134	-8.0230980000	-23.1344300000	23.7901230000
N135	-6.6067460000	-24.4877760000	23.2894670000
H136	5.2635510000	-32.9382670000	24.4989950000
H137	-7.0719000000	-37.0710560000	19.6126350000
H138	-6.8750300000	-38.8044050000	19.9451470000
H139	-4.7227760000	-25.0351850000	21.7076590000
C140	-5.5860030000	-25.6409930000	21.3980010000
C141	-6.8021790000	-25.3655470000	22.2558120000
O142	-7.8723600000	-25.9375100000	22.0622580000
C143	-5.2273040000	-27.1448170000	21.4738670000
C144	-3.9973860000	-27.4681210000	20.6183610000
C145	-5.0299090000	-27.6096400000	22.9231950000
H146	-5.7458590000	-23.9597360000	23.3117920000
H147	-5.8284500000	-25.3705440000	20.3634590000
H148	-6.0897740000	-27.6808320000	21.0672720000
H149	-3.1046960000	-26.9553930000	21.0015810000
H150	-4.1383400000	-27.1601700000	19.5742890000
H151	-3.7931090000	-28.5451760000	20.6349790000
H152	-5.9388870000	-27.4779910000	23.5181540000
H153	-4.2221800000	-27.0450100000	23.4107230000
H154	-4.7524230000	-28.6672310000	22.9590320000
H155	-7.0372010000	-29.8709470000	27.6107500000
C156	-11.0219920000	-37.0395550000	21.4210640000
C157	-10.4330490000	-36.7285420000	22.7785090000
O158	-9.2591250000	-36.9694520000	23.0845180000
N159	-11.2774800000	-36.1567990000	23.6652070000
C160	-10.8110160000	-35.7070390000	24.9629140000
H161	-10.3564530000	-37.7095910000	20.8758260000
H162	-11.1626990000	-36.1216050000	20.8401640000
H163	-12.2331260000	-35.9750790000	23.3943770000
H164	-10.0026160000	-34.9760740000	24.8485580000
H165	-11.6404810000	-35.2340550000	25.4926220000
H166	-11.9911370000	-37.5203900000	21.5567550000
H167	-10.4290790000	-36.5298610000	25.5672320000
O168	-2.4012000000	-30.5996550000	23.2159790000
O169	-3.6303610000	-30.7912010000	23.6518460000

A$_{R}$ (doublet, 0)

C1	-0.8190210000	-26.0150640000	24.4889620000
C2	-0.7280690000	-26.7583890000	25.8454110000
C3	-2.0646600000	-27.3282810000	26.1915870000
N4	-2.4308110000	-28.5930140000	25.7599510000
C5	-3.1693850000	-26.7028240000	26.6964490000
C6	-3.7285840000	-28.7240160000	25.9753250000
N7	-4.2301920000	-27.5913600000	26.5515250000

C8	-7.7230420000	-24.0380500000	24.0959570000
C9	-7.4965670000	-24.0583920000	25.6177560000
C10	-7.2245320000	-25.4434510000	26.1516060000
C11	-8.2330270000	-26.3255580000	26.5569500000
C12	-5.9139750000	-25.9127460000	26.1645640000
C13	-7.9282620000	-27.6187400000	26.9883920000
C14	-5.5927970000	-27.1948950000	26.5995540000
C15	-6.6012490000	-28.0722010000	27.0340210000
O16	-6.2285520000	-29.2906210000	27.4810580000
C17	2.9849930000	-33.3739930000	28.0369990000
C18	3.2258950000	-33.6080820000	26.5481010000
O19	2.8499530000	-34.6281090000	25.9782330000
C20	1.5066020000	-33.6443600000	28.3832070000
C21	0.5530320000	-32.6753730000	27.7454590000
N22	-0.4377250000	-32.0424120000	28.4763830000
C23	0.3656960000	-32.2284700000	26.4571840000
C24	-1.1708680000	-31.2643780000	27.6333010000
N25	-0.6978260000	-31.3492950000	26.4055420000
N26	3.8671170000	-32.5910910000	25.9018540000
C27	4.2950000000	-32.6899950000	24.5159970000
C28	4.0703630000	-31.3817210000	23.7310500000
C29	2.6349610000	-31.0002190000	23.5900960000
N30	1.7535030000	-31.6456910000	22.7325970000
C31	1.8616870000	-30.0776310000	24.2474710000
C32	0.5160890000	-31.1021310000	22.8976060000
N33	0.5543220000	-30.1485120000	23.8141330000
C34	-6.9921720000	-37.9247440000	20.5292570000
C35	-5.7653810000	-37.5985720000	21.3992630000
C36	-5.8191970000	-36.2382430000	22.0209120000
N37	-6.8776560000	-35.8075870000	22.8036020000
C38	-4.9415100000	-35.1830670000	21.9983710000
C39	-6.6111890000	-34.5431100000	23.2138960000
N40	-5.4430570000	-34.1382710000	22.7474500000
Cu41	-1.2631070000	-29.9704970000	24.8337960000
Fe42	-4.4826920000	-32.4040600000	23.2040250000
C43	-1.7028320000	-33.5430640000	21.5552430000
C44	-3.4246090000	-33.9205740000	26.0803760000
C45	-7.2544620000	-31.2233910000	24.8650260000
C46	-5.5878060000	-30.9403800000	20.3403590000
N47	-6.1050460000	-31.2938120000	22.7000770000
C48	-1.8267480000	-33.8749730000	22.9096700000
C49	-0.8859870000	-34.6672290000	23.6373210000
C50	-1.3690190000	-34.7890470000	24.9250070000
C51	-2.6159190000	-34.0445400000	24.9532010000
C52	-0.6407350000	-35.4279330000	26.0076620000
O53	-0.9862800000	-35.5046350000	27.1853620000
N54	-5.2046750000	-32.5355990000	25.1062490000
C55	-4.6295830000	-33.2387730000	26.1351410000
C56	-5.4869720000	-33.2245900000	27.3072240000
C57	-6.6015740000	-32.5108150000	26.9559040000

C58	-6.3947120000	-32.0596690000	25.5803620000
C59	-5.1666760000	-33.9247440000	28.5942340000
N60	-2.8699020000	-33.5046120000	23.7263510000
C61	-7.0897930000	-30.8346230000	23.5386050000
C62	-7.9892110000	-29.9315850000	22.8350790000
C63	-7.5749320000	-29.9189320000	21.5223850000
C64	-6.3730780000	-30.7588250000	21.4724880000
C65	-9.0653160000	-29.1024280000	23.4682060000
C66	-8.1206350000	-29.2027300000	20.3784070000
C67	-9.3327450000	-28.6352830000	20.2642120000
N68	-3.7887940000	-32.2847560000	21.3204040000
C69	-4.3784750000	-31.6284370000	20.2818950000
C70	-3.5526730000	-31.7127840000	19.0771180000
C71	-2.4582580000	-32.4385910000	19.4310840000
C72	-2.6096040000	-32.7899860000	20.8293300000
C73	-3.8766090000	-31.0890790000	17.7544860000
C74	-7.8621970000	-32.4234010000	27.7597270000
O75	-8.3474270000	-31.0506970000	27.7652850000
C76	-8.9462350000	-33.3699610000	27.2186130000
H77	0.0366300000	-35.0793460000	23.2437060000
H78	-1.6142510000	-32.7132000000	18.8082240000
H79	-1.2256040000	-26.6945530000	23.7310370000
H80	-0.0036920000	-27.5787260000	25.7734660000
H81	-0.3795940000	-26.0706140000	26.6268710000
H82	-8.5361950000	-24.7317010000	23.8601340000
H83	-6.6666200000	-23.3875220000	25.8817200000
H84	-8.3948830000	-23.6347690000	26.0855370000
H85	3.6228680000	-34.0758250000	28.5871140000
H86	1.2497660000	-34.6630060000	28.0723380000
H87	1.3853270000	-33.6074370000	29.4731670000
H88	3.7210170000	-33.5106470000	24.0767360000
H89	4.6002070000	-30.5603600000	24.2305490000
H90	4.5455030000	-31.4912170000	22.7457640000
H91	-7.9029490000	-37.9256100000	21.1372220000
H92	-5.6673260000	-38.3664780000	22.1807710000
H93	-4.8516000000	-37.6548770000	20.7966230000
H94	-5.8606930000	-33.6526340000	29.3961890000
H95	-4.1542510000	-33.6750530000	28.9334410000
H96	-5.2000360000	-35.0163230000	28.4816880000
H97	-10.0713790000	-29.5176660000	23.3099800000
H98	-9.0527770000	-28.0918580000	23.0410840000
H99	-8.9104500000	-29.0038600000	24.5471590000
H100	-3.0958440000	-31.3051610000	17.0178360000
H101	-3.9670430000	-29.9977910000	17.8376600000
H102	-4.8291100000	-31.4591180000	17.3525430000
H103	-7.6346400000	-32.7056270000	28.7962340000
H104	-8.5229450000	-34.3727490000	27.0996020000
H105	-9.2844760000	-33.0189060000	26.2388800000
H106	-9.8097600000	-33.4561000000	27.8942240000
H107	-0.8253310000	-33.9149670000	21.0311360000

H108	-5.9229310000	-30.4779280000	19.4191570000
H109	-7.4572590000	-29.1198580000	19.5185470000
H110	-10.0774660000	-28.6689550000	21.0507890000
H111	-9.6092440000	-28.1015830000	19.3596780000
H112	-8.1164050000	-30.8392540000	25.3952350000
H113	-3.0690630000	-34.4223250000	26.9718760000
H114	0.3412890000	-35.8442310000	25.6977560000
H115	-7.2622270000	-33.9533810000	23.8403970000
H116	-7.7215600000	-36.3372080000	23.0136390000
H117	-3.9840000000	-35.1033290000	21.5090680000
H118	-0.3795390000	-31.4327370000	22.3891320000
H119	1.9793630000	-32.4020000000	22.1026840000
H120	2.1598910000	-29.3778970000	25.0155260000
H121	4.1434950000	-31.7762990000	26.4305230000
H122	0.9058110000	-32.4918710000	25.5623530000
H123	-0.5801670000	-32.1608890000	29.4667910000
H124	-2.0108010000	-30.6574120000	27.9380300000
H125	-4.3240000000	-29.5768810000	25.6896460000
H126	-3.3079040000	-25.7273690000	27.1348130000
H127	-5.1175680000	-25.2782290000	25.7917400000
H128	-8.7196520000	-28.2926410000	27.3046910000
H129	-9.2723060000	-26.0055770000	26.5344220000
H130	-9.0861360000	-30.9875220000	28.3908180000
H131	3.2712790000	-32.3595120000	28.3144110000
H132	-1.4217560000	-25.2221240000	24.5787680000
H133	0.0891100000	-25.7122520000	24.1996770000
H134	-8.0230980000	-23.1344300000	23.7901230000
N135	-6.6075080000	-24.4946100000	23.2951850000
H136	5.2635510000	-32.9382670000	24.4989950000
H137	-7.1105070000	-37.1816900000	19.7322900000
H138	-6.8901080000	-38.9135930000	20.0671160000
H139	-4.7137330000	-25.0550920000	21.7228070000
C140	-5.5860030000	-25.6409930000	21.3980010000
C141	-6.8140870000	-25.3233780000	22.2220310000
O142	-7.9112090000	-25.8126070000	21.9674610000
C143	-5.2576860000	-27.1523300000	21.4775940000
C144	-4.0542100000	-27.5047680000	20.5960730000
C145	-5.0299090000	-27.6096400000	22.9231950000
H146	-5.7032600000	-24.0602490000	23.4074290000
H147	-5.8019440000	-25.3658710000	20.3585490000
H148	-6.1387770000	-27.6773450000	21.0960620000
H149	-3.1446450000	-27.0020810000	20.9528790000
H150	-4.2162200000	-27.2067110000	19.5514180000
H151	-3.8678740000	-28.5841500000	20.6234460000
H152	-5.9255730000	-27.4684910000	23.5374740000
H153	-4.2083390000	-27.0451850000	23.3889430000
H154	-4.7564160000	-28.6688310000	22.9626290000
H155	-7.0102310000	-29.8718550000	27.6221310000
C156	-11.0219920000	-37.0395550000	21.4210640000
C157	-10.4387430000	-36.7972490000	22.7989220000

O158	-9.3181250000	-37.1807860000	23.1424750000
N159	-11.2646260000	-36.1506310000	23.6590810000
C160	-10.8110160000	-35.7070390000	24.9629140000
H161	-10.3613710000	-37.6896860000	20.8478340000
H162	-11.1656100000	-36.0973590000	20.8808590000
H163	-12.1352910000	-35.7815250000	23.3044050000
H164	-10.0101950000	-34.9626310000	24.8741650000
H165	-11.6517740000	-35.2506330000	25.4907320000
H166	-11.9911370000	-37.5203900000	21.5567550000
H167	-10.4290790000	-36.5298610000	25.5672320000
O168	-2.3858170000	-30.6216390000	23.2079600000
O169	-3.6159700000	-30.7987100000	23.6966480000

F’ (doublet, +1)

C1	-0.8191000000	-26.0151690000	24.4889320000
C2	-0.7207900000	-26.8403800000	25.7963260000
C3	-2.0582830000	-27.4061510000	26.1645570000
N4	-2.4361370000	-28.7209380000	25.9165360000
C5	-3.1586710000	-26.7223170000	26.5986440000
C6	-3.7374970000	-28.8101690000	26.1592810000
N7	-4.2264310000	-27.6119230000	26.5782510000
C8	-7.7230370000	-24.0380360000	24.0959740000
C9	-7.4822770000	-24.0621410000	25.6173860000
C10	-7.2112770000	-25.4446160000	26.1661800000
C11	-8.2124890000	-26.2857270000	26.6676010000
C12	-5.9091340000	-25.9408090000	26.1405050000
C13	-7.9093770000	-27.5632650000	27.1463090000
C14	-5.5880320000	-27.2028710000	26.6358320000
C15	-6.5904880000	-28.0396920000	27.1555600000
O16	-6.2195600000	-29.2442950000	27.6478820000
C17	2.9850290000	-33.3740230000	28.0369720000
C18	3.1554190000	-33.5600930000	26.5285910000
O19	2.6351060000	-34.4913900000	25.9203170000
C20	1.4931890000	-33.6522900000	28.3819260000
C21	0.4951910000	-32.7230150000	27.7262520000
N22	-0.4475400000	-32.0305060000	28.4730490000
C23	0.2379880000	-32.3425370000	26.4283560000
C24	-1.1935330000	-31.2564140000	27.6403710000
N25	-0.7915440000	-31.4101450000	26.3874150000
N26	3.8885380000	-32.5883790000	25.9097480000
C27	4.2949170000	-32.6900560000	24.5160030000
C28	4.1384990000	-31.3567680000	23.7499690000
C29	2.7231650000	-30.9019550000	23.6313150000
N30	2.0118700000	-30.8689590000	22.4374500000
C31	1.8129870000	-30.5416560000	24.5887680000
C32	0.7314290000	-30.5076310000	22.7057720000
N33	0.5841450000	-30.3268990000	24.0060180000
C34	-7.0446160000	-37.0266000000	19.9117830000

C35	-5.7565470000	-36.8981210000	20.7437190000
C36	-5.7389200000	-35.6799960000	21.6132090000
N37	-6.7775270000	-35.3652230000	22.4731250000
C38	-4.8184610000	-34.6753960000	21.7819690000
C39	-6.4680540000	-34.2192120000	23.1132030000
N40	-5.2836140000	-33.7765500000	22.7214320000
Cu41	-1.3374490000	-30.0667510000	24.7532630000
Fe42	-4.3115660000	-32.0779000000	23.4799420000
C43	-1.4276620000	-32.9344480000	21.8438260000
C44	-3.4738160000	-34.0909880000	26.0958270000
C45	-7.1486900000	-31.0917210000	25.1330660000
C46	-5.4681650000	-30.5842970000	20.6364440000
N47	-5.9761790000	-31.0437760000	22.9818940000
C48	-1.6617350000	-33.5135310000	23.0899560000
C49	-0.7804400000	-34.4345250000	23.7320590000
C50	-1.3689110000	-34.7890430000	24.9250410000
C51	-2.6132610000	-34.0574340000	25.0035670000
C52	-0.6945670000	-35.5715790000	25.9602170000
O53	-1.0999590000	-35.7545200000	27.1015830000
N54	-5.1575690000	-32.5094260000	25.2675880000
C55	-4.6405260000	-33.3596230000	26.2130280000
C56	-5.5114610000	-33.4103090000	27.3719920000
C57	-6.5607040000	-32.5734600000	27.1001430000
C58	-6.3173590000	-32.0020680000	25.7822780000
C59	-5.2700140000	-34.2793240000	28.5697380000
N60	-2.7756850000	-33.3116870000	23.8718510000
C61	-6.9866990000	-30.6564370000	23.8265090000
C62	-7.9502450000	-29.8261530000	23.1248260000
C63	-7.5541130000	-29.8023800000	21.8081170000
C64	-6.2840050000	-30.5244030000	21.7534680000
C65	-9.0894210000	-29.0880340000	23.7574200000
C66	-8.2010680000	-29.1708150000	20.6640190000
C67	-9.5105430000	-28.9102020000	20.5446850000
N68	-3.5725930000	-31.7807410000	21.6293890000
C69	-4.1838160000	-31.1135200000	20.6068800000
C70	-3.2904400000	-31.0041770000	19.4607120000
C71	-2.1370270000	-31.6280040000	19.8292900000
C72	-2.3250110000	-32.1290630000	21.1695140000
C73	-3.6130230000	-30.3119530000	18.1736220000
C74	-7.8309160000	-32.4858360000	27.8917870000
O75	-8.2670360000	-31.1060370000	27.9634090000
C76	-8.9186810000	-33.3713340000	27.2601760000
H77	0.1822850000	-34.7564320000	23.3535800000
H78	-1.2387210000	-31.7622200000	19.2387330000
H79	-1.2159160000	-26.6818730000	23.7128260000
H80	-0.0149180000	-27.6665480000	25.6494120000
H81	-0.3315170000	-26.2210080000	26.6150590000
H82	-8.5373050000	-24.7324710000	23.8656580000
H83	-6.6491920000	-23.3928660000	25.8737010000
H84	-8.3750330000	-23.6360780000	26.0919190000

H85	3.6248600000	-34.0877800000	28.5668180000
H86	1.2630990000	-34.6817700000	28.0886730000
H87	1.3666100000	-33.5964050000	29.4697060000
H88	3.6963550000	-33.4834900000	24.0657670000
H89	4.7388340000	-30.5870480000	24.2536070000
H90	4.5867210000	-31.4815580000	22.7560690000
H91	-7.9204690000	-37.1128310000	20.5644510000
H92	-5.6355470000	-37.7988090000	21.3623100000
H93	-4.8827120000	-36.8574640000	20.0840610000
H94	-5.9314910000	-34.0214200000	29.4018500000
H95	-4.2377080000	-34.1842990000	28.9248090000
H96	-5.4284380000	-35.3398440000	28.3358500000
H97	-10.0338650000	-29.6476440000	23.7136480000
H98	-9.2355390000	-28.1342010000	23.2381020000
H99	-8.8806820000	-28.8644030000	24.8084560000
H100	-2.7805540000	-30.3927500000	17.4687060000
H101	-3.8131810000	-29.2459560000	18.3380600000
H102	-4.5020880000	-30.7402950000	17.6946070000
H103	-7.6333790000	-32.8385120000	28.9125840000
H104	-8.5258660000	-34.3828740000	27.1140020000
H105	-9.2063970000	-32.9670100000	26.2846160000
H106	-9.8097930000	-33.4561350000	27.8942260000
H107	-0.4910300000	-33.1762960000	21.3515090000
H108	-5.8276680000	-30.1235870000	19.7250400000
H109	-7.5547940000	-28.8955290000	19.8330330000
H110	-10.2298660000	-29.1720240000	21.3135150000
H111	-9.9011590000	-28.4161890000	19.6607230000
H112	-8.0105900000	-30.7342640000	25.6788790000
H113	-3.1685750000	-34.7258910000	26.9176770000
H114	0.3002700000	-35.9531820000	25.6547300000
H115	-7.1029510000	-33.7354620000	23.8382050000
H116	-7.6451900000	-35.8916740000	22.6010070000
H117	-3.8626890000	-34.5359540000	21.3022840000
H118	-0.0619340000	-30.3561670000	21.9903300000
H119	2.3951350000	-31.0387670000	21.5183410000
H120	1.9535500000	-30.4599550000	25.6537970000
H121	4.3901330000	-31.9303440000	26.4918790000
H122	0.7154920000	-32.6835370000	25.5274680000
H123	-0.5801510000	-32.1608510000	29.4668290000
H124	-1.9865380000	-30.5975970000	27.9612260000
H125	-4.3470230000	-29.6840150000	26.0009310000
H126	-3.2803770000	-25.6987090000	26.9137250000
H127	-5.1232730000	-25.3382380000	25.6997300000
H128	-8.6958870000	-28.1999680000	27.5428830000
H129	-9.2442690000	-25.9436740000	26.6904010000
H130	-9.0669880000	-31.0664500000	28.5110660000
H131	3.2711920000	-32.3595160000	28.3144280000
H132	-1.4216880000	-25.2220540000	24.5787930000
H133	0.0891290000	-25.7122140000	24.1996590000
H134	-8.0231070000	-23.1343920000	23.7901000000

N135	-6.6114350000	-24.4927160000	23.2843530000
H136	5.2636930000	-32.9382100000	24.4989740000
H137	-7.1837100000	-36.1501810000	19.2693180000
H138	-7.0073620000	-37.9175610000	19.2761820000
H139	-4.7497600000	-24.9893210000	21.6880020000
C140	-5.5860310000	-25.6410060000	21.3980060000
C141	-6.8004470000	-25.3981060000	22.2700120000
O142	-7.8493510000	-26.0154840000	22.1111180000
C143	-5.1324380000	-27.1179960000	21.4749660000
C144	-3.8094990000	-27.3181550000	20.7259690000
C145	-5.0298950000	-27.6096340000	22.9232080000
H146	-5.7628070000	-23.9443490000	23.2781010000
H147	-5.8524920000	-25.3909950000	20.3644290000
H148	-5.9114260000	-27.7067030000	20.9833240000
H149	-3.0020950000	-26.7422710000	21.1955510000
H150	-3.8869840000	-26.9990390000	19.6785300000
H151	-3.5063930000	-28.3701360000	20.7443430000
H152	-5.9855590000	-27.5261290000	23.4480300000
H153	-4.2795850000	-27.0333670000	23.4826410000
H154	-4.7285700000	-28.6608770000	22.9611090000
H155	-2.6040610000	-29.0866200000	23.0641230000
C156	-11.0220330000	-37.0394900000	21.4210300000
C157	-10.4135240000	-36.6174660000	22.7358740000
O158	-9.1968200000	-36.6414830000	22.9496750000
N159	-11.2806890000	-36.1724210000	23.6725330000
C160	-10.8110290000	-35.7070430000	24.9628890000
H161	-10.3458660000	-37.7337000000	20.9192640000
H162	-11.1570700000	-36.1636920000	20.7755990000
H163	-12.2727100000	-36.2096980000	23.4900040000
H164	-10.0041600000	-34.9804530000	24.8292660000
H165	-11.6376510000	-35.2286990000	25.4927440000
H166	-11.9911860000	-37.5204730000	21.5567680000
H167	-10.4290670000	-36.5298540000	25.5672380000
O168	-1.6359070000	-29.1068800000	23.1393070000
O169	-3.5202600000	-30.8035960000	24.1285020000
H170	-6.9984810000	-29.8123460000	27.8338580000

F’$_{\mathbf{R}}$ (triplet, 0)

C1	-0.8190970000	-26.0150850000	24.4890520000
C2	-0.6955820000	-26.8021950000	25.8179090000
C3	-2.0237460000	-27.3622550000	26.2156510000
N4	-2.3895180000	-28.6676120000	25.9307020000
C5	-3.1172100000	-26.6795160000	26.6740610000
C6	-3.6810530000	-28.7652680000	26.1856250000
N7	-4.1837350000	-27.5753970000	26.6460630000
C8	-7.7230970000	-24.0379980000	24.0959970000
C9	-7.4629420000	-24.0595090000	25.6135730000
C10	-7.1854550000	-25.4404540000	26.1637080000

C11	-8.1927580000	-26.3020380000	26.6112330000
C12	-5.8745950000	-25.9148520000	26.1848390000
C13	-7.8844470000	-27.5783560000	27.0905670000
C14	-5.5451180000	-27.1791970000	26.6713500000
C15	-6.5593670000	-28.0332620000	27.1468260000
O16	-6.1966770000	-29.2358510000	27.6521590000
C17	2.9850150000	-33.3740350000	28.0369680000
C18	3.1961060000	-33.6015400000	26.5431840000
O19	2.7617370000	-34.5924000000	25.9625360000
C20	1.5094210000	-33.6759070000	28.4034500000
C21	0.4951530000	-32.7812190000	27.7404140000
N22	-0.4692650000	-32.0917130000	28.4627130000
C23	0.2231630000	-32.4674640000	26.4283310000
C24	-1.2586860000	-31.4067400000	27.5769790000
N25	-0.8550500000	-31.6072060000	26.3376620000
N26	3.8821030000	-32.6092410000	25.9095970000
C27	4.2949790000	-32.6900940000	24.5159090000
C28	4.0885110000	-31.3425410000	23.7868140000
C29	2.6606950000	-30.9038600000	23.6579250000
N30	2.0052170000	-30.8320790000	22.4325580000
C31	1.7059950000	-30.5352000000	24.5751460000
C32	0.7221760000	-30.4403080000	22.6479320000
N33	0.5093200000	-30.2659910000	23.9421160000
C34	-7.0255360000	-37.1408960000	19.9820580000
C35	-5.7350280000	-36.9741550000	20.8040180000
C36	-5.7303630000	-35.7393840000	21.6492560000
N37	-6.7607340000	-35.4321400000	22.5238690000
C38	-4.8288170000	-34.7122040000	21.7863080000
C39	-6.4569770000	-34.2657510000	23.1391810000
N40	-5.2924190000	-33.8064160000	22.7170750000
Cu41	-1.6642510000	-30.2413430000	24.3058030000
Fe42	-4.3099870000	-32.0993080000	23.4578630000
C43	-1.4337780000	-32.9824750000	21.8113250000
C44	-3.4559430000	-34.0464440000	26.1012990000
C45	-7.1674180000	-31.1101260000	25.1018710000
C46	-5.4491600000	-30.5809080000	20.6231330000
N47	-5.9787600000	-31.0384620000	22.9614520000
C48	-1.6620180000	-33.5416110000	23.0710390000
C49	-0.7860170000	-34.4634970000	23.7189290000
C50	-1.3689060000	-34.7890430000	24.9250410000
C51	-2.6032070000	-34.0371970000	24.9994090000
C52	-0.7037180000	-35.5836300000	25.9498980000
O53	-1.1107800000	-35.8048320000	27.0865120000
N54	-5.1601400000	-32.4972420000	25.2631870000
C55	-4.6271610000	-33.3182260000	26.2210830000
C56	-5.4906400000	-33.3593180000	27.3888470000
C57	-6.5587920000	-32.5516780000	27.1009310000
C58	-6.3239550000	-32.0017010000	25.7679690000
C59	-5.2192210000	-34.1850740000	28.6104370000
N60	-2.7624550000	-33.3068430000	23.8603060000

C61	-7.0009270000	-30.6707290000	23.7948270000
C62	-7.9676310000	-29.8419550000	23.0890410000
C63	-7.5534590000	-29.7988800000	21.7789540000
C64	-6.2760540000	-30.5171600000	21.7358210000
C65	-9.1149180000	-29.1127190000	23.7182580000
C66	-8.1868980000	-29.1544930000	20.6361330000
C67	-9.4958120000	-28.8986660000	20.4892830000
N68	-3.5575610000	-31.7971950000	21.6055760000
C69	-4.1685070000	-31.1270620000	20.5866850000
C70	-3.2859690000	-31.0427720000	19.4262520000
C71	-2.1396810000	-31.6832430000	19.7861230000
C72	-2.3230010000	-32.1685560000	21.1353660000
C73	-3.6081370000	-30.3512000000	18.1379090000
C74	-7.8288340000	-32.4714160000	27.8903880000
O75	-8.2760150000	-31.0917400000	27.9615000000
C76	-8.9148340000	-33.3647800000	27.2639770000
H77	0.1688360000	-34.8064380000	23.3380780000
H78	-1.2481330000	-31.8317050000	19.1878870000
H79	-1.2233640000	-26.6914620000	23.7242490000
H80	0.0070160000	-27.6321130000	25.6817800000
H81	-0.2934690000	-26.1500970000	26.6058900000
H82	-8.5410070000	-24.7317540000	23.8754580000
H83	-6.6245230000	-23.3906020000	25.8548240000
H84	-8.3481390000	-23.6280840000	26.0994450000
H85	3.6369760000	-34.0707740000	28.5782590000
H86	1.3001730000	-34.7157290000	28.1274780000
H87	1.3967000000	-33.6084330000	29.4928040000
H88	3.7024050000	-33.4847760000	24.0579130000
H89	4.6730030000	-30.5714300000	24.3100740000
H90	4.5425300000	-31.4251730000	22.7903400000
H91	-7.8965670000	-37.2185780000	20.6426790000
H92	-5.5956930000	-37.8637120000	21.4359680000
H93	-4.8666530000	-36.9318200000	20.1367840000
H94	-5.8922270000	-33.9311750000	29.4356960000
H95	-4.1914900000	-34.0363620000	28.9617560000
H96	-5.3310150000	-35.2584110000	28.4079210000
H97	-10.0536080000	-29.6848890000	23.6897870000
H98	-9.2799990000	-28.1668150000	23.1899090000
H99	-8.9005780000	-28.8739780000	24.7651880000
H100	-2.7862460000	-30.4563100000	17.4224780000
H101	-3.7809940000	-29.2790850000	18.2963140000
H102	-4.5142860000	-30.7594300000	17.6716630000
H103	-7.6309880000	-32.8201640000	28.9131240000
H104	-8.5126360000	-34.3732410000	27.1204760000
H105	-9.1999060000	-32.9652370000	26.2856900000
H106	-9.8097930000	-33.4561350000	27.8942260000
H107	-0.5024810000	-33.2402500000	21.3156890000
H108	-5.8021630000	-30.1135370000	19.7112670000
H109	-7.5259410000	-28.8647690000	19.8208310000
H110	-10.2318560000	-29.1791550000	21.2358650000

H111	-9.8678930000	-28.3889620000	19.6055510000
H112	-8.0375880000	-30.7584300000	25.6400950000
H113	-3.1422420000	-34.6686130000	26.9304540000
H114	0.2872420000	-35.9733510000	25.6344850000
H115	-7.0832170000	-33.7827760000	23.8728520000
H116	-7.6095730000	-35.9753070000	22.6774200000
H117	-3.8816270000	-34.5602040000	21.2919810000
H118	-0.0526390000	-30.2648870000	21.9106390000
H119	2.4192160000	-31.0137650000	21.5301070000
H120	1.7943840000	-30.4782120000	25.6479660000
H121	4.2508700000	-31.8492970000	26.4640730000
H122	0.7258630000	-32.8240510000	25.5459960000
H123	-0.5801530000	-32.1608510000	29.4668350000
H124	-2.0846090000	-30.7735820000	27.8664610000
H125	-4.2824940000	-29.6337030000	25.9701630000
H126	-3.2393140000	-25.6634330000	27.0144330000
H127	-5.0811250000	-25.2951200000	25.7828220000
H128	-8.6739880000	-28.2356610000	27.4462570000
H129	-9.2313250000	-25.9795280000	26.5904140000
H130	-9.0513330000	-31.0492860000	28.5425050000
H131	3.2711960000	-32.3595050000	28.3144420000
H132	-1.4217220000	-25.2220670000	24.5787490000
H133	0.0891520000	-25.7122800000	24.1996020000
H134	-8.0230670000	-23.1344150000	23.7900810000
N135	-6.6192830000	-24.4989970000	23.2804390000
H136	5.2636620000	-32.9381870000	24.4990390000
H137	-7.1782210000	-36.2818300000	19.3191590000
H138	-6.9834370000	-38.0481530000	19.3686390000
H139	-4.7454930000	-25.0028100000	21.7066330000
C140	-5.5860260000	-25.6410160000	21.3980060000
C141	-6.8148070000	-25.3700310000	22.2387680000
O142	-7.8962140000	-25.9058990000	22.0123290000
C143	-5.1489940000	-27.1228870000	21.4740100000
C144	-3.8407510000	-27.3191800000	20.6987900000
C145	-5.0298930000	-27.6096310000	22.9232010000
H146	-5.7216760000	-24.0452980000	23.3662090000
H147	-5.8275560000	-25.3821180000	20.3598160000
H148	-5.9369030000	-27.7056540000	20.9889510000
H149	-3.0115980000	-26.7940350000	21.1875780000
H150	-3.9267870000	-26.9450210000	19.6690060000
H151	-3.5628550000	-28.3753910000	20.6583080000
H152	-5.9957940000	-27.5749990000	23.4366070000
H153	-4.3203900000	-26.9870870000	23.4860610000
H154	-4.6561680000	-28.6351170000	22.9828410000
H155	-2.6942950000	-29.4886700000	22.3799650000
C156	-11.0220450000	-37.0394970000	21.4210220000
C157	-10.4123990000	-36.6560860000	22.7498740000
O158	-9.2066220000	-36.7534480000	22.9904820000
N159	-11.2781580000	-36.1723140000	23.6713100000
C160	-10.8110350000	-35.7070430000	24.9628930000

H161	-10.3498020000	-37.7242770000	20.9016270000
H162	-11.1577570000	-36.1476330000	20.7983720000
H163	-12.2620660000	-36.1252400000	23.4525170000
H164	-10.0028900000	-34.9799800000	24.8361400000
H165	-11.6388260000	-35.2290500000	25.4912720000
H166	-11.9911780000	-37.5204700000	21.5567680000
H167	-10.4290620000	-36.5298540000	25.5672380000
O168	-1.7673760000	-29.3269620000	22.6091800000
O169	-3.5258150000	-30.7274780000	24.1963330000
H170	-6.9773380000	-29.8128680000	27.7982010000

O_B (quintet, +1)

C1	-0.8188620000	-26.0150660000	24.4889300000
C2	-0.7381750000	-26.9302520000	25.7338940000
C3	-2.0800300000	-27.5216220000	26.0376070000
N4	-2.4875880000	-28.7931860000	25.6500260000
C5	-3.1711670000	-26.8562090000	26.5199850000
C6	-3.7975010000	-28.8717210000	25.8642940000
N7	-4.2580830000	-27.7086790000	26.3959870000
C8	-7.7228740000	-24.0381160000	24.0959550000
C9	-7.4563690000	-24.0587020000	25.6110590000
C10	-7.1915530000	-25.4508840000	26.1294990000
C11	-8.2076880000	-26.2898810000	26.6039160000
C12	-5.9012640000	-25.9704840000	26.0661130000
C13	-7.9299180000	-27.5876580000	27.0320800000
C14	-5.6085360000	-27.2624440000	26.4984950000
C15	-6.6206100000	-28.0903840000	27.0072980000
O16	-6.2754830000	-29.3151740000	27.4650940000
C17	2.9851990000	-33.3739850000	28.0369550000
C18	3.1581840000	-33.5653570000	26.5312710000
O19	2.6302230000	-34.4929890000	25.9243530000
C20	1.5071120000	-33.6607250000	28.4017090000
C21	0.5264940000	-32.7216570000	27.7493570000
N22	-0.4120290000	-32.0191920000	28.4869140000
C23	0.2977010000	-32.3278580000	26.4531100000
C24	-1.1482900000	-31.2435840000	27.6563380000
N25	-0.7258270000	-31.3942410000	26.4132270000
N26	3.8902470000	-32.5937420000	25.9123670000
C27	4.2947060000	-32.6899800000	24.5164340000
C28	4.0186490000	-31.3778020000	23.7411900000
C29	2.5618490000	-31.0618460000	23.5482030000
N30	1.8748990000	-31.3455810000	22.3710540000
C31	1.6031070000	-30.5330430000	24.3753600000
C32	0.5735810000	-30.9939580000	22.5205540000
N33	0.3699220000	-30.5114290000	23.7377070000
C34	-7.0503710000	-37.4911460000	20.2679210000
C35	-5.7889040000	-37.2641940000	21.1188600000
C36	-5.7819330000	-35.9477880000	21.8305610000

N37	-6.8035350000	-35.5367430000	22.6701320000
C38	-4.8715440000	-34.9204250000	21.8516040000
C39	-6.4838110000	-34.3129080000	23.1532690000
N40	-5.3164940000	-33.9098360000	22.6803360000
Cu41	-1.5348690000	-30.3854030000	24.7529660000
Fe42	-4.1644440000	-31.9828000000	23.2206380000
C43	-1.5179640000	-33.4715000000	21.5512070000
C44	-3.4348220000	-33.8406560000	26.0252340000
C45	-7.1439120000	-30.9737420000	24.7436120000
C46	-5.3414780000	-30.8024160000	20.2326410000
N47	-5.8899620000	-30.9988250000	22.6237950000
C48	-1.6810030000	-33.8165470000	22.9067780000
C49	-0.8194110000	-34.6750560000	23.6618080000
C50	-1.3689800000	-34.7890320000	24.9250200000
C51	-2.5673720000	-33.9798970000	24.9340740000
C52	-0.7171700000	-35.4837150000	26.0308690000
O53	-1.0972130000	-35.4953350000	27.1970810000
N54	-5.1058930000	-32.2747340000	25.1110880000
C55	-4.6171620000	-33.1079790000	26.0852110000
C56	-5.5648260000	-33.1807250000	27.1818870000
C57	-6.6444960000	-32.4085500000	26.8228820000
C58	-6.3338770000	-31.8311690000	25.5124360000
C59	-5.3786110000	-34.0218770000	28.4097270000
N60	-2.7185550000	-33.4163860000	23.7071420000
C61	-6.9362880000	-30.5889700000	23.4158250000
C62	-7.8803750000	-29.8140470000	22.6146410000
C63	-7.4201690000	-29.8584670000	21.3169560000
C64	-6.1569450000	-30.5931680000	21.3461760000
C65	-9.0964320000	-29.1080820000	23.1227460000
C66	-8.0455090000	-29.3636760000	20.0903220000
C67	-8.7179220000	-28.2113670000	19.9531570000
N68	-3.5358540000	-32.1193350000	21.2314780000
C69	-4.1235280000	-31.4897540000	20.1802620000
C70	-3.2996230000	-31.6534790000	18.9837670000
C71	-2.2246380000	-32.4002570000	19.3678130000
C72	-2.3739640000	-32.6931110000	20.7792110000
C73	-3.6062000000	-31.0774750000	17.6372830000
C74	-7.9178830000	-32.3355790000	27.6247330000
O75	-8.4462390000	-30.9867800000	27.5506740000
C76	-8.9756580000	-33.3594670000	27.1763900000
H77	0.0929170000	-35.1381840000	23.3052520000
H78	-1.3994420000	-32.7296140000	18.7476020000
H79	-1.2251290000	-26.6129340000	23.6593360000
H80	-0.0224230000	-27.7382680000	25.5402930000
H81	-0.3649520000	-26.3674530000	26.5985300000
H82	-8.5324590000	-24.7379860000	23.8720610000
H83	-6.6103960000	-23.3997820000	25.8505330000
H84	-8.3345070000	-23.6268270000	26.1068820000
H85	3.6372050000	-34.0809970000	28.5620710000
H86	1.2662250000	-34.6858710000	28.1037330000

H87	1.3878160000	-33.6072390000	29.4902060000
H88	3.7183700000	-33.5142590000	24.0889780000
H89	4.5021750000	-30.5432010000	24.2644440000
H90	4.5156210000	-31.4444220000	22.7645110000
H91	-7.9433980000	-37.4858440000	20.9012180000
H92	-5.6953310000	-38.0824470000	21.8473510000
H93	-4.8939280000	-37.3096350000	20.4878640000
H94	-6.0239440000	-33.6976010000	29.2323420000
H95	-4.3438070000	-33.9811360000	28.7658940000
H96	-5.6052370000	-35.0776940000	28.2122970000
H97	-9.8694670000	-29.0716170000	22.3474060000
H98	-8.8546500000	-28.0682390000	23.3786790000
H99	-9.5157390000	-29.5891840000	24.0127740000
H100	-2.8415440000	-31.3570920000	16.9065820000
H101	-3.6486520000	-29.9816470000	17.6791440000
H102	-4.5762320000	-31.4248330000	17.2596280000
H103	-7.6635980000	-32.5407870000	28.6732030000
H104	-8.5127410000	-34.3475690000	27.0853520000
H105	-9.3727520000	-33.0725630000	26.1973690000
H106	-9.8098350000	-33.4561500000	27.8942640000
H107	-0.6467680000	-33.8856510000	21.0508980000
H108	-5.6850030000	-30.3695410000	19.2994520000
H109	-7.9398930000	-30.0137830000	19.2209800000
H110	-8.7868180000	-27.4844210000	20.7545890000
H111	-9.1654490000	-27.9481660000	18.9981000000
H112	-8.0485960000	-30.6183050000	25.2200200000
H113	-3.1511650000	-34.4103240000	26.9018250000
H114	0.2287740000	-35.9894260000	25.7510970000
H115	-7.1076960000	-33.7504230000	23.8308010000
H116	-7.6648610000	-36.0493810000	22.8645650000
H117	-3.9291780000	-34.8450550000	21.3329890000
H118	-0.1827120000	-31.0370430000	21.7498170000
H119	2.2841980000	-31.7135110000	21.5236680000
H120	1.7077340000	-30.2056900000	25.3975830000
H121	4.3766650000	-31.9231150000	26.4923020000
H122	0.7778800000	-32.6677440000	25.5532930000
H123	-0.5801860000	-32.1609150000	29.4666790000
H124	-1.9534860000	-30.5962540000	27.9699970000
H125	-4.4186170000	-29.7190950000	25.6211640000
H126	-3.2750530000	-25.8624940000	26.9247890000
H127	-5.1070070000	-25.3685280000	25.6395870000
H128	-8.7261830000	-28.2229100000	27.4086170000
H129	-9.2314670000	-25.9260220000	26.6448920000
H130	-9.2124420000	-30.9245510000	28.1432630000
H131	3.2712030000	-32.3595430000	28.3144110000
H132	-1.4218470000	-25.2220820000	24.5788000000
H133	0.0891240000	-25.7121790000	24.1996120000
H134	-8.0232390000	-23.1343840000	23.7901020000
N135	-6.6067050000	-24.4733430000	23.2795870000
H136	5.2637210000	-32.9382930000	24.4987430000

H137	-7.1588160000	-36.7032240000	19.5140150000
H138	-7.0019550000	-38.4568860000	19.7530120000
H139	-4.7317160000	-25.0163190000	21.6948390000
C140	-5.5860000000	-25.6409850000	21.3980450000
C141	-6.7906460000	-25.3816410000	22.2758880000
O142	-7.8489390000	-25.9932610000	22.1340270000
C143	-5.1960740000	-27.1337470000	21.4694600000
C144	-3.9417490000	-27.3818840000	20.6289980000
C145	-5.0299300000	-27.6096290000	22.9231800000
H146	-5.7655830000	-23.9137650000	23.2702180000
H147	-5.8480250000	-25.3770120000	20.3666650000
H148	-6.0258450000	-27.6939010000	21.0292720000
H149	-3.0816650000	-26.8319910000	21.0404820000
H150	-4.0833930000	-27.0374770000	19.5964660000
H151	-3.6767860000	-28.4415400000	20.6048430000
H152	-5.9737430000	-27.5586190000	23.4731480000
H153	-4.2931030000	-26.9893570000	23.4551830000
H154	-4.6693220000	-28.6422540000	22.9571800000
H155	-2.3548740000	-30.0518090000	22.1197170000
C156	-11.0220210000	-37.0395550000	21.4210410000
C157	-10.4231850000	-36.6938970000	22.7656570000
O158	-9.2317610000	-36.8653600000	23.0418210000
N159	-11.2797030000	-36.1631130000	23.6687740000
C160	-10.8109960000	-35.7070270000	24.9628940000
H161	-10.3545870000	-37.7166820000	20.8869970000
H162	-11.1611650000	-36.1333810000	20.8207150000
H163	-12.2520420000	-36.0466920000	23.4238570000
H164	-10.0014740000	-34.9799270000	24.8398820000
H165	-11.6382990000	-35.2303540000	25.4929430000
H166	-11.9911580000	-37.5204010000	21.5567610000
H167	-10.4290950000	-36.5298690000	25.5672470000
O168	-1.7975760000	-29.6769580000	21.3923570000
O169	-3.0193030000	-30.6732350000	23.6111090000
H170	-1.6299050000	-28.7676030000	21.6800290000
H171	-7.0836250000	-29.8590680000	27.6077230000

O_{BR} (sextet, 0)

C1	-0.8188620000	-26.0150660000	24.4889300000
C2	-0.7212910000	-26.8187980000	25.8090520000
C3	-2.0572040000	-27.3916760000	26.1547280000
N4	-2.4305390000	-28.6572270000	25.7362940000
C5	-3.1555480000	-26.7480560000	26.6535680000
C6	-3.7284560000	-28.7700810000	25.9499260000
N7	-4.2255930000	-27.6269230000	26.5178280000
C8	-7.7228740000	-24.0381160000	24.0959550000
C9	-7.4753910000	-24.0584660000	25.6138440000
C10	-7.2036140000	-25.4470690000	26.1375180000
C11	-8.2194630000	-26.3205720000	26.5423490000

C12	-5.8969030000	-25.9282950000	26.1388530000
C13	-7.9236470000	-27.6154960000	26.9715400000
C14	-5.5830240000	-27.2143710000	26.5710700000
C15	-6.5994720000	-28.0764900000	27.0175130000
O16	-6.2422550000	-29.2940600000	27.4823300000
C17	2.9851990000	-33.3739850000	28.0369550000
C18	3.2023480000	-33.6008910000	26.5438750000
O19	2.7791650000	-34.5977550000	25.9659670000
C20	1.5106280000	-33.6614910000	28.3982300000
C21	0.5287800000	-32.7293280000	27.7462430000
N22	-0.4444130000	-32.0630800000	28.4729540000
C23	0.2982540000	-32.3537750000	26.4430500000
C24	-1.2080550000	-31.3348120000	27.6094360000
N25	-0.7720010000	-31.4833450000	26.3741380000
N26	3.8755130000	-32.6005820000	25.9064120000
C27	4.2947060000	-32.6899800000	24.5164340000
C28	4.0478320000	-31.3667760000	23.7563100000
C29	2.6051760000	-31.0012190000	23.5793570000
N30	1.8726630000	-31.3337900000	22.4452110000
C31	1.7001910000	-30.3592070000	24.3889120000
C32	0.5965080000	-30.8867450000	22.6029570000
N33	0.4600800000	-30.2976130000	23.7806720000
C34	-7.0401990000	-37.8304860000	20.4940150000
C35	-5.7759890000	-37.5175210000	21.3147300000
C36	-5.8202660000	-36.1856800000	21.9954930000
N37	-6.8163100000	-35.8247150000	22.8891490000
C38	-4.9884190000	-35.0928230000	21.9353430000
C39	-6.5506490000	-34.5586090000	23.3132270000
N40	-5.4506050000	-34.0888790000	22.7588480000
Cu41	-1.4833770000	-30.2867890000	24.6993240000
Fe42	-4.2087150000	-31.9571130000	23.2769530000
C43	-1.6205700000	-33.5023990000	21.5427930000
C44	-3.4236190000	-33.8384210000	26.0621380000
C45	-7.2111920000	-31.0521030000	24.8090840000
C46	-5.4838600000	-30.8670900000	20.2737920000
N47	-6.0187520000	-31.0787560000	22.6619950000
C48	-1.7447990000	-33.8322720000	22.9074800000
C49	-0.8535640000	-34.6756740000	23.6464390000
C50	-1.3689800000	-34.7890320000	24.9250200000
C51	-2.5773340000	-33.9861870000	24.9531270000
C52	-0.6973850000	-35.4904060000	26.0078200000
O53	-1.0741770000	-35.5684700000	27.1748030000
N54	-5.1415220000	-32.3171120000	25.1597610000
C55	-4.6147040000	-33.1161190000	26.1384260000
C56	-5.5329680000	-33.1709670000	27.2634320000
C57	-6.6313850000	-32.4240720000	26.9080680000
C58	-6.3634630000	-31.8817080000	25.5724850000
C59	-5.2980890000	-33.9673270000	28.5129720000
N60	-2.7617740000	-33.4307060000	23.7294600000
C61	-7.0446290000	-30.6724800000	23.4712680000

C62	-7.9992700000	-29.8844890000	22.6905840000
C63	-7.5511810000	-29.9088310000	21.3887900000
C64	-6.2915570000	-30.6586930000	21.3965990000
C65	-9.2094840000	-29.1865980000	23.2254890000
C66	-8.1829810000	-29.3889260000	20.1775300000
C67	-8.8518340000	-28.2323470000	20.0571350000
N68	-3.6674070000	-32.1959270000	21.2403010000
C69	-4.2682790000	-31.5623460000	20.2023620000
C70	-3.4562550000	-31.7141380000	18.9931340000
C71	-2.3735040000	-32.4555440000	19.3600880000
C72	-2.5066830000	-32.7520710000	20.7747750000
C73	-3.7751350000	-31.1230930000	17.6551650000
C74	-7.9004980000	-32.3589550000	27.7051620000
O75	-8.4164370000	-31.0008430000	27.6755680000
C76	-8.9626750000	-33.3488810000	27.1978510000
H77	0.0534600000	-35.1336170000	23.2689250000
H78	-1.5481450000	-32.7681010000	18.7307210000
H79	-1.2291880000	-26.6674540000	23.7080020000
H80	-0.0086290000	-27.6421430000	25.6841050000
H81	-0.3486420000	-26.1712860000	26.6138820000
H82	-8.5346730000	-24.7347770000	23.8676070000
H83	-6.6385100000	-23.3913710000	25.8650400000
H84	-8.3647550000	-23.6321260000	26.0960520000
H85	3.6331990000	-34.0736540000	28.5787660000
H86	1.2752960000	-34.6906170000	28.1049440000
H87	1.3957870000	-33.6083340000	29.4882980000
H88	3.7156700000	-33.5041050000	24.0734180000
H89	4.5619230000	-30.5525910000	24.2848070000
H90	4.5395980000	-31.4396420000	22.7766460000
H91	-7.9194670000	-37.8675730000	21.1455630000
H92	-5.6236010000	-38.3175130000	22.0546280000
H93	-4.8944060000	-37.5301090000	20.6633210000
H94	-5.9452160000	-33.6440960000	29.3356380000
H95	-4.2602550000	-33.8703690000	28.8506740000
H96	-5.4800240000	-35.0386010000	28.3528670000
H97	-9.9763630000	-29.0941720000	22.4481120000
H98	-8.9591050000	-28.1674460000	23.5497890000
H99	-9.6427700000	-29.7122730000	24.0844960000
H100	-3.0173990000	-31.3953150000	16.9132730000
H101	-3.8154940000	-30.0272670000	17.7091690000
H102	-4.7501990000	-31.4626520000	17.2813780000
H103	-7.6635470000	-32.6082340000	28.7485620000
H104	-8.5093090000	-34.3389050000	27.0817390000
H105	-9.3328210000	-33.0213280000	26.2207010000
H106	-9.8098350000	-33.4561500000	27.8942640000
H107	-0.7488450000	-33.9028090000	21.0308670000
H108	-5.8303460000	-30.4210890000	19.3469520000
H109	-8.0848450000	-30.0258660000	19.2967070000
H110	-8.9082890000	-27.5094700000	20.8628370000
H111	-9.3031030000	-27.9551110000	19.1070900000

H112	-8.1050810000	-30.6983200000	25.3091490000
H113	-3.1155840000	-34.3918880000	26.9417040000
H114	0.2675860000	-35.9532890000	25.7104230000
H115	-7.1653280000	-34.0208320000	24.0216600000
H116	-7.6400910000	-36.3745880000	23.1209640000
H117	-4.0827600000	-34.9633870000	21.3619960000
H118	-0.1941530000	-30.9463940000	21.8639430000
H119	2.2293320000	-31.8034170000	21.6256690000
H120	1.8499010000	-29.9691680000	25.3848250000
H121	4.2289910000	-31.8305030000	26.4566210000
H122	0.8086450000	-32.6717800000	25.5496770000
H123	-0.5801860000	-32.1609150000	29.4666790000
H124	-2.0425260000	-30.7162040000	27.9051000000
H125	-4.3301400000	-29.6205790000	25.6683980000
H126	-3.2835170000	-25.7659930000	27.0809800000
H127	-5.0980830000	-25.3002360000	25.7606540000
H128	-8.7185970000	-28.2842240000	27.2894040000
H129	-9.2556790000	-25.9899620000	26.5247330000
H130	-9.1601290000	-30.9422950000	28.2958180000
H131	3.2712030000	-32.3595430000	28.3144110000
H132	-1.4218470000	-25.2220820000	24.5788000000
H133	0.0891240000	-25.7121790000	24.1996120000
H134	-8.0232390000	-23.1343840000	23.7901020000
N135	-6.6070710000	-24.4898910000	23.2914710000
H136	5.2637210000	-32.9382930000	24.4987430000
H137	-7.2084250000	-37.0581130000	19.7345520000
H138	-6.9484290000	-38.7987060000	19.9874740000
H139	-4.7239030000	-25.0367620000	21.7152600000
C140	-5.5860000000	-25.6409850000	21.3980450000
C141	-6.8067500000	-25.3392810000	22.2371600000
O142	-7.9054670000	-25.8423840000	22.0080830000
C143	-5.2178290000	-27.1421550000	21.4722230000
C144	-3.9733120000	-27.4108960000	20.6208500000
C145	-5.0299300000	-27.6096290000	22.9231800000
H146	-5.7098590000	-24.0374700000	23.3878500000
H147	-5.8173470000	-25.3700220000	20.3607370000
H148	-6.0604710000	-27.6957800000	21.0454060000
H149	-3.1129450000	-26.8389170000	20.9999450000
H150	-4.1340030000	-27.1101760000	19.5766680000
H151	-3.6905430000	-28.4664500000	20.6429860000
H152	-5.9667490000	-27.5552480000	23.4876290000
H153	-4.2892850000	-26.9824370000	23.4418890000
H154	-4.6603980000	-28.6399120000	22.9662110000
H155	-2.4311380000	-30.1121830000	22.1644860000
C156	-11.0220210000	-37.0395550000	21.4210410000
C157	-10.4347670000	-36.7956500000	22.7986640000
O158	-9.3140250000	-37.1742550000	23.1425650000
N159	-11.2635710000	-36.1497920000	23.6589320000
C160	-10.8109960000	-35.7070270000	24.9628940000
H161	-10.3614560000	-37.6883390000	20.8462960000

H162	-11.1661230000	-36.0968210000	20.8814160000
H163	-12.1381660000	-35.7882390000	23.3065050000
H164	-10.0085080000	-34.9642830000	24.8730200000
H165	-11.6511270000	-35.2486310000	25.4903110000
H166	-11.9911580000	-37.5204010000	21.5567610000
H167	-10.4290950000	-36.5298690000	25.5672470000
O168	-1.8675350000	-29.7396390000	21.4202210000
O169	-3.1373330000	-30.5807440000	23.6000920000
H170	-1.5427350000	-28.9172740000	21.8180140000
H171	-7.0365680000	-29.8611830000	27.6014630000

E (sextet, +1)

C1	-0.8189090000	-26.0151940000	24.4889320000
C2	-0.7356240000	-26.8813840000	25.7651230000
C3	-2.0789740000	-27.4454270000	26.1025220000
N4	-2.4645780000	-28.7344250000	25.7642250000
C5	-3.1670940000	-26.7752840000	26.5896090000
C6	-3.7603570000	-28.8284110000	26.0352570000
N7	-4.2419380000	-27.6498700000	26.5299220000
C8	-7.7228630000	-24.0381220000	24.0959470000
C9	-7.4607730000	-24.0508650000	25.6119290000
C10	-7.1995850000	-25.4388730000	26.1414280000
C11	-8.2242780000	-26.2974620000	26.5549240000
C12	-5.8989270000	-25.9333520000	26.1457410000
C13	-7.9446690000	-27.5955390000	26.9821530000
C14	-5.6006720000	-27.2232420000	26.5846810000
C15	-6.6281690000	-28.0756490000	27.0210630000
O16	-6.2926990000	-29.3127190000	27.4615880000
C17	2.9851550000	-33.3739620000	28.0369420000
C18	3.1612830000	-33.5758510000	26.5332450000
O19	2.6517150000	-34.5189440000	25.9337390000
C20	1.5185820000	-33.6701550000	28.4208070000
C21	0.5202560000	-32.7642970000	27.7537800000
N22	-0.4565660000	-32.0958060000	28.4712790000
C23	0.2759100000	-32.4153370000	26.4455450000
C24	-1.2317820000	-31.3877720000	27.6082780000
N25	-0.8037490000	-31.5561940000	26.3713050000
N26	3.8844300000	-32.6015170000	25.9104180000
C27	4.2946800000	-32.6899640000	24.5164050000
C28	4.0572990000	-31.3619120000	23.7622800000
C29	2.6140950000	-31.0104710000	23.5711040000
N30	1.9154670000	-31.3147810000	22.4098000000
C31	1.6768760000	-30.4307790000	24.3894290000
C32	0.6247560000	-30.9165900000	22.5571780000
N33	0.4457980000	-30.3807560000	23.7560880000
C34	-7.0125890000	-37.7797130000	20.3946970000
C35	-5.7490660000	-37.4918370000	21.2244450000
C36	-5.7718780000	-36.1527140000	21.8930070000

N37	-6.8069550000	-35.7428860000	22.7174190000
C38	-4.8888900000	-35.1017490000	21.8831670000
C39	-6.5260490000	-34.4969220000	23.1611460000
N40	-5.3682880000	-34.0796090000	22.6768820000
Cu41	-1.3964760000	-30.2976880000	24.7636510000
Fe42	-4.2813770000	-32.1575510000	23.1784970000
C43	-1.5436410000	-33.5093120000	21.5391010000
C44	-3.4480870000	-33.8713070000	26.0191780000
C45	-7.1963760000	-31.0510410000	24.7374040000
C46	-5.4162700000	-30.9082300000	20.2106040000
N47	-5.9696340000	-31.1117800000	22.6031340000
C48	-1.6909110000	-33.8288080000	22.9010600000
C49	-0.8181610000	-34.6689950000	23.6637570000
C50	-1.3690170000	-34.7890590000	24.9250090000
C51	-2.5789840000	-33.9997480000	24.9295690000
C52	-0.7190760000	-35.4866560000	26.0351470000
O53	-1.1445290000	-35.5610470000	27.1812040000
N54	-5.1581660000	-32.3592870000	25.0872710000
C55	-4.6405740000	-33.1556280000	26.0774460000
C56	-5.5612950000	-33.1924290000	27.1960370000
C57	-6.6499890000	-32.4323200000	26.8361320000
C58	-6.3753550000	-31.8977240000	25.5036770000
C59	-5.3394270000	-33.9832310000	28.4509640000
N60	-2.7324940000	-33.4319690000	23.7007350000
C61	-7.0023200000	-30.6767730000	23.4037060000
C62	-7.9218680000	-29.8710650000	22.6123180000
C63	-7.4659850000	-29.9180320000	21.3092040000
C64	-6.2287700000	-30.6890740000	21.3252980000
C65	-9.1122040000	-29.1276720000	23.1280500000
C66	-8.0777000000	-29.3855170000	20.0921820000
C67	-8.7266520000	-28.2173270000	19.9758760000
N68	-3.6063190000	-32.2301180000	21.2064610000
C69	-4.2021830000	-31.6022910000	20.1547740000
C70	-3.3750650000	-31.7526760000	18.9611110000
C71	-2.2872270000	-32.4823720000	19.3465860000
C72	-2.4309050000	-32.7764170000	20.7560560000
C73	-3.6814340000	-31.1729160000	17.6159930000
C74	-7.9158430000	-32.3490980000	27.6439670000
O75	-8.4494840000	-31.0034750000	27.5506760000
C76	-8.9607250000	-33.3831140000	27.1967720000
H77	0.1044500000	-35.1151770000	23.3124030000
H78	-1.4507890000	-32.7871670000	18.7291180000
H79	-1.2258310000	-26.6345920000	23.6766150000
H80	-0.0404690000	-27.7125300000	25.5970360000
H81	-0.3446010000	-26.2880330000	26.6013010000
H82	-8.5332380000	-24.7373310000	23.8731920000
H83	-6.6159360000	-23.3907950000	25.8517190000
H84	-8.3410280000	-23.6137640000	26.0996380000
H85	3.6480300000	-34.0738750000	28.5594440000
H86	1.2925700000	-34.7073210000	28.1510470000

H87	1.4108100000	-33.5933270000	29.5092740000
H88	3.7121840000	-33.5008810000	24.0731910000
H89	4.5605300000	-30.5494120000	24.3020100000
H90	4.5586430000	-31.4265980000	22.7875250000
H91	-7.9008560000	-37.7813150000	21.0352340000
H92	-5.6261210000	-38.2819480000	21.9788800000
H93	-4.8595830000	-37.5344350000	20.5858520000
H94	-5.9759970000	-33.6395680000	29.2721080000
H95	-4.2993250000	-33.9090390000	28.7855910000
H96	-5.5486910000	-35.0497280000	28.2969670000
H97	-9.8996430000	-29.0903070000	22.3673230000
H98	-8.8427360000	-28.0882000000	23.3568310000
H99	-9.5235640000	-29.5801750000	24.0363560000
H100	-2.9168340000	-31.4514560000	16.8849260000
H101	-3.7210810000	-30.0770890000	17.6616440000
H102	-4.6518120000	-31.5174030000	17.2369160000
H103	-7.6614970000	-32.5424610000	28.6940900000
H104	-8.4952320000	-34.3729520000	27.1472230000
H105	-9.3311480000	-33.1255150000	26.1997320000
H106	-9.8097910000	-33.4561000000	27.8942470000
H107	-0.6603320000	-33.9029430000	21.0440830000
H108	-5.7523810000	-30.4641820000	19.2801230000
H109	-7.9832630000	-30.0201760000	19.2105750000
H110	-8.7834270000	-27.5021000000	20.7887900000
H111	-9.1659470000	-27.9278950000	19.0246810000
H112	-8.0862420000	-30.6769370000	25.2272470000
H113	-3.1512400000	-34.4271550000	26.9001060000
H114	0.2598010000	-35.9361720000	25.7735120000
H115	-7.1632850000	-33.9293520000	23.8218610000
H116	-7.6655770000	-36.2630230000	22.9114910000
H117	-3.9457010000	-35.0124660000	21.3679870000
H118	-0.1403560000	-30.9746460000	21.7938590000
H119	2.3103650000	-31.7211280000	21.5735610000
H120	1.7945300000	-30.0814930000	25.4038490000
H121	4.3424130000	-31.9076390000	26.4859350000
H122	0.7844650000	-32.7416760000	25.5552440000
H123	-0.5801570000	-32.1608660000	29.4666730000
H124	-2.0630350000	-30.7652260000	27.9041880000
H125	-4.3746450000	-29.7039570000	25.8928180000
H126	-3.2737690000	-25.7716460000	26.9686300000
H127	-5.0976370000	-25.3112010000	25.7639810000
H128	-8.7487980000	-28.2508830000	27.3034780000
H129	-9.2554000000	-25.9531560000	26.5433580000
H130	-9.2133680000	-30.9328120000	28.1447910000
H131	3.2712140000	-32.3595430000	28.3144150000
H132	-1.4218280000	-25.2221490000	24.5787910000
H133	0.0891170000	-25.7121240000	24.1996730000
H134	-8.0232220000	-23.1343920000	23.7901120000
N135	-6.6067150000	-24.4755920000	23.2810130000
H136	5.2636750000	-32.9382860000	24.4987600000

H137	-7.1502020000	-37.0189510000	19.6180360000
H138	-6.9408380000	-38.7588460000	19.9091280000
H139	-4.7295430000	-25.0193580000	21.6945300000
C140	-5.5860060000	-25.6409930000	21.3980540000
C141	-6.7919060000	-25.3784560000	22.2737710000
O142	-7.8509470000	-25.9887540000	22.1251490000
C143	-5.2060290000	-27.1347520000	21.4711820000
C144	-3.9582020000	-27.4079970000	20.6279600000
C145	-5.0299250000	-27.6096290000	22.9231680000
H146	-5.7619980000	-23.9211350000	23.2774410000
H147	-5.8452900000	-25.3781780000	20.3658370000
H148	-6.0469120000	-27.6902920000	21.0454010000
H149	-3.0957530000	-26.8459570000	21.0156500000
H150	-4.1102280000	-27.0979100000	19.5864600000
H151	-3.6872150000	-28.4673100000	20.6352250000
H152	-5.9317390000	-27.4570880000	23.5222240000
H153	-4.1952000000	-27.0946540000	23.4190370000
H154	-4.8176400000	-28.6814900000	22.9168550000
H155	-2.3580700000	-30.1665080000	22.0817340000
C156	-11.0220230000	-37.0394780000	21.4210240000
C157	-10.4306720000	-36.7313960000	22.7792630000
O158	-9.2596920000	-36.9776270000	23.0854990000
N159	-11.2770860000	-36.1576610000	23.6657850000
C160	-10.8110120000	-35.7070310000	24.9628930000
H161	-10.3567470000	-37.7064930000	20.8723240000
H162	-11.1637720000	-36.1192440000	20.8431840000
H163	-12.2287630000	-35.9652100000	23.3888910000
H164	-10.0025940000	-34.9763490000	24.8475990000
H165	-11.6407430000	-35.2343790000	25.4929500000
H166	-11.9911060000	-37.5204580000	21.5567840000
H167	-10.4290510000	-36.5298500000	25.5672420000
O168	-1.8363400000	-29.8028220000	21.3242890000
O169	-3.1558470000	-30.6069450000	23.5839120000
H170	-1.4850680000	-28.9708840000	21.6768740000
H171	-3.6960990000	-29.8154560000	23.7336500000
H172	-7.1031260000	-29.8566060000	27.5875680000

$\mathbf{E_R}$ (quintet, 0)

C1	-0.8189090000	-26.0151940000	24.4889320000
C2	-0.7288270000	-26.8472960000	25.7898200000
C3	-2.0695140000	-27.4027400000	26.1526580000
N4	-2.4415420000	-28.6994800000	25.8383080000
C5	-3.1626000000	-26.7297570000	26.6249160000
C6	-3.7354710000	-28.8009430000	26.0966430000
N7	-4.2311450000	-27.6168060000	26.5773180000
C8	-7.7228630000	-24.0381220000	24.0959470000
C9	-7.4752180000	-24.0549980000	25.6137600000
C10	-7.2086400000	-25.4405520000	26.1497460000

C11	-8.2279620000	-26.3086010000	26.5579660000
C12	-5.9022780000	-25.9222010000	26.1679570000
C13	-7.9356860000	-27.5974390000	27.0052970000
C14	-5.5908530000	-27.2027110000	26.6213870000
C15	-6.6125290000	-28.0592500000	27.0692200000
O16	-6.2633040000	-29.2718300000	27.5502740000
C17	2.9851550000	-33.3739620000	28.0369420000
C18	3.2051120000	-33.6020390000	26.5442310000
O19	2.7927610000	-34.6041950000	25.9688340000
C20	1.5145150000	-33.6676060000	28.4021630000
C21	0.5360190000	-32.7338330000	27.7513600000
N22	-0.4369470000	-32.0652470000	28.4750000000
C23	0.3160530000	-32.3525700000	26.4487270000
C24	-1.1932880000	-31.3313180000	27.6125980000
N25	-0.7495290000	-31.4779620000	26.3800410000
N26	3.8727640000	-32.5983580000	25.9053270000
C27	4.2946800000	-32.6899640000	24.5164050000
C28	4.0573980000	-31.3682900000	23.7530170000
C29	2.6177500000	-31.0032750000	23.5692960000
N30	1.8757740000	-31.3822140000	22.4570580000
C31	1.7212910000	-30.3299540000	24.3614040000
C32	0.6001820000	-30.9325730000	22.6094170000
N33	0.4754340000	-30.2928520000	23.7628630000
C34	-7.0152480000	-37.9277190000	20.6139260000
C35	-5.7770160000	-37.6128270000	21.4728410000
C36	-5.8270620000	-36.2664070000	22.1237620000
N37	-6.8294230000	-35.8845750000	23.0028760000
C38	-4.9972100000	-35.1745990000	22.0396120000
C39	-6.5709270000	-34.6079060000	23.3961030000
N40	-5.4684270000	-34.1528630000	22.8350520000
Cu41	-1.4216640000	-30.3244380000	24.7489380000
Fe42	-4.3005750000	-32.1054990000	23.2229230000
C43	-1.6601000000	-33.5708390000	21.5109270000
C44	-3.3913280000	-33.7662160000	26.0726200000
C45	-7.2177970000	-31.0380460000	24.7999380000
C46	-5.5177420000	-30.9163250000	20.2437040000
N47	-6.0482300000	-31.0951920000	22.6368080000
C48	-1.7534630000	-33.8625070000	22.8922460000
C49	-0.8749600000	-34.7210690000	23.6319120000
C50	-1.3690170000	-34.7890590000	24.9250090000
C51	-2.5514390000	-33.9440420000	24.9575020000
C52	-0.6959330000	-35.4878880000	26.0066510000
O53	-1.0369630000	-35.5167960000	27.1875990000
N54	-5.1220740000	-32.2489700000	25.1816390000
C55	-4.5873540000	-33.0446210000	26.1571920000
C56	-5.5127350000	-33.1203650000	27.2784650000
C57	-6.6265480000	-32.3972050000	26.9156300000
C58	-6.3583460000	-31.8461060000	25.5803260000
C59	-5.2706970000	-33.9121970000	28.5299020000
N60	-2.7341130000	-33.4055330000	23.7272260000

C61	-7.0640460000	-30.6744380000	23.4515210000
C62	-8.0154040000	-29.8803690000	22.6711160000
C63	-7.5712280000	-29.9113180000	21.3654190000
C64	-6.3216280000	-30.6814380000	21.3699130000
C65	-9.2156460000	-29.1654260000	23.2068520000
C66	-8.1981510000	-29.3782180000	20.1579020000
C67	-8.8736230000	-28.2239780000	20.0447500000
N68	-3.7160660000	-32.2789990000	21.1957420000
C69	-4.3096040000	-31.6318130000	20.1638620000
C70	-3.4960660000	-31.7834720000	18.9507960000
C71	-2.4189870000	-32.5354000000	19.3149360000
C72	-2.5553630000	-32.8341290000	20.7330110000
C73	-3.8047230000	-31.1808410000	17.6154560000
C74	-7.8985850000	-32.3505060000	27.7097210000
O75	-8.4217720000	-30.9922790000	27.6844410000
C76	-8.9588540000	-33.3424870000	27.2004590000
H77	0.0068310000	-35.2231860000	23.2494830000
H78	-1.5913180000	-32.8409420000	18.6844020000
H79	-1.2273700000	-26.6531720000	23.6938570000
H80	-0.0380910000	-27.6850190000	25.6391870000
H81	-0.3294390000	-26.2276290000	26.6038630000
H82	-8.5340140000	-24.7353360000	23.8670290000
H83	-6.6367200000	-23.3891660000	25.8628430000
H84	-8.3631770000	-23.6232600000	26.0936740000
H85	3.6379550000	-34.0710140000	28.5769100000
H86	1.2786110000	-34.6955600000	28.1057800000
H87	1.4002900000	-33.6160470000	29.4922160000
H88	3.7158820000	-33.5038800000	24.0724390000
H89	4.5686490000	-30.5536100000	24.2835030000
H90	4.5553560000	-31.4443570000	22.7766270000
H91	-7.9175010000	-37.9399990000	21.2339690000
H92	-5.6608770000	-38.3979190000	22.2347090000
H93	-4.8732900000	-37.6494060000	20.8534890000
H94	-5.9224580000	-33.5964390000	29.3522260000
H95	-4.2342530000	-33.8027530000	28.8683990000
H96	-5.4386790000	-34.9860190000	28.3714850000
H97	-9.9942090000	-29.0878310000	22.4392340000
H98	-8.9594760000	-28.1394750000	23.5047230000
H99	-9.6375210000	-29.6704330000	24.0835990000
H100	-3.0527110000	-31.4626920000	16.8710170000
H101	-3.8256110000	-30.0844840000	17.6741120000
H102	-4.7861650000	-31.5005340000	17.2404420000
H103	-7.6625630000	-32.6009330000	28.7531260000
H104	-8.5012500000	-34.3305130000	27.0837250000
H105	-9.3267740000	-33.0145030000	26.2228490000
H106	-9.8097910000	-33.4561000000	27.8942470000
H107	-0.7991080000	-33.9894370000	20.9938600000
H108	-5.8590130000	-30.4663720000	19.3160760000
H109	-8.0869680000	-30.0004690000	19.2683050000
H110	-8.9465890000	-27.5126720000	20.8590720000

H111	-9.3155020000	-27.9363940000	19.0933530000
H112	-8.1164480000	-30.6890960000	25.2961390000
H113	-3.0831840000	-34.3182910000	26.9535220000
H114	0.2372290000	-36.0041540000	25.6943610000
H115	-7.1885160000	-34.0510600000	24.0864330000
H116	-7.6593730000	-36.4262540000	23.2314450000
H117	-4.0883280000	-35.0543980000	21.4694140000
H118	-0.1922170000	-31.0275230000	21.8752080000
H119	2.2228200000	-31.8911990000	21.6571120000
H120	1.8805630000	-29.9002360000	25.3396490000
H121	4.2145310000	-31.8212760000	26.4527890000
H122	0.8272310000	-32.6696050000	25.5555480000
H123	-0.5801570000	-32.1608660000	29.4666730000
H124	-2.0273150000	-30.7111000000	27.9050370000
H125	-4.3424290000	-29.6806410000	25.9372810000
H126	-3.2819700000	-25.7206040000	26.9856510000
H127	-5.1042010000	-25.2979660000	25.7825810000
H128	-8.7329560000	-28.2638950000	27.3212010000
H129	-9.2638750000	-25.9785160000	26.5277080000
H130	-9.1678420000	-30.9397740000	28.3023740000
H131	3.2712140000	-32.3595430000	28.3144150000
H132	-1.4218280000	-25.2221490000	24.5787910000
H133	0.0891170000	-25.7121240000	24.1996730000
H134	-8.0232220000	-23.1343920000	23.7901120000
N135	-6.6055630000	-24.4864730000	23.2911010000
H136	5.2636750000	-32.9382860000	24.4987600000
H137	-7.1452560000	-37.1697350000	19.8328300000
H138	-6.9169920000	-38.9075240000	20.1313690000
H139	-4.7201380000	-25.0410250000	21.7138540000
C140	-5.5860060000	-25.6409930000	21.3980540000
C141	-6.8072300000	-25.3327450000	22.2345120000
O142	-7.9026580000	-25.8435920000	22.0106140000
C143	-5.2368040000	-27.1432530000	21.4749890000
C144	-4.0122940000	-27.4505940000	20.6083180000
C145	-5.0299250000	-27.6096290000	22.9231680000
H146	-5.7137420000	-24.0213490000	23.3780800000
H147	-5.8137070000	-25.3727460000	20.3594860000
H148	-6.0986670000	-27.6859950000	21.0738240000
H149	-3.1387060000	-26.8788620000	20.9556060000
H150	-4.1901990000	-27.1773800000	19.5599630000
H151	-3.7416530000	-28.5095370000	20.6481610000
H152	-5.8952900000	-27.3922630000	23.5572410000
H153	-4.1456780000	-27.1394750000	23.3773120000
H154	-4.8915290000	-28.6929230000	22.9279500000
H155	-2.3552890000	-30.1821420000	22.0295330000
C156	-11.0220230000	-37.0394780000	21.4210240000
C157	-10.4400210000	-36.8092310000	22.8036670000
O158	-9.3329940000	-37.2180020000	23.1570840000
N159	-11.2626260000	-36.1473310000	23.6578370000
C160	-10.8110120000	-35.7070310000	24.9628930000

H161	-10.3616630000	-37.6860960000	20.8435290000
H162	-11.1652890000	-36.0931090000	20.8883210000
H163	-12.1172370000	-35.7529750000	23.2916090000
H164	-10.0099890000	-34.9620930000	24.8772350000
H165	-11.6523040000	-35.2513470000	25.4907230000
H166	-11.9911060000	-37.5204580000	21.5567840000
H167	-10.4290510000	-36.5298500000	25.5672420000
O168	-1.8211620000	-29.8471790000	21.2470340000
O169	-3.0524050000	-30.4888530000	23.5119460000
H170	-1.4566450000	-29.0184880000	21.5944080000
H171	-3.5702990000	-29.6843090000	23.6525250000
H172	-7.0570810000	-29.8464280000	27.6503370000

I$_P$ (singlet, +1)

C1	-0.8190000000	-26.0150010000	24.4890000000
C2	-0.7258540000	-26.7272590000	25.8624990000
C3	-2.0694930000	-27.2698730000	26.2210670000
N4	-2.4564000000	-28.5444320000	25.8198990000
C5	-3.1588220000	-26.6304650000	26.7307570000
C6	-3.7407130000	-28.6782210000	26.0810390000
N7	-4.2284550000	-27.5206510000	26.6371670000
C8	-7.7229990000	-24.0380000000	24.0960010000
C9	-7.4927970000	-24.0217250000	25.6265300000
C10	-7.2124930000	-25.3928700000	26.1795010000
C11	-8.2526170000	-26.2977750000	26.5254150000
C12	-5.8903010000	-25.8346880000	26.2507020000
C13	-7.9752120000	-27.5837100000	26.9254990000
C14	-5.5755050000	-27.1241740000	26.6469730000
C15	-6.6161470000	-28.0733530000	27.0210650000
O16	-6.3316510000	-29.2404920000	27.4071960000
C17	2.9850000000	-33.3740010000	28.0370010000
C18	3.1619060000	-33.5706210000	26.5313260000
O19	2.6407180000	-34.5026400000	25.9243850000
C20	1.5064690000	-33.6417540000	28.3965020000
C21	0.5421380000	-32.6867260000	27.7421880000
N22	-0.4455940000	-32.0543670000	28.4773120000
C23	0.3396810000	-32.2435570000	26.4544350000
C24	-1.1885290000	-31.2791540000	27.6507280000
N25	-0.7268670000	-31.3637470000	26.4176480000
N26	3.8926690000	-32.5993310000	25.9114760000
C27	4.2949990000	-32.6899990000	24.5160010000
C28	4.0565920000	-31.3716870000	23.7452260000
C29	2.6163690000	-30.9855250000	23.6314300000
N30	1.7430060000	-31.5530990000	22.7141080000
C31	1.8358190000	-30.1217350000	24.3565770000
C32	0.5048280000	-31.0270710000	22.9107840000
N33	0.5296980000	-30.1550520000	23.9055920000
C34	-6.8306390000	-37.7512700000	20.5502620000

C35	-5.6485370000	-37.4503210000	21.4889430000
C36	-5.7101740000	-36.0834730000	22.0963500000
N37	-6.7941940000	-35.6372680000	22.8359950000
C38	-4.8223930000	-35.0394780000	22.0957320000
C39	-6.5402810000	-34.3809550000	23.2518200000
N40	-5.3480030000	-33.9892010000	22.8247660000
Cu41	-1.2215070000	-29.8811260000	25.0218410000
Fe42	-4.4248260000	-32.2652660000	23.3162780000
C43	-1.6566100000	-33.3941540000	21.6144240000
C44	-3.3971290000	-33.9013330000	26.1223110000
C45	-7.2303450000	-31.1791740000	24.9855380000
C46	-5.6027960000	-30.8569870000	20.4477370000
N47	-6.0881360000	-31.2299880000	22.8175540000
C48	-1.7839490000	-33.7667350000	22.9533210000
C49	-0.8775520000	-34.6234970000	23.6480410000
C50	-1.3690000000	-34.7890010000	24.9249990000
C51	-2.5835760000	-33.9974520000	24.9946900000
C52	-0.6577780000	-35.4901700000	25.9930340000
O53	-1.0106090000	-35.5648420000	27.1640830000
N54	-5.1572370000	-32.4587630000	25.1979500000
C55	-4.5921360000	-33.2105600000	26.2006050000
C56	-5.4691950000	-33.2432060000	27.3618930000
C57	-6.5816410000	-32.5179790000	27.0298920000
C58	-6.3533720000	-32.0091020000	25.6822190000
C59	-5.1715470000	-33.9936870000	28.6257780000
N60	-2.8122790000	-33.3947450000	23.7900920000
C61	-7.0888110000	-30.8018110000	23.6589370000
C62	-8.0460360000	-29.9808060000	22.9377920000
C63	-7.6380400000	-29.9742260000	21.6240600000
C64	-6.3935360000	-30.7353550000	21.5798680000
C65	-9.2318930000	-29.2889230000	23.5307310000
C66	-8.2880910000	-29.4135480000	20.4409680000
C67	-9.0167880000	-28.2885360000	20.3817420000
N68	-3.7453460000	-32.1285130000	21.4200610000
C69	-4.3677100000	-31.4908050000	20.3843520000
C70	-3.5553180000	-31.5561260000	19.1767880000
C71	-2.4416990000	-32.2630690000	19.5192640000
C72	-2.5677010000	-32.6230510000	20.9118370000
C73	-3.9125820000	-30.9541680000	17.8524420000
C74	-7.8691860000	-32.4035910000	27.8147750000
O75	-8.4486860000	-31.1010860000	27.7595690000
C76	-8.9034390000	-33.4083980000	27.2762150000
H77	0.0308680000	-35.0486300000	23.2368640000
H78	-1.6071010000	-32.5334970000	18.8825840000
H79	-1.2253070000	-26.7029360000	23.7380980000
H80	-0.0041200000	-27.5517900000	25.8161450000
H81	-0.3744320000	-26.0218470000	26.6255910000
H82	-8.5282930000	-24.7414090000	23.8672600000
H83	-6.6674290000	-23.3404580000	25.8723550000
H84	-8.3974740000	-23.6017630000	26.0839880000

H85	3.6316200000	-34.0835300000	28.5660170000
H86	1.2518010000	-34.6648750000	28.1008760000
H87	1.3855540000	-33.5856860000	29.4853650000
H88	3.7140780000	-33.5084880000	24.0827730000
H89	4.5969510000	-30.5567070000	24.2429560000
H90	4.5097060000	-31.4696900000	22.7489200000
H91	-7.7781480000	-37.7064860000	21.0981390000
H92	-5.6178580000	-38.2071460000	22.2857910000
H93	-4.7004420000	-37.5353320000	20.9453050000
H94	-5.8663510000	-33.7237400000	29.4264370000
H95	-4.1542600000	-33.7870100000	28.9802670000
H96	-5.2410450000	-35.0792010000	28.4770410000
H97	-10.0423690000	-29.2247370000	22.7968790000
H98	-8.9749910000	-28.2596470000	23.8143370000
H99	-9.6089860000	-29.7984100000	24.4233650000
H100	-3.1299620000	-31.1505660000	17.1136720000
H101	-4.0415120000	-29.8662170000	17.9257830000
H102	-4.8532370000	-31.3637680000	17.4621520000
H103	-7.6530100000	-32.6640550000	28.8636360000
H104	-8.4653180000	-34.4122920000	27.2412550000
H105	-9.1904550000	-33.1186100000	26.2591250000
H106	-9.8097580000	-33.4560620000	27.8942110000
H107	-0.7907900000	-33.7672920000	21.0731930000
H108	-5.9615020000	-30.3883300000	19.5387860000
H109	-8.1601400000	-29.9932350000	19.5259380000
H110	-9.1276910000	-27.6213170000	21.2260970000
H111	-9.4781140000	-27.9810370000	19.4465080000
H112	-8.0950270000	-30.8288970000	25.5328080000
H113	-3.0569890000	-34.4474450000	26.9931340000
H114	0.3008280000	-35.9448780000	25.6713180000
H115	-7.2113470000	-33.7835040000	23.8483390000
H116	-7.6663820000	-36.1500280000	22.9961490000
H117	-3.8502250000	-34.9733660000	21.6371000000
H118	-0.3757700000	-31.3097950000	22.3548200000
H119	1.9832650000	-32.2400170000	22.0133530000
H120	2.1244450000	-29.4979690000	25.1893580000
H121	4.3562050000	-31.9099850000	26.4878200000
H122	0.8723440000	-32.5072360000	25.5566710000
H123	-0.5801620000	-32.1608920000	29.4668080000
H124	-2.0265420000	-30.6751980000	27.9663160000
H125	-4.3481450000	-29.5356100000	25.8393100000
H126	-3.2769050000	-25.6479360000	27.1578270000
H127	-5.0981860000	-25.1683310000	25.9273660000
H128	-8.7635580000	-28.2852630000	27.1830960000
H129	-9.2853430000	-25.9632760000	26.4596600000
H130	-7.7158520000	-30.4514600000	27.8051010000
H131	3.2712730000	-32.3595090000	28.3144090000
H132	-1.4217630000	-25.2221430000	24.5787600000
H133	0.0890980000	-25.7122920000	24.1996460000
H134	-8.0231320000	-23.1344800000	23.7901080000

N135	-6.5930730000	-24.4787430000	23.3042050000
H136	5.2635440000	-32.9382590000	24.4989890000
H137	-6.8719330000	-37.0249750000	19.7304290000
H138	-6.7336490000	-38.7530710000	20.1178020000
H139	-4.7162010000	-25.0401420000	21.6991080000
C140	-5.5860000000	-25.6410010000	21.3980010000
C141	-6.7903000000	-25.3684600000	22.2781410000
O142	-7.8563510000	-25.9576970000	22.1252560000
C143	-5.2436170000	-27.1522480000	21.4725070000
C144	-4.0241100000	-27.4881340000	20.6077270000
C145	-5.0299140000	-27.6096400000	22.9231870000
H146	-5.7615790000	-23.9041730000	23.2828810000
H147	-5.8422810000	-25.3695300000	20.3670770000
H148	-6.1148260000	-27.6869660000	21.0789410000
H149	-3.1199440000	-26.9906330000	20.9868160000
H150	-4.1636520000	-27.1732790000	19.5659770000
H151	-3.8435900000	-28.5706290000	20.6062300000
H152	-5.9157960000	-27.4278950000	23.5407260000
H153	-4.1827260000	-27.0809570000	23.3864990000
H154	-4.8250660000	-28.6851410000	22.9754700000
H155	-2.7510450000	-29.5688650000	22.6734350000
C156	-11.0219990000	-37.0400010000	21.4210000000
C157	-10.4156210000	-36.7150760000	22.7694110000
O158	-9.2334280000	-36.9390830000	23.0560210000
N159	-11.2622370000	-36.1524770000	23.6592860000
C160	-10.8110000000	-35.7070010000	24.9629990000
H161	-10.3583070000	-37.7096100000	20.8722530000
H162	-11.1664800000	-36.1245160000	20.8352790000
H163	-12.2202230000	-35.9842300000	23.3879240000
H164	-10.0099640000	-34.9658160000	24.8645060000
H165	-11.6484180000	-35.2443500000	25.4896220000
H166	-11.9911550000	-37.5200200000	21.5567300000
H167	-10.4290980000	-36.5299030000	25.5672210000
O168	-2.4404560000	-30.3537330000	23.1598720000
O169	-3.6611920000	-30.7214830000	23.8719860000

I_{PR} (doublet, 0)

C1	-0.8191190000	-26.0152130000	24.4889680000
C2	-0.7603400000	-26.8054710000	25.8187370000
C3	-2.1019470000	-27.3900220000	26.1347010000
N4	-2.4680850000	-28.6573030000	25.7095110000
C5	-3.2107210000	-26.7683750000	26.6409530000
C6	-3.7739990000	-28.7857420000	25.9455640000
N7	-4.2722910000	-27.6494920000	26.4950980000
C8	-7.7229490000	-24.0381280000	24.0958730000
C9	-7.4721070000	-24.0610260000	25.6125720000
C10	-7.2298170000	-25.4494990000	26.1439990000
C11	-8.2630820000	-26.3409750000	26.4869750000

C12	-5.9322080000	-25.9460410000	26.1799600000
C13	-7.9989640000	-27.6544610000	26.8515620000
C14	-5.6468380000	-27.2556380000	26.5558170000
C15	-6.6713510000	-28.1998730000	26.9036940000
O16	-6.4112040000	-29.4187760000	27.2186600000
C17	2.9849900000	-33.3739660000	28.0369610000
C18	3.1934080000	-33.5898930000	26.5400660000
O19	2.7627050000	-34.5794790000	25.9547690000
C20	1.5147700000	-33.6564480000	28.4069790000
C21	0.5403560000	-32.7201160000	27.7515830000
N22	-0.4558160000	-32.0824320000	28.4709640000
C23	0.3298060000	-32.3227040000	26.4513510000
C24	-1.2134430000	-31.3476350000	27.6127890000
N25	-0.7525520000	-31.4692080000	26.3824600000
N26	3.8700460000	-32.5891270000	25.9036810000
C27	4.2949770000	-32.6900140000	24.5159890000
C28	4.0477420000	-31.3865870000	23.7266810000
C29	2.6029840000	-31.0376680000	23.5808930000
N30	1.7557510000	-31.6676770000	22.6781250000
C31	1.7900000000	-30.1768820000	24.2729430000
C32	0.4987350000	-31.1728730000	22.8497310000
N33	0.4904570000	-30.2704580000	23.8154350000
C34	-6.9586110000	-37.9014780000	20.4976350000
C35	-5.7391420000	-37.5953830000	21.3851260000
C36	-5.7730480000	-36.2264370000	21.9889550000
N37	-6.8343120000	-35.7702980000	22.7555850000
C38	-4.8793040000	-35.1871600000	21.9610480000
C39	-6.5644160000	-34.5088730000	23.1509110000
N40	-5.3826260000	-34.1281470000	22.6895310000
Cu41	-1.2482950000	-30.0133080000	24.8982110000
Fe42	-4.4317590000	-32.3970180000	23.1586230000
C43	-1.6203610000	-33.5588610000	21.5440290000
C44	-3.4555240000	-33.9497030000	26.0312580000
C45	-7.2244330000	-31.2054770000	24.7711870000
C46	-5.5270920000	-31.0201660000	20.2462520000
N47	-6.0589860000	-31.3456460000	22.6147120000
C48	-1.7698940000	-33.8861690000	22.8945470000
C49	-0.8491310000	-34.6667790000	23.6505970000
C50	-1.3690290000	-34.7889940000	24.9250050000
C51	-2.6175260000	-34.0594290000	24.9232520000
C52	-0.6669060000	-35.4288370000	26.0300640000
O53	-1.0521020000	-35.5167620000	27.1916430000
N54	-5.1995870000	-32.5454060000	25.0313490000
C55	-4.6543890000	-33.2669940000	26.0652810000
C56	-5.5411700000	-33.2493820000	27.2179160000
C57	-6.6336610000	-32.5120700000	26.8600810000
C58	-6.3835730000	-32.0467950000	25.4972590000
C59	-5.2573100000	-33.9572140000	28.5085200000
N60	-2.8366870000	-33.5227580000	23.6872010000
C61	-7.0496780000	-30.8623070000	23.4398230000

C62	-7.9575410000	-30.0025940000	22.6956770000
C63	-7.5371880000	-30.0391860000	21.3869780000
C64	-6.3269710000	-30.8544690000	21.3675840000
C65	-9.1008000000	-29.2349130000	23.2789500000
C66	-8.1441620000	-29.4534050000	20.1924650000
C67	-8.7574230000	-28.2631800000	20.1123870000
N68	-3.7133640000	-32.3119320000	21.2721330000
C69	-4.3039690000	-31.6813430000	20.2150670000
C70	-3.4661010000	-31.7789460000	19.0266400000
C71	-2.3665610000	-32.4890370000	19.4049130000
C72	-2.5250050000	-32.8202290000	20.8021450000
C73	-3.7865730000	-31.1898140000	17.6875360000
C74	-7.9094370000	-32.3274730000	27.6559910000
O75	-8.4843800000	-31.0478130000	27.5120870000
C76	-8.9405970000	-33.3934400000	27.2215810000
H77	0.0887960000	-35.0695460000	23.2859340000
H78	-1.5174130000	-32.7719610000	18.7934740000
H79	-1.2256370000	-26.6696950000	23.7075370000
H80	-0.0289320000	-27.6190810000	25.7339610000
H81	-0.4241820000	-26.1448690000	26.6286510000
H82	-8.5406060000	-24.7300490000	23.8736360000
H83	-6.6233290000	-23.4042070000	25.8574810000
H84	-8.3536240000	-23.6064030000	26.0859910000
H85	3.6382250000	-34.0749130000	28.5707510000
H86	1.2704470000	-34.6857530000	28.1221370000
H87	1.4038640000	-33.5947300000	29.4965340000
H88	3.7219290000	-33.5155750000	24.0857350000
H89	4.5603380000	-30.5542770000	24.2252670000
H90	4.5234460000	-31.4879230000	22.7411610000
H91	-7.8815970000	-37.8602330000	21.0852830000
H92	-5.6734940000	-38.3518830000	22.1804050000
H93	-4.8164110000	-37.6820140000	20.8002170000
H94	-5.9611950000	-33.6578870000	29.2901380000
H95	-4.2442160000	-33.7358020000	28.8658200000
H96	-5.3245800000	-35.0476490000	28.3983710000
H97	-9.8636540000	-29.0429250000	22.5173320000
H98	-8.7609240000	-28.2613580000	23.6561320000
H99	-9.5636670000	-29.7605270000	24.1201760000
H100	-2.9949050000	-31.4098970000	16.9643020000
H101	-3.8965520000	-30.0991230000	17.7450490000
H102	-4.7284800000	-31.5852260000	17.2859210000
H103	-7.6644690000	-32.5164150000	28.7166540000
H104	-8.4712730000	-34.3838040000	27.1898840000
H105	-9.2988650000	-33.1433560000	26.2162110000
H106	-9.8097210000	-33.4560390000	27.8941990000
H107	-0.7330600000	-33.9283290000	21.0365730000
H108	-5.8581720000	-30.5508250000	19.3269310000
H109	-8.0834430000	-30.0677600000	19.2924100000
H110	-8.7914390000	-27.5611550000	20.9382670000
H111	-9.1984790000	-27.9362140000	19.1736790000

H112	-8.0671300000	-30.7963960000	25.3101630000
H113	-3.1223790000	-34.4596670000	26.9262120000
H114	0.3313490000	-35.8258320000	25.7497980000
H115	-7.2158930000	-33.9037280000	23.7611010000
H116	-7.6931760000	-36.2844090000	22.9631580000
H117	-3.9168960000	-35.1252170000	21.4796330000
H118	-0.3708050000	-31.4969640000	22.2977310000
H119	2.0200110000	-32.3750110000	22.0077740000
H120	2.0484280000	-29.5128690000	25.0848870000
H121	4.2430310000	-31.8314300000	26.4583300000
H122	0.8626040000	-32.6104470000	25.5601940000
H123	-0.5801560000	-32.1608850000	29.4668030000
H124	-2.0591200000	-30.7420730000	27.9049420000
H125	-4.3810310000	-29.6431220000	25.7100050000
H126	-3.3450010000	-25.7914010000	27.0761170000
H127	-5.1168730000	-25.3035770000	25.8573860000
H128	-8.8134500000	-28.3242650000	27.1160790000
H129	-9.2965240000	-25.9955500000	26.4571880000
H130	-7.7270670000	-30.3793510000	27.5027600000
H131	3.2712870000	-32.3595350000	28.3144470000
H132	-1.4216850000	-25.2220750000	24.5787510000
H133	0.0891230000	-25.7121660000	24.1996900000
H134	-8.0231420000	-23.1343920000	23.7901710000
N135	-6.6114480000	-24.4906980000	23.2825680000
H136	5.2635540000	-32.9382510000	24.4990040000
H137	-7.0373990000	-37.1740760000	19.6815680000
H138	-6.8771230000	-38.9030300000	20.0604990000
H139	-4.7054280000	-25.0766770000	21.7374880000
C140	-5.5860380000	-25.6410140000	21.3981090000
C141	-6.8205250000	-25.2812180000	22.1954630000
O142	-7.9359760000	-25.6921040000	21.8761980000
C143	-5.2918560000	-27.1589390000	21.4805070000
C144	-4.1160450000	-27.5422780000	20.5725570000
C145	-5.0299120000	-27.6096080000	22.9231470000
H146	-5.6750930000	-24.1848560000	23.5007820000
H147	-5.7769450000	-25.3614710000	20.3554380000
H148	-6.1873360000	-27.6733260000	21.1179280000
H149	-3.1876200000	-27.0541710000	20.9023400000
H150	-4.2946810000	-27.2489430000	19.5303040000
H151	-3.9557730000	-28.6278630000	20.5887550000
H152	-5.8700610000	-27.3867840000	23.5884760000
H153	-4.1425830000	-27.1107850000	23.3401170000
H154	-4.8609930000	-28.6918390000	22.9776840000
H155	-2.7940360000	-29.5896020000	22.6443960000
C156	-11.0219830000	-37.0399930000	21.4210190000
C157	-10.4292110000	-36.7552990000	22.7835500000
O158	-9.2701620000	-37.0559010000	23.0884060000
N159	-11.2648790000	-36.1571580000	23.6605910000
C160	-10.8109940000	-35.7070050000	24.9629860000
H161	-10.3594140000	-37.7018320000	20.8629260000

H162	-11.1638570000	-36.1116520000	20.8560910000
H163	-12.1941870000	-35.9079900000	23.3545320000
H164	-10.0065980000	-34.9697880000	24.8640580000
H165	-11.6456480000	-35.2398530000	25.4878010000
H166	-11.9911680000	-37.5200230000	21.5567250000
H167	-10.4291010000	-36.5299070000	25.5672230000
O168	-2.4769940000	-30.4496170000	22.9759710000
O169	-3.6462430000	-30.8778800000	23.7356850000
